# Coevolution of marine phytoplankton and *Alteromonas* bacteria in response to pCO_2_ and coculture

**DOI:** 10.1093/ismejo/wrae259

**Published:** 2024-12-24

**Authors:** Zhiying Lu, Elizabeth Entwistle, Matthew D Kuhl, Alexander R Durrant, Marcelo Malisano Barreto Filho, Anuradha Goswami, J Jeffrey Morris

**Affiliations:** Department of Biology, University of Alabama at Birmingham, Birmingham, AL 35294, United States; Department of Biology, University of Alabama at Birmingham, Birmingham, AL 35294, United States; Department of Biology, University of Alabama at Birmingham, Birmingham, AL 35294, United States; Department of Biology, University of Alabama at Birmingham, Birmingham, AL 35294, United States; Department of Biology, University of Alabama at Birmingham, Birmingham, AL 35294, United States; Department of Biology, University of Alabama at Birmingham, Birmingham, AL 35294, United States; Department of Biology, University of Alabama at Birmingham, Birmingham, AL 35294, United States

**Keywords:** phytoplankton, experimental evolution, ocean acidification, coevolution

## Abstract

As a result of human activity, Earth’s atmosphere and climate are changing at an unprecedented pace. Models based on short-term experiments predict major changes will occur in marine phytoplankton communities in the future ocean, but rarely consider how evolution or interactions with other microbes may influence these changes. Here, we experimentally evolved several phytoplankton in coculture with a heterotrophic bacterium, *Alteromonas* sp. EZ55, under either present-day or predicted future pCO_2_ conditions. Growth rates of phytoplankton generally increased over time under both conditions, but only *Thalassiosira oceanica* had evidence of a growth rate tradeoff in the ancestral environment after evolution at elevated pCO_2_. The growth defects observed in ancestral *Prochlorococcus* cultures at elevated pCO_2_ and in axenic culture were diminished after evolution, possibly due to regulatory mutations in antioxidant genes. Except for *Prochlorococcus*, mutational profiles suggested phytoplankton experienced primarily purifying selection, but most *Alteromonas* lineages showed evidence of directional selection, where evolution appeared to favor a metabolic switch between growth on small organic acids with cyanobacteria versus catabolism of more complex carbon substrates with eukaryotic phytoplankton. Evolved *Alteromonas* were also poorer “helpers” for *Prochlorococcus*, consistent with that interaction being a competitive Black Queen process rather than a true mutualism. This work provides new insights on how phytoplankton will respond to increased pCO_2_ and on the evolutionary mechanisms governing phytoplankton:bacteria interactions. It also clearly demonstrates that both evolution and interspecies interactions must be considered to predict future marine biogeochemistry.

## Introduction

As a result of human fossil fuel use, Earth’s atmospheric pCO_2_ has increased by ~40% since the industrial revolution and is projected to further double by the end of the century [1]. Elevated pCO_2_ affects ocean biogeochemistry primarily by raising ocean temperature and lowering ocean pH, and marine ecosystems may experience extensive compositional changes as organisms better adapted to the new conditions locally replace those that are currently dominant [2, 3]. For example, models informed by short-term culture experiments have predicted that many smaller phytoplanktons such as cyanobacteria will become more abundant in warmer and/or more acidic oceans [4, 5]. In contrast, the cyanobacterium *Prochlorococcus*, which is numerically dominant in current oligotrophic oceans, has shown pronounced reductions in growth rate at elevated pCO_2_ [6–8]. However, making predictions about future seas from pure culture experiments is complicated by their lack of community context. *Prochlorococcus*, for instance, displays different responses to increased pCO_2_ depending on whether it is in axenic culture, in coculture with heterotrophic [6, 9] or other photosynthetic microbes [10], or in *in situ* communities [11]. Heterotrophic bacteria in general have profound impacts on the metabolisms of phytoplankton in culture experiments [12–17], and very little is known about how anthropogenic change will impact pelagic bacterial assemblages, or how those changes may reverberate through the phytoplankton. Moreover, ecological and evolutionary time scales overlap for fast-growing microbes [18], and therefore natural selection will act on populations to adapt them to changing environments [19], potentially mitigating negative impacts of environmental change [20].

Although several studies of phytoplankton responses to future pCO_2_ have considered long-term evolutionary responses [21–31], to our knowledge, no study has observed coevolution between phytoplankton and heterotrophic bacteria under simulated global change conditions such as increased pCO_2_. We therefore sought to explore how the growth rates, metabolisms, and genomes of simple assemblages of marine phytoplankton and heterotrophic bacteria adapt to ocean acidification using long-term experimental evolution. We grew a variety of unicellular phytoplankton in low-density semicontinuous cultures either under current atmospheric pCO_2_ conditions or projected year 2100 conditions (400 vs. 800 ppm, respectively) for ~500 generations. We selected taxa representing major ecological functional groups: the open ocean diatom *Thalassiosira oceanica* CCMP1005, the important bloom-forming coccolithophore *Emiliania huxleyi* CCMP371, the coastal picocyanobacterium *Synechococcus* CC9311, and the highly abundant oligotrophic picocyanobacterium *Prochlorococcus* MIT9312. These groups are ecologically important not only just because they interact with ocean biogeochemistry in distinct ways (e.g. *Prochlorococcus* as an oligotrophic specialist, *T. oceanica* and *E. huxleyi* as silicifiers and calcifiers, respectively) but also because they have conspicuously different growth rate responses to elevated pCO_2_ in short-term culture experiments (i.e. positive for *Synechococcus,* negative for *Prochlorococcus*, and no net effect for diatoms or coccolithophores) [4].

In addition to these photoautotrophic taxa, each culture also included a single heterotrophic bacterial strain, the Gammaproteobacterium *Alteromonas macleodii* EZ55. Strains of *Alteromonas* are commonly found inhabiting phytoplankton cultures [32, 33] and are ubiquitous in ocean waters worldwide [34]. The decision to use mixed instead of axenic cultures was made for two reasons. First, the bacteria facilitated carbon cycling of photosynthetic exudates, preventing the environmentally unrealistic overaccumulation of metabolites in the cultures that might obscure any evolutionary responses of the phytoplankton to pCO_2_ manipulation [10, 35]. Second, *Prochlorococcus* grew very poorly in axenic culture especially under elevated pCO_2_ conditions [6, 7, 36], and in preliminary experiments, we were unable to sustain axenic *Prochlorococcus* in semicontinuous low-density cultures for more than a few transfers. Whereas some *Alteromonas* strains are known to have negative impacts on *Prochlorococcus* [16], we have used EZ55 many times as a “helper” for *Prochlorococcus* cultures [6–8, 36, 37], and therefore chose it as our heterotrophic component in these experiments.

## Materials and methods

### Cultures and media

The phytoplankton used in this study as well as the media in which they were grown are listed in Table S1. We chose these particular strains because they represented major functional groups of phytoplankton often represented in ocean ecology models [38]. The particular strains of *Prochlorococcus* and *Synechococcus* were chosen because they are both surface mixed layer inhabitants and because they demonstrated opposite responses to elevated pCO_2_ in preliminary studies [8]. *T. oceanica* was chosen over other more commonly studied diatoms with finished genomes such as *T. pseudonana* because it is more abundant in the ocean [39]. Finally, we chose the CCMP371 strain of *E. huxleyi* because it is more strongly calcified than the one strain, CCMP1516, with a finished genome [40], and thus was more likely to respond strongly to changes in pCO_2_.

All media types were derived from media commonly used to cultivate each organism [9]. P concentrations were standardized at 2 μM NaH_2_PO_4_ across all media types, with N added at Redfield proportions (16:1 N:P). *Prochlorococcus* was grown in PEv medium, which was a 1/25 dilution of Pro99 (32 μM NH_4_Cl, 40 μl l^−1^ Pro99 trace metals). *Synechococcus* and *Synechocystis* were grown in SEv medium, which was modeled on SN, but with N concentrations lowered to be at Redfield proportions with P (final concentrations 32 μM NaNO_3_, 20 μl l^−1^ SN trace metals, 20 μl F/2 vitamins). *T. oceanica* and *E. huxleyi* were grown in FEv medium, which was a 1/25 dilution of F/2 medium, replacing the F/2 trace metal solution with 40 μl l^−1^ of L1 trace metals. All media were prepared in an artificial seawater (ASW) base (per liter, 28.4 g NaCl, 7.21 g MgSO_4_*7H_2_O, 5.18 g MgCl_2_*6H_2_O, 1.58 g CaCl_2_, 0.79 g KCl). This ASW base was autoclaved in 1 or 2 l batches, then filter-sterilized nutrient solutions and 4 ml of filter-sterilized ~0.58 M NaHCO_3_ solution were added for each liter of media. The precise concentration of NaHCO_3_ of each batch was determined by titration (see below). Completed media were bubbled for at least 24 h with sterile air to equilibrate the carbonate system with the atmosphere. All media storage bottles and culture glassware were acid washed prior to use.

Prior to use in experiments, phytoplankton cultures were rendered clonal and axenic, eliminating nearly all genetic variation from starting populations. First, cultures were diluted to ~1 cell ml^−1^ and then 100 μl aliquots were distributed to 96-well plates, such that each well was likely to have either 0 or 1 cells; replicate ancestral cultures of each strain were picked from these plates, cryopreserved (see below) and used for all subsequent experiments. We used a streptomycin-resistant *Prochlorococcus* strain [37] and each of our six ancestral cultures was rendered axenic according to [36]. *Synechococcus* and *Synechocystis* were made axenic by dilution to extinction in late exponential phase batch cultures, where phytoplankton cells outnumbered bacteria; these cultures were then diluted into 96-well microtiter plates at concentrations that effectively excluded the bacteria. *T. oceanica* and *E. huxleyi* were both axenic upon receipt from the National Collection of Marine Algae (Boothbay Harbor, ME). Putatively axenic cultures were checked for bacterial contamination by adding 1 ml aliquots to liquid YTSS medium [41]; if no growth was observed after 48 h, the cultures were diluted 100-fold into fresh medium to initiate experiments.

After our pilot experiment using axenic *Synechocystis* (see below), we chose to incorporate the heterotrophic bacterium *Alteromonas* sp. EZ55 into our other cultures. We made the decision to use bacteria for several reasons stated in the introduction, and we chose an *Alteromonas* strain over taxa more commonly associated with phytoplankton because of our extensive use of EZ55 as a “helper” for *Prochlorococcus* cultures [6–8, 36, 37]. *Alteromonas* sp. EZ55 was streaked for isolation on YTSS agar and individual colonies were used to inoculate clonal liquid YTSS cultures. Prior to addition to axenic phytoplankton, *Alteromonas* cells were pelleted by centrifugation (2000×*g* for 2 min) and washed twice with sterile ASW. *Alteromonas* clones were reisolated from postevolution cultures by spread plating on YTSS agar.

### Carbonate system manipulation

We manipulated the carbonate system in our cultures by careful additions of HCl, NaHCO_3_, and/or NaOH [42]. The exact concentration of each solution was determined by titrating the alkalinity of ASW before and after the addition of gravimetrically determined masses of solution. Alkalinity titrations were performed with factory-standardized 0.1 N HCl using a Mettler Toledo T5 titrator according to [43]. Medium pH was determined by addition of m-cresol purple according to [43] but with the protocol modified to use a BioTek Synergy H1 plate reader instead of a standard spectrophotometer. Using the measured pH and alkalinity of a batch of media, we calculated the additions of either HCl and NaHCO_3_, or NaOH, necessary to achieve 400 ppm or 800 ppm pCO_2_ conditions using the package *seacarb* in R [44]. We used 0.2 M NaHCO_3_, 0.2 M HCl, and 0.1 M NaOH solutions for carbonate system manipulation. All solutions were filter sterilized prior to calibration by titration and were monitored for bacterial contamination by periodically adding aliquots to YTSS broth.

In general, we only assessed the alkalinity and atmosphere-equilibrated pH of a 2 l batch of media once, prior to its utilization for experiments. Alkalinity was very stable over repeat measurements and was close to the target value of 2.32 mM in all batches (Fig. S20A). After supplementing media according to our *seacarb* analysis, pH was also close to our target values (Fig. S20B), although our calculated pCO_2_ values for year 2100 cultures tended to be higher than our target value of 800 ppm (Fig. S20C). However, ~1 year into the experiment, we discovered that the atmospheric pCO_2_ in the laboratory varied between ~380 and 450 ppm, which led to some drift in pCO_2_ concentrations in the media over the period necessary to consume an entire 2 l batch. We therefore began monitoring laboratory CO_2_ levels and reassessed pH (and recalculated necessary additions) approximately monthly. Most pH deviations in media batches that were tested multiple times were less than 0.1 pH unit, and none were high enough to result in overlap between our ambient and year 2100 pCO_2_ treatment groups (Fig. S21). Because of time and media volume limitations, we were unable to measure alkalinity and pH continually during the experiment so it is possible that larger deviations occurred of which we are unaware.

### Cryopreservation of cultures

Using dilution-to-extinction, we isolated five (PCC6803) or six clones (all other strains) of each phytoplankton strain to initiate evolution experiments. We also isolated six *Alteromonas* clones from isolated colonies on YTSS agar. These clonal populations represent the ancestors of our experiment and were each cryopreserved immediately. *Alteromonas* clones were frozen at −80°C in YTSS with 20% sterile glycerol. Cyanobacteria were flash frozen in culture media with 7.5% sterile DMSO by immersion in liquid nitrogen [45]. Eukaryotic phytoplankton was also treated with 7.5% DMSO, but were slowly frozen using a Thermo Scientific Mr. Frosty device according to the manufacturer’s instructions [9]. After the freezing process, all cultures were placed in long-term storage in liquid nitrogen vapor.

When it was necessary to recover *Alteromonas* from frozen stocks, *Alteromonas* cultures were revived by scraping some frozen material from the top of the sample with a sterile wooden dowel and streaking for isolation onto YTSS agar; new experiments were always initiated from fresh clonal isolates. Phytoplankton was revived by placing frozen samples into room temperature water in the dark just long enough to fully thaw. Then, working under very low light (< 5 μmol photon m^−2^ s^−1^ direct illumination), 500 μl were inoculated into fresh media, and the tube was refrozen using the same techniques described above. Freshly revived cultures were placed into an incubator under very low light (~5 μmol photons m^−2^ s^−1^) for 48 h, then moved to moderate (~30 μmol photons m^−2^ s^−1^) light and monitored for growth by flow cytometry (see below).

### Experimental evolution

Cultures for experimental evolution were initiated with 12.3 ml of media, 0.2 ml of acid, or base additions for carbonate chemistry manipulation, and 0.5 ml of a previous culture. All cultures were grown at 22°C under ~75 μmol photons m^−2^ s^−1^ in acid-washed conical-bottom glass tubes with airtight caps; with 13 ml of culture, almost no headspace existed in these tubes. All cultures except *Prochlorococcus* were grown on a rotating test tube wheel with illumination from the top and bottom; preliminary observations indicated that *Prochlorococcus* cells did not settle noticeably when grown in static test tube racks, whereas all other taxa did. Each phytoplankton clone was split into two culture lines, one maintained at 400 ppm pCO_2_ and the other at 800 ppm pCO_2_. For all strains except *Synechocystis*, each clone was cocultured with a single *Alteromonas* clone. Thus, all phytoplankton designated “1” in Table S1 were cocultured with *Alteromonas* clone 1, all phytoplankton labeled “2” were cocultured with *Alteromonas* clone 2, and so forth. *Synechocystis* was evolved in axenic culture as a pilot experiment during method development because our final results focus heavily on the evolution of algal:bacterial interactions. We present the data from these cultures as part of the online supplementary material.

Phytoplankton growth was measured every 48 h using a Guava HT1 flow cytometer equipped with a 488 nm laser. Phytoplankton populations were identified by their clustering pattern on logarithmic plots of forward light scatter vs. 660 nm (chlorophyll) fluorescence (Fig. S23). Cell densities within user-defined gates encircling the phytoplankton were calculated automatically by the Guava software. When phytoplankton cell densities crossed a cutoff value (Table S1), cultures were diluted 26-fold (0.5 ml into a total volume of 13 ml) into fresh media. The cutoff value was chosen based on preliminary growth curves, selecting concentrations at least 10-fold lower than the carrying capacity of the medium to avoid stationary phase and pH drift due to carbon-concentrating mechanisms or metabolite accumulation (Fig. S22). We targeted 108 transfers for each lineage, representing log_2_26 or 4.7 generations (although we did not achieve this goal for some lineages due to repeated crashes or contamination, Table S1). Samples from each lineage were cryopreserved every 25 generations and again at the end of the experiment.

We monitored both the media, media additions, and cultures for external contamination throughout the experiment. Each time transfers were performed, 0.5 ml of any media batch used were added to 5 ml of YTSS broth and monitored for at least 7 days for evidence of growth. *Synechocystis* cultures, which were grown axenically, were directly tested for bacterial contamination by transferring 0.5 ml into YTSS broth after transfer. For cultures containing *Alteromonas*, we periodically spread-plated cultures on YTSS media and examined colony morphology for evidence of bacteria other than *Alteromonas*, which forms distinctive large, shiny brown colonies.

After transfer into fresh media, the previous generation’s tube from a given replicate evolutionary lineage was placed back in the same incubator under low (<30 μmol photons m^−2^ s^−1^) light conditions to slow growth. At least three previous transfers were stored in this manner. In rare instances (see Table S1), when a culture failed to grow after transfer or contamination was detected in a culture or in the media, we restarted the line from the last transfer tube to have viable cells and no evidence of contamination. In a small number of cases, we had to revive a culture from the last cryopreserved sample due to slow-growing contaminants.

**Fig. 1:**
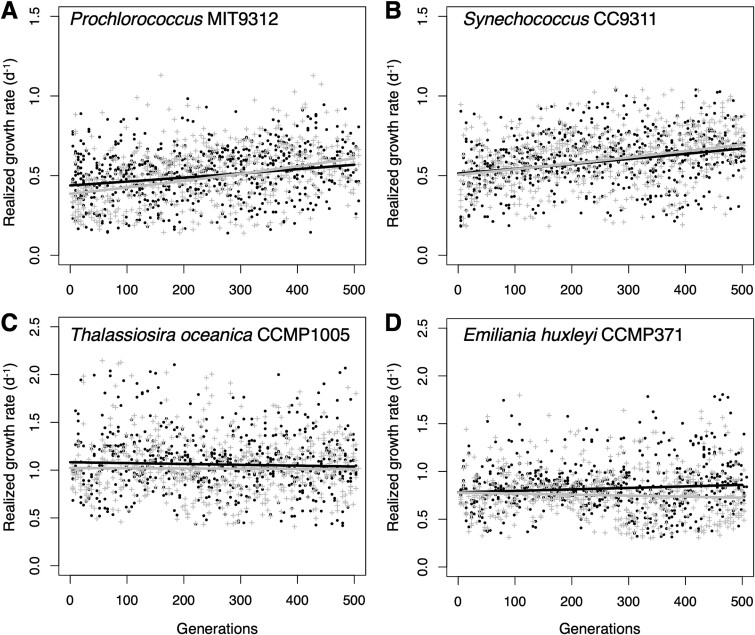
Evolution of the realized growth rates of phytoplankton at 400 and 800 ppm pCO_2_. Each point represents the realized growth rate of one transfer (log_2_ 26 = 4.7 generations) of the (A) *Prochlorococcus*, (B) *Synechococcus*, (C) *T. oceanica*, or (D) *E. huxleyi*. Trendlines are regressions of growth rate on generation. All 12 replicate lineages are plotted together for each panel. Black points and lines = 400 ppm cultures, gray crosses, and lines = 800 ppm cultures.

We only measured cell density in evolving cultures every 48 h because of the relatively slow growth rates (especially of the cyanobacteria) and because of the amount of time needed to sample and read 58 cultures. Also, we inferred the initial cell density of a culture posttransfer by dividing the final cell density of the previous transfer by the dilution factor of 26. Because of these factors, the calculated realized growth rates (RGRs) depicted in Fig. 1 are conspicuously noisy, particularly for the faster-growing eukaryotes. For this reason, we remeasured growth rates with higher temporal resolution and greater (5×) within-lineage replication using the final populations at the end of the evolutionary period. These final growth rates were used for all subsequent analyses.

### Synechocystis *cultures*

We began this project by testing the protocols and procedures on cultures of *Synechocystis* PCC6803, a model cyanobacterium that is significantly easier to cultivate than *Prochlorococcus* or *Synechococcus*. Because these cultures were grown axenically, and because *Synechocystis* is not an open-ocean phytoplankton, we chose not to report these data alongside that of the four primary species studied. Data from the analysis of *Synechocystis* growth rates and genomes are depicted in Figs S24, S25, S26, S27, and S28, with detailed descriptions in the figure legends. Individual *Synechocystis* mutations are included in Data S1 alongside those of other phytoplankton studied.

### Mix-and-match growth experiments

At the end of the evolution period, we subcultured clonal evolved *Alteromonas* strains by spread-plating evolved cultures on YTSS agar and selecting single, isolated colonies for growth in YTSS broth. *Prochlorococcus* was rendered axenic by the addition of 100 μg ml^−1^ streptomycin. All growth experiments were initiated by mixing axenic *Prochlorococcus* with a specific *Alteromonas* clone (or else remaining axenic) and acclimating the coculture for three transfer cycles (~14 generations) at the target pCO_2_ concentration. Growth was then monitored by flow cytometry as described above for at least three subsequent transfers under constant conditions. Cultures were considered to have died if they did not show signs of sustained exponential growth after 4 weeks from inoculation. We calculated realized and exponential growth rates (EGRs) as described in [7]. Both evolved clones as well as revived cryopreserved ancestral *Prochlorococcus* and *Alteromonas* were examined.

### Statistical analysis of growth data

The impact of experimental treatments on growth parameters was statistically analyzed using linear models in R with *post hoc* statistical testing using extended marginal means with the *emmeans* package [46]. Linear model residuals were checked graphically for deviations from normality; where these were discovered, we used the Box-Cox process [47] to find the optimal power transformation of the data in order to produce an improved final model from which we reported and graphed results.

Realized and EGRs were calculated as described in [7]. Because these experiments involved thousands of measurements collected over several years by different researchers, a variety of clearly erroneous data points were recorded that led to several outlier growth rates that had a disproportionate impact on model output; rather than attempt to manually curate all growth rates, we simply removed either the most extreme 5% high and low RGRs or eliminated EGRs with *R*^2^ values lower than 0.95 for each strain in each experiment before conducting statistical tests.

Because growth rates evolved differently in different lineages, contrasts involving evolved strains were performed using both linear models and linear mixed effects models with the *lme4* package in R [48] with lineage as the random contrast; in all cases, the plain linear model yielded a better fit, assessed by lower Bayesian Information Criterion.

We documented many instances of *Prochlorococcus* cultures failing to grow, especially when they were grown axenically. We assessed the impact of pCO_2_ and *Alteromonas* on culture viability using binomial logistical regression with each attempted culture coded as either “Alive” or “Dead”.

### Whole-genome resequencing

Postevolution cultures were split into five replicate 13 ml culture tubes, grown to the cutoff transfer cell density, and then collected by gentle vacuum filtration on 0.2 μm pore size polycarbonate filters, then flash frozen in liquid nitrogen and stored at −80°C. Genomic DNA was extracted from the filters using MoBio ProSoil kits, with the bead-beating step accomplished using a MP FastPrep-24 homogenizer. DNA was fragmented, ligated with Illumina adapters, and sequenced on an Illumina NextSeq500 device.

FastQ files (NCBI BioSamples SAMN34542194 through SAMN34542251) were analyzed using breseq [49] in polymorphism mode with default settings except as mentioned below. *Synechocystis* PCC6803 cultures were assembled against the chromosomal reference genome (NCBI accession number NC_000911) as well as its four plasmids (NC_005229, NC_005230, NC_005231, and NC_005232). *Prochlorococcus* MIT9312 and *Synechococcus* CC9311 were assembled against their RefSeq genomes (respectively, CP000111 and NC_008319). The *T. oceanica* CCMP1005 and *E. huxleyi* CCMP371 reference genomes were still in draft form and were assembled in breseq with the contig flag activated; all contigs were downloaded as a single GenBank format file from NCBI BioProject PRJNA36595 (CCMP1005) or BioSample SRX112492 (CCMP371, also known as strain 12–1). The *T. oceanica* CCMP1005 sequence file contained the *T. oceanica* chloroplast sequence but *E. huxleyi* CCMP371 did not; we therefore used the chloroplast sequence of another *E. huxleyi* strain, CCMP373, also with downstream curation, to analyze mutational changes to those sequences. For both *T. oceanica* and *E. huxleyi*, breseq was set to not attempt to predict structural variants due to the much greater computational demand required to assemble these large genomes relative to the bacteria.

*Alteromonas* genomes were assembled against the most recent EZ55 genome [50], including both the chromosomal (CABDXN010000001) and plasmid (CABDXN010000002) sequences in the same GenBank format file. For *Prochlorococcus* and *Synechococcus* lineages, EZ55 genomes were assembled in the same breseq run as the phytoplankton genome. However, because of the computational demand of assembling *T. oceanica* and *E. huxleyi* genomes, the *Alteromonas* portion of these cultures was assembled separately to allow for structural variant prediction.

Any specific mutational call present in 100% of replicate lineages for a given organism was considered to have occurred prior to the initiation of the experiment (i.e. it was ancestral) and was removed from further consideration. Because of additional genomic complexity in the eukaryotic genomes of *T. oceanica* and *E. huxleyi*, we undertook additional efforts to curate these datasets. First, coverage for the eukaryote genomes was lower than for the bacterial genomes, and some loci had insufficient coverage to make predictions about mutations. All these loci were removed from breseq output files prior to further analysis. Also, because the GenBank files we used as reference sequences represented haploid sequences, we sought to discover potentially heterozygous loci in the ancestral genomes. To do this, we reassembled the raw read files from the original genomic sequencing runs for both organisms against the GenBank reference sequences using breseq (SRX112492 for *E. huxleyi* and all sequencing runs within BioProject PRJNA36595 for *T. oceanica*). We identified 4479 and 79 340 variants present in 100% of *T. oceanica* and *E. huxleyi* reads, respectively, compared to the published GenBank sequences, perhaps suggesting differences between breseq’s handling of data and the programs used to produce the published sequences; all mutations assigned by breseq to these loci were therefore removed from consideration. We also discovered 96 556 and 132 276 variants present in 0% < *n* < 100% of sequences in the *T. oceanica* and *E. huxleyi* reference genome reassemblies, respectively. These were assumed to represent loci that were heterozygous in the ancestral population. Mutations mapped to these loci were only considered further if they became fixed in evolved lineages; if they remained at intermediate frequencies, they were removed from subsequent analyses. In most cases, these curation steps led to a modest (~10%) reduction in the number of mutations identified by breseq that were considered in our downstream analysis (Fig. S29A). Tables S3–S5 show the number of identified mutations surviving each curation step.

Mutational frequency analysis indeed suggested (Fig. S7) that *T. oceanica* and *E. huxleyi* retained substantial levels of heterozygosity during the experiment and were possibly undergoing sexual reproduction (although we did not attempt to directly observe sexual stages or ploidy changes during the experiment). Overall, around 30% of ancestrally heterozygous loci fixed in most lineages, although three *E. huxleyi* lineages showed much more pronounced loss of heterozygosity of 65–90% (Fig. S29B). It is likely that these curation steps removed many true mutations from the dataset; however here, as with downstream analyses described below, we opted for a highly conservative methodology for choosing putative mutations for analysis.

### Mutational analysis

We used the application gdtools from the breseq package to convert breseq output genome difference files into long-format data files for subsequent analysis. Custom python scripts were used to bin all mutations within a given gene in each lineage, producing mutational count tables of genes vs. lineages. For bacterial genomes, we produced count tables either with or without considering insertions, deletions, and intergenic mutations within 50 bp upstream of a gene’s start codon (i.e. putative cis-acting promoter or other regulatory elements) in addition to mutations within a gene’s coding region. Eukaryotic count tables only considered coding sequences; all intergenic and intron regions were removed from analysis. We used python scripts to analyze three metrics of directional selection: dN/dS [51], transition:transversion ratio [52], and nonsense mutation ratio [53]. All three metrics exclusively used SNP data from coding regions of annotated genes. Effects of treatment groups on these metrics were analyzed using linear models in R.

We sought to test for convergent evolution of gene targets by determining which, if any, genes received more mutations than would be expected by chance under a completely random mutational model. This analysis was complicated by the fact that larger genes represented larger mutational targets than smaller ones, precluding the use of a simple Poisson model. Instead, we used a bootstrapped Monte Carlo procedure to produce randomized genomes with the same number of coding sequence mutations observed in our real dataset, only distributed randomly across the genome. Given the total coding genome size *g* as the sum of the lengths of all coding sequences in the genome, and the total number of observed mutations *n*, the probability *p* of any given base pair receiving a mutation is *p* = *n*/*g*, and the probability *λ* of a gene of length *l* receiving a mutation is thus *λ* = *p* x *l*. For each lineage, we produced 100 randomized matrices where each gene in the genome was assigned a number of mutations drawn from a Poisson distribution with average rate of occurrence *λ*. Each random matrix was compared to the real matrix of mutations by first converting each into an empirical cumulative distribution function (ECDF) and then applying either a Kolmogorov–Smirnov (KS) or dts test [54]. The KS test only considers the single value in the ECDF where the gap between the two samples is greatest, whereas the dts test considers the entire distribution. Because the greatest difference between our observed and Monte Carlo matrices was the fact that the real dataset had many more genes with large numbers of mutations than were ever observed in any simulation (Table S6), the dts test was generally more sensitive in detecting significant differences. These procedures were conducted separately for nonsynonymous and synonymous mutations and revealed that the distribution of both kind of mutations was significantly different from random expectations (dts test, *P* < 0.01; KS test, *P* < .001 for all comparisons except for *Prochlorococcus* synonymous mutations which were not significantly different from random).

Because genomes are known to include mutational hot spots [55] and because both nonsynonymous and synonymous mutations were more common than expected by chance, we performed additional curation of multiply mutated genes to find genes that were most likely targets of natural selection instead of accelerated mutational rates. We only considered genes as convergently evolved if they (i) accumulated more mutations across replicate evolved lineages than *any* gene received in any randomized bootstrap trial or (ii) were observed in at least half of all replicate evolved lineages. Additionally, reasoning that mutational hot spots should not be biased for or against silent mutations, the gene had to meet these criteria for the nonsynonymous mutation dataset but not also the synonymous mutation dataset. We applied these tests separately for each treatment group (e.g. pCO_2_ treatments for phytoplankton genomes or pCO_2_ × partner for *Alteromonas* genomes). We further supported this curation step by performing a gene-by-gene linear model test in R to discover genes significantly more mutated under one treatment than another, accepting an unadjusted *P* value <.05 as evidence of an effect of the treatment on the likelihood of detecting a mutation in that gene.

Genes remaining after this curation process were then analyzed for function. First, we binned genes into KEGG pathway groups using over representation analysis (ORA) with the function *enrichKEGG* in *clusterProfiler* in R [56]. The argument “*organism*” was set to the KEGG organism codes “*pmi*” and “*syn*” for *Prochlorococcus* and *Synechocystis*, respectively, whereas it was to set to “ko” (KEGG Orthology) for *Synechococcus*, *T. oceanica*, *E. huxleyi*, and *Alteromonas*. KO identifiers to individual genes were assigned for each organism using the KEGG automatic annotation server website by the bidirectional best hit (BBH) method [57]. *P* values correspond to the comparison between the gene ratio (i.e. the number of genes that match that gene set divided by the number of genes in the “hit” database) and the background ratio (i.e. the number of genes in the gene set divided by the total number of unique genes in the genome database). Under specific contrast comparisons for *Alteromonas* considering the mixed effect of cocultures (i.e. MIT9312:CC9311, MIT9312:CCMP1005, MIT9312:CCM371, MIT9312: CCMP1005:CCM371 and all phytoplankton partners), KO identifiers were examined directly using the KEGG database website [58].

The degree of association between mutating genes in EZ55 was analyzed using the Species Pairwise Association Analysis (SPAA) algorithm in R [59], reasoning that presence versus absence of mutations in a given gene in a culture was mathematically comparable to presence versus absence of species in an ecosystem. The mutation data was transformed into a binary presence versus absence matrix, with genes having at least 1 mutation in each lineage coded as 1 and those without mutations coded as 0. The logistic regression function was used to calculate the odds ratio for mutations in each gene as described in [59], using cutoffs of 0.5 and 0.9 for the minimum and maximum mutation frequencies, respectively. SPAA estimated Spearman’s rank correlation coefficient for each gene pair, measuring the monotonic predictive relationship, i.e. the likelihood of a mutation in one gene predicting a mutation in the other. After manually examining the data, we further filtered the pairwise predictions to consider only the most positively and negatively correlated pairs (coefficients between 0.5 and 0.9 for positive relationships and −0.3 to −0.2 for negative relationships). The curated matrix was imported in Cytoscape 3.8.2 for network visualization [60]. The network was analyzed as a directed graph to obtain the indegree and outdegree of each node, i.e. the number of other genes to which each gene is connected as a predictive target or source, respectively. The target gene of each set of genes was illustrated as an arrow pointed toward the target gene, and the gradient of the edge connecting genes was set to reflect either a positive or negative correlation.

We manually examined several genes of interest identified from these analyses using BLAST [61] against the NCBI nr database to attempt to provide superior annotations for hypothetical proteins or other vaguely described gene products.

### Copy number analysis

We used R scripts to extract coverage data from all breseq-assembled genomes (summarized in Table S2). Estimated copy numbers for plasmids reported in Table S2 were initially obtained simply by dividing average plasmid coverage by average chromosome coverage, and these values were sufficient for understanding the plasmid copy numbers in *Synechocystis* genomes (Fig. S28). However, inspection of coverage maps for the *Alteromonas* plasmid revealed highly uneven coverage (Fig. S6). Closer analysis revealed three plasmid regions with different patterns of coverage. One region was generally present at the same or greater coverage as the chromosome, one was often completely absent, and a third often existed at an intermediate coverage level. We reasoned that these differences may reflect plasmid loss, homologous recombination events leading to the excision of parts of the plasmid, and/or insertion of plasmid regions into the *Alteromonas* chromosome and therefore looked for homologous regions between the plasmid and chromosome sequences using a dot plot analysis via the D-GENIES web interface [62]. This analysis revealed several points of homology, including one ~9 kb sequence (Fig. S30), thus suggesting two things. First, a reduced-size plasmid likely evolved as a subpopulation in several lineages, with ~50 kb deleted following an unknown but reproducible event. Second, an ~70 kb section likely integrated into the chromosome of most lineages, and in several lineages became the only surviving portion of the plasmid, with no evidence remaining of subpopulations carrying the remaining 150 kb of the plasmid. Because of these trends, we calculated average coverage of the plasmid at three different regions: 170–180 kb to estimate the “insertable” portion of the plasmid, 120–130 kb to estimate the full, free plasmids, and 40–50 kb to represent the possible reduced-size plasmid. We tested the effects of phytoplankton partner and pCO_2_ treatment on *Alteromonas* plasmid copy number using pairwise Mann–Whitney tests in R with manually calculated Holm–Bonferroni corrections for multiple tests [47]. We also compared the likelihood of plasmid loss (defined as less than 1% coverage of plasmid region 120–130 kb) in *Alteromonas* paired with cyanobacteria vs. eukaryotic phytoplankton using Fisher’s exact test in R.

Examination of coverage maps further revealed the presence of elevated copy numbers for some chromosomal regions, suggesting the presence of duplications. We therefore investigated these more closely using R scripts, retrieving any regions where the average coverage was 5× the coverage (or 5× the standard deviation of coverage for low-coverage eukaryote genomes) across the chromosome. We excluded from further analysis all duplications in eukaryotic genomes where repetitive elements led to tens of thousands of qualifying duplications, as well as duplications falling outside of coding sequences or cis-acting promoter regions. After this process, only one duplication remained of interest: a promoter region mutation in the apolipoprotein N-acyltransferase gene in *Prochlorococcus* MIT9312, clearly visible in coverage maps (Fig. S1) and present in most lineages with up to 120× duplication.

## Results

### Evolution of phytoplankton growth characteristics

**Fig. 2:**
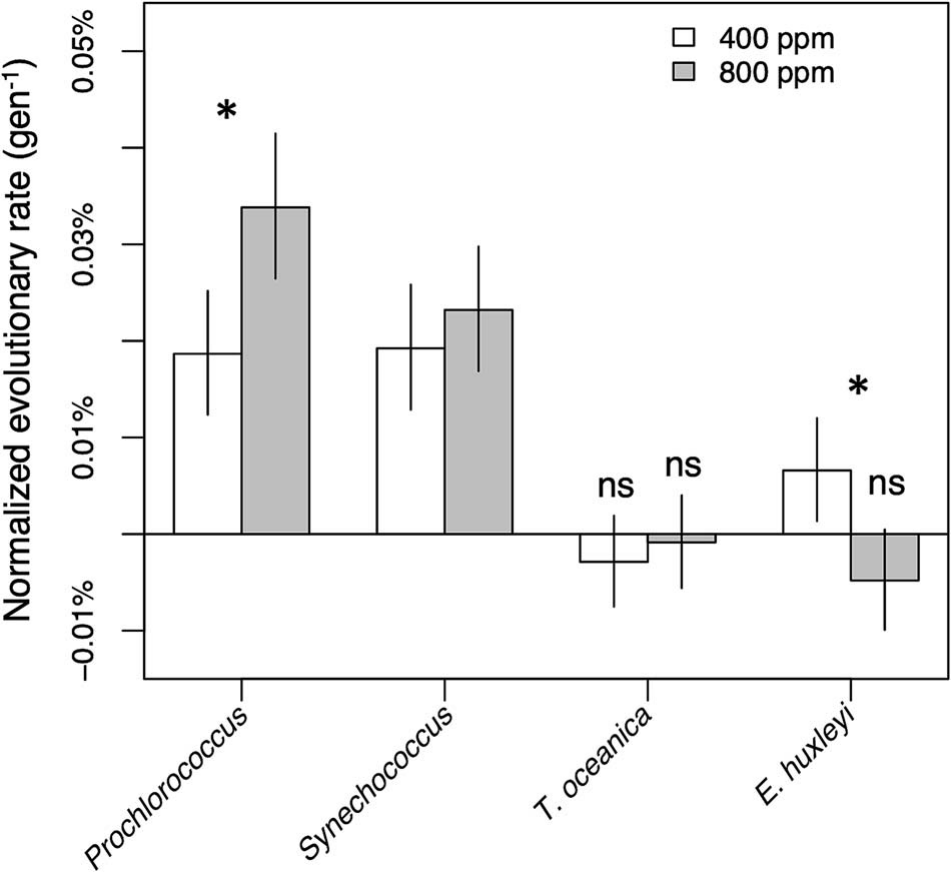
Growth rate change over the course of evolution. Normalized evolutionary rates were expressed as the slope of growth rate change (i.e. regression lines in Fig. 1) divided by the estimated ancestral growth rate (i.e. the y-intercept of the regression). Error bars are 95% confidence intervals of the estimates. Asterisks represent *P* < .05 in *post hoc* tests of the evolutionary rates of 400 versus 800 ppm pCO_2_ cultures. All evolutionary rates were significantly greater than 0 except for those marked “ns” or not significant.

We tracked phytoplankton growth in six replicate cultures of each phytoplankton at each pCO_2_ condition, achieving at least 500 generations for most cultures (Table S1). Although there was substantial variability transfer-to-transfer in RGRs, regression lines fit to the overall data for each of the 48 lineages revealed clear fitness trends (Fig. 1), with all cyanobacterial lineages and one eukaryotic phytoplankton treatment (*E. huxleyi*, 400 ppm pCO_2_) exhibiting significant increases in growth rate over the course of the experiment (Fig. 2). The pace of growth rate change was only significantly different between pCO_2_ conditions for two species; however, *Prochlorococcus’* growth rate evolved faster at 800 ppm pCO_2_, and *E. huxleyi*’s evolved faster at 400 ppm (Fig. 2).

**Fig. 3:**
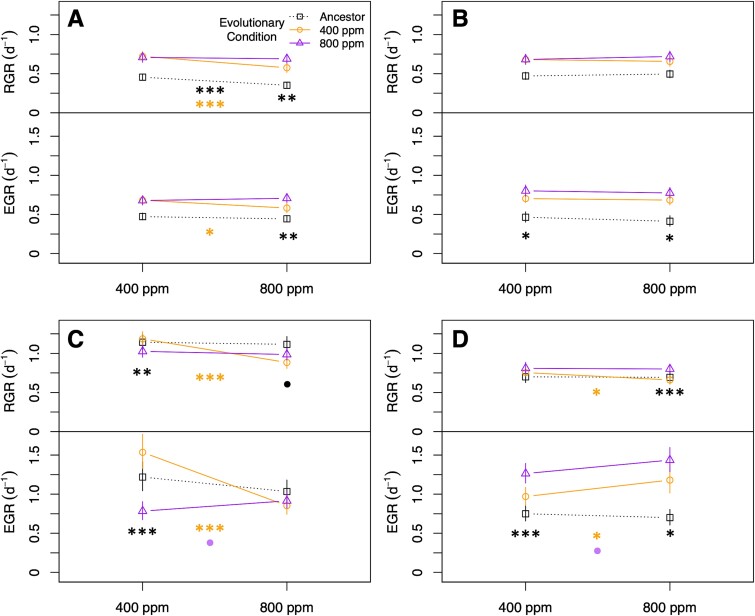
Pre- and postevolution reaction norms for four phytoplankton species. (A) *Prochlorococcus*, (B) *Synechococcus*, (C) *T. oceanica*, and (D) *E. huxleyi*. RGR, realized growth rate; EGR, exponential growth rate. The legend in panel (A) applies to all panels. Error bars represent 95% confidence intervals for the growth rate estimate at the indicated assay pCO_2_ condition. Asterisks in the center of plots indicate significant differences between pCO_2_ treatments; asterisks at ends of reaction norms indicate significant differences between the responses of the evolutionary treatments at the assay pCO_2_. Colors of asterisks correspond to colors of lines. ^*^P < .05; ^*^^*^P < .01; ^*^^*^^*^P < .001.

At the end of the experiment, we investigated whether evolution at 400 or 800 ppm pCO_2_ had produced a correlated response [63] in RGR and/or EGR in the opposite treatment condition for any of the phytoplankton species (Fig. 3), potentially indicating adaptive specialization to the changed environment. Where the EGR reflects the raw growth potential of the organisms, the RGR also includes the lag or die-off after culture transfer, possibly reflecting stress response [7]. At the beginning of the evolution experiment, *Prochlorococcus* had a significantly slower RGR at 800 ppm; however, when the 800 ppm-evolved lineages were grown under both pCO2 conditions, there was no longer a significant difference in RGR between the pCO_2_ treatments (Fig. 3A), indicating that the negative response to elevated pCO_2_ was eliminated as an adaptive response to growth at 800 ppm. In contrast, 400 ppm-evolved *Prochlorococcus* retained significantly reduced RGR and EGR at 800 ppm compared both to itself at 400 ppm and to the 800 ppm cultures grown at 800 ppm (Fig. 3A). 800 ppm-evolved *Synechococcus* lineages grew faster than 400 ppm-evolved lineages under both pCO_2_ assay conditions, but (as with their ancestor) pCO_2_ treatment had no effect on either their EGRs or RGRs (Fig. 3B). For *T. oceanica*, both evolved lineages had greater RGRs and/or EGRs in their evolved milieu than in the opposite pCO_2_ condition (Fig. 3C). *T. oceanica* was also the only strain to show evidence of adaptive tradeoffs, with both evolved strains growing more slowly (by EGR and/or RGR) than their ancestor under the pCO_2_ condition opposite from their evolutionary condition (Fig. 3C). In contrast, 800 ppm-evolved *E. huxleyi* lineages grew faster than 400 ppm-evolved lineages under both pCO_2_ assay conditions (Fig. 3D). EGR increased when assayed at 800 ppm for both evolutionary treatments, but 400 ppm-evolved lineages had slower RGRs at 800 ppm than 800 ppm lineages, similar to the response of ancestral and 400 ppm-evolved *Prochlorococcus*.

### Genomic evolution and evidence of adaptation

To better understand the genetic mechanisms behind the evolution of growth rates and pCO_2_ responses in these organisms, we obtained shotgun metagenomic sequences (>50× coverage for prokaryotes, >15× coverage eukaryotes, Table S2, Figs S1, S2, S3, S4, S5, and S6) for each evolved population and predicted single nucleotide polymorphism (SNP) and simple insertion/deletion (indel) mutations in each of the genomes compared to the reference ancestral genome. We observed thousands of mutations existing at various abundances above our 5% per population cutoff (Table S3), with strikingly different trends for the different species. The cyanobacterial taxa had several dozen fixed mutations observed across the replicate lineages, with large numbers of rarer mutations (Fig. S7A-B). Similar patterns were observed for *Alteromonas*, albeit with a much greater number of rare mutations relative to fixed ones (Fig. S8). In contrast, *T. oceanica* and *E. huxleyi* genomes showed very large numbers of fixed mutations, and whereas the distribution of cyanobacterial nonfixed mutations was left-skewed toward rarer mutations, the eukaryotes showed a roughly normal distribution of mutation frequencies, centered on 50% (Fig. S7C, D). We believe these differences between bacteria and eukaryotes reflect differences in how natural selection affects sexual and asexual genomes. Because of their obligate asexuality under our experimental conditions, fixation of a new mutation in a bacterial genome requires a selective sweep, such that each fixed mutation purges all previously existing diversity, generating a bimodal distribution weighted heavily toward rarer, more recent mutations. The eukaryotes, in contrast, may have engaged in sexual reproduction at least some of the time, allowing beneficial mutations to fix without a loss of diversity, and at a much greater rate due to an absence of clonal interference [64]. An accumulation of heterozygotic strains may account for the normal distribution of novel mutations in eukaryotic populations. However, these organisms have complex life cycles, and we did not make any attempts to observe or quantify sexual reproduction during the experiment.

**Fig. 4:**
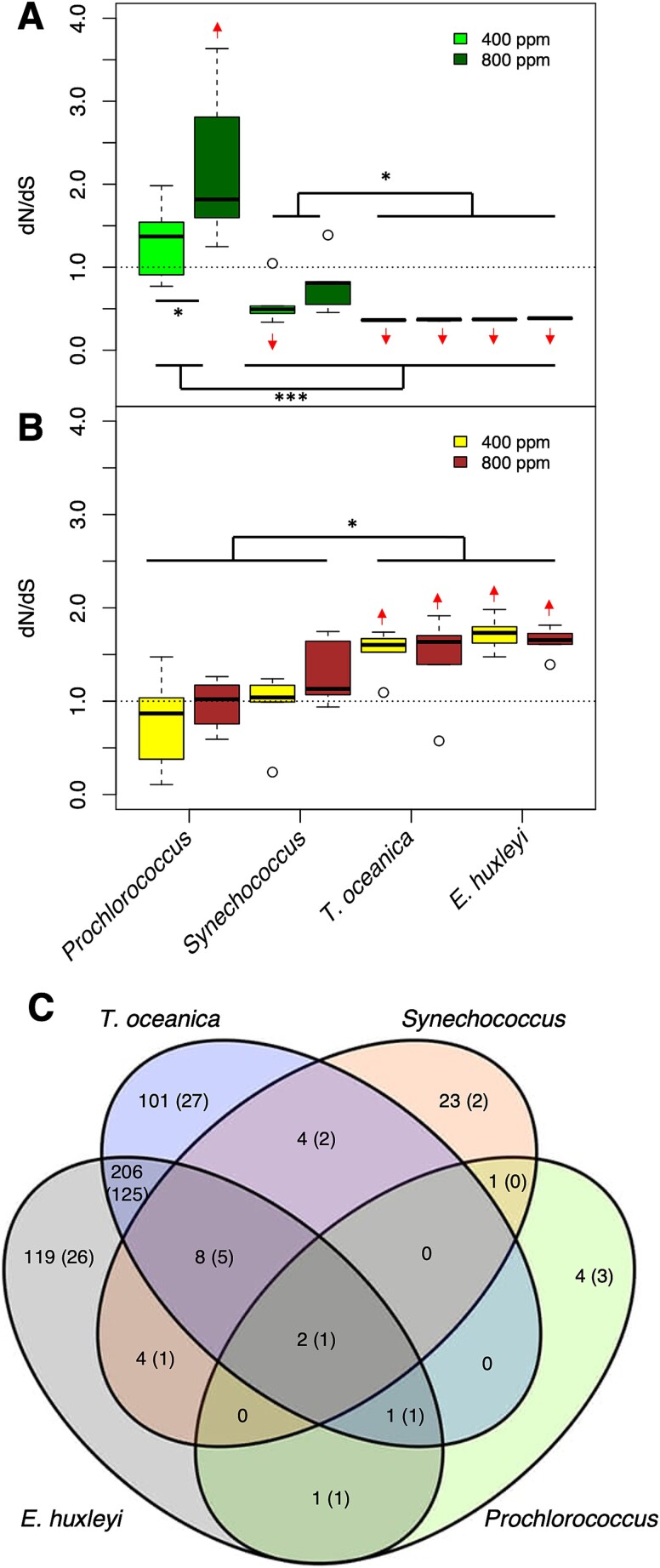
Genomic evidence of adaptive evolution. Panels show the ratio of nonsynonymous to synonymous (dN/dS) amino acid changes in coding sequences of phytoplankton species (A) or *Alteromonas* strains paired with the indicated phytoplankton species (B). Dashed lines indicate the expected value under neutral evolution; arrows indicate predicted means significantly higher or lower than this expected value (linear model, 95% confidence interval of the extended marginal mean). Asterisks indicate significant differences between pCO_2_ treatments within a species, or between species or groups of species, based on a linear model (*P* < .05). (C) The Venn diagrams show counts of genes in *Alteromonas* genomes that were significantly more mutated by phytoplankton partner. In order to be considered significantly multiply mutated, a gene had to have either (i) more observed nonsynonymous, indel, or promoter mutations than in any of our bootstrapped dummy datasets (see methods), and to not also have more synonymous mutations than in the dummy datasets, or (ii) it had to have at least one observed nonsynonymous mutation in at least 50% of replicately evolved lineages. Values indicate the number of genes passing these criteria for the partners indicated by overlapping ovals; values in parentheses indicate the number of thus-identified genes that were also marked as statistically significantly differentially mutated between at least one pair of partners in a linear model.

The spectrum of mutational types also suggests that natural selection rather than random drift or sequencing errors was responsible for the detected variants. The relative abundance of nonsynonymous and nonsense mutations, both of which result in altered proteins upon translation and are therefore more likely to result in phenotype changes than synonymous or intergenic mutations, varied greatly among phytoplankton taxa (Fig. S9) and among *Alteromonas* populations based on which phytoplankter they evolved alongside (Fig. S10). The ratio of nonsynonymous to synonymous substitutions (dN/dS ratio) for *Prochlorococcus* evolved at 800 ppm pCO_2_ was significantly greater than 1 (Fig. 4A), suggesting that directional natural selection drove the evolutionary process. dN/dS was also significantly higher for both cyanobacteria than for the eukaryotes, but in most cases non-*Prochlorococcus* phytoplankton had dN/dS ratios significantly less than 1, suggesting that purifying selection, not drift, dominated evolution in these cases (Fig. 4A). Similar trends were observed for the abundance of nonsense mutations, which were very rare for eukaryotes under both pCO_2_ conditions but were significantly elevated for *Prochlorococcus* evolved at 800 ppm pCO_2_ (Fig. S11A). Similarly, the transition:transversion ratio (where lower values generally correspond to greater likelihood of phenotypic change [65]) was much higher than expected under neutral conditions for both eukaryotes and was significantly higher for eukaryotes than cyanobacteria but was significantly reduced for *Prochlorococcus* at 800 ppm pCO_2_ (Fig. S11B). In short, the spectrum of mutations in evolved *Prochlorococcus* was suggestive of changes to protein function during adaptation to future pCO_2_, whereas eukaryotic phytoplankton (and, to a lesser degree, *Synechococcus* and 400 ppm pCO_2_-evolved *Prochlorococcus*) accumulated mutations that were less likely to alter or disrupt protein function, with no difference between pCO_2_ treatments.

In most cases, these trends were reversed for *Alteromonas*: when the phytoplankton partner exhibited evidence of purifying selection and conservation, *Alteromonas* showed signs of directional change, and vice versa (Figs 4B, S11C–D). *Alteromonas* EZ55 was originally isolated from a *Prochlorococcus* culture from the same HLII ecotype as the strain used in this study [37], so it is not surprising that all three mutational type metrics (i.e. dN/dS ratio, abundance of nonsense mutations, and transition:transversion ratio) support conservation instead of change with *Prochlorococcus* under current pCO_2_ conditions. In general, all these metrics moved in favor of directional evolution as the partner became more evolutionarily distant from *Prochlorococcus*. In all cases, these metrics were significantly greater than the value expected by chance for *Alteromonas* evolved alongside eukaryotic phytoplankton and were significantly different between eukaryotic and cyanobacterial partners. In the great majority of case, none of the metrics of adaptive evolution in *Alteromonas* lineages were significantly affected by the evolutionary pCO_2_ treatment. We thus conclude that the primary driver of adaptation for *Alteromonas* was adaptation to its coculture partner.

### Mutational targets for convergent evolution

To detect specific mutational targets, we searched for genes with more observed mutations than expected by chance. First, we assessed whether overall mutational distribution patterns deviated from random expectations using a Monte Carlo bootstrapping process. In all cases, the maximum number of mutations in a single gene observed in the actual dataset was much higher than any bootstrapped dataset (Table S3). However, empirical cumulative distribution of actual and dummy per-gene mutational frequency datasets did not consistently differ statistically (KS and DTS tests, see Methods), with a few highly mutated loci conspicuously driving the difference between the two. Furthermore, nonrandom mutational distributions were observed for both nonsynonymous and synonymous mutations, possibly suggesting the presence of mutational hotspots obscuring potentially convergently evolved beneficial mutations [55]. We therefore curated a list of individual genes that were most likely to be adaptive evolutionary targets by selecting only nonsynonymous mutations (including mutations within promoter regions up to 50 bp upstream of a prokaryotic start codon) that either (i) received more mutations than were observed in any bootstrap dataset or (ii) had mutations in at least half of eligible replicate lineages. We further removed any genes that also fit either of these criteria for synonymous mutations. We applied these selection criteria separately for lineages evolved under each pCO_2_ regime, so a given gene could appear in either treatment or in both. We also applied linear models to test for differences in mutation frequencies between pCO_2_ treatments for genes passing these criteria, with uncorrected *P* values <0.05 considered supportive of a treatment effect.

Using these conservative criteria, relatively few mutations remained for cyanobacteria (Data S1, Fig. S12), although several pathways were nevertheless statistically over-represented (Fig. S13). Every *Prochlorococcus* lineage under both pCO_2_ treatments contained one of several promoter region mutations upstream of the gene for plastoquinol terminal oxidase (PTOX), and most also had similar mutations upstream of thioredoxin reductase; both of these proteins are involved in protection against oxidative stress in cyanobacteria [66, 67] and could underlie the increased resilience of evolved axenic MIT9312 cultures (see below). Most *Prochlorococcus* populations also had expansions of AT repeats (Fig. S1) in the promoter/5′ region of the apolipoprotein N-acyltransferase gene that encodes an integral membrane protein involved in outer membrane lipoprotein maturation in *E. coli* [68], with significantly more polymorphisms observed in 800 ppm pCO_2_-evolved cultures. Lipoproteins are involved in many intercell interactions in bacterial pathogens [69] and may affect how *Prochlorococcus* and *Alteromonas* interact with each other and possibly also with secreted membrane vesicles in their environment [11], but it is unclear how such changes affect growth at elevated pCO_2_. In contrast, no single gene was mutated in more than half of the *Synechococcus* cultures, and only a single ribosomal protein was differentially mutated between pCO_2_ treatments.

Far more mutations passed the filter for eukaryotic phytoplankton (Data S1, Fig. S12), with genes involved in biosynthetic pathways, carbon metabolism, and peroxisome functions strongly overrepresented (Fig. S14). The diatom *T. oceanica* had dozens of pathways statistically overrepresented, mostly in populations evolved at 400 ppm. In contrast, *E. huxleyi* had more pathways overrepresented in lineages evolved at 800 ppm pCO_2_, including several pathways related to lipid metabolism. Most genes that passed our cutoff for *T. oceanica* only did so in one or the other pCO_2_ treatment, whereas most *E. huxleyi* multiply mutated genes passed the filter under both treatments (Fig. S12). Even though it is unclear what drove this difference, it is noteworthy that *T. oceanica* was the only phytoplankton species that showed evidence of a growth rate tradeoff when adapting to the different pCO_2_ regimes (Fig. 3), possibly suggesting a more specific adaptive response to pCO_2_ for this particular organism.

The mutational targets for *Alteromonas* differed between populations evolved with different phytoplankton partners (Data S2, Fig. S15). Relatively few mutations and pathways passed our filters in strains paired with cyanobacteria, consistent with our observation of mostly purifying selection under these conditions for *Alteromonas* except when paired with *Prochlorococcus* under 800 ppm pCO_2_, possibly reflecting *Alteromonas’* long history of growing in coculture with *Prochlorococcus* under ambient pCO_2_ conditions. When evolved alongside eukaryotes, however, many pathways were found to be significantly over-represented in the mutational data (Fig. S16), and conspicuously more genes were observed to be multiply mutated under 800 ppm than under 400 ppm pCO_2_ (Fig. S15). Starch and sucrose catabolic pathways were overrepresented in *Prochlorococcus* partners as well as *Synechococcus* at 800 ppm pCO_2_ but not with either eukaryotic phytoplankton. Genes involved in the synthesis and catabolism of amino acids were multiply mutated with all partners, but the particular pathways were distinct: for instance, *Alteromonas* evolved alongside *Prochlorococcus* at 800 ppm pCO_2_ were more likely to have mutations in tryptophan and aspartate metabolism, whereas those at 400 ppm pCO_2_ were more likely to have arginine pathways mutated, and *Synechococcus*-paired lineages favored proline mutations (Fig. S16). Also, in contrast to the mutational pattern for the phytoplankton themselves, *Alteromonas* lineages paired with *T. oceanica* had many more pathways over-mutated at 800 than at 400 ppm pCO_2_. Regardless of the phytoplankton partner or pCO_2_ treatment, two-component regulatory systems and genes related to chemotaxis, flagellar synthesis, and biofilm formation were convergently mutated across *Alteromonas* lineages, consistent with these genes previously being shown to be differentially regulated in ancestral *Alteromonas* based on partner and pCO_2_ conditions [6]. The most mutated EZ55 gene was an unannotated putative lipoprotein (EZ55_02425) observed in 47 out of 48 *Alteromonas* lineages (Data S2), with almost all mutations falling within the 50-bp promoter region we considered in our analysis, suggesting possible cell-surface alterations like those we speculated above may have occurred in *Prochlorococcus*.

**Fig. 5:**
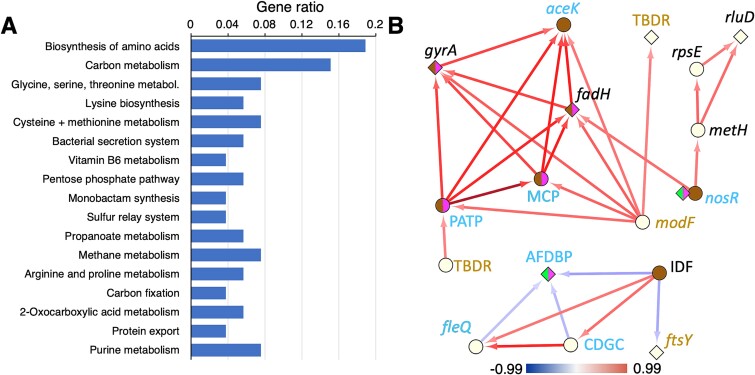
Metabolic evolution in *Alteromonas* EZ55. (A) Pathways analysis of *Alteromonas* EZ55 genes convergently mutated in coculture with eukaryotic phytoplankton. Genes passing our multiple mutation screening criteria only for both *T. oceanica* CCMP1005 and *E. huxleyi* CCMP371 were assigned to KEGG pathways as described in the methods. Only pathways with adjusted *P* < .05 for the statistical test of overrepresentation are depicted. (B) Network analysis of multiply mutated genes in Alteromonas. Nodes indicate genes and arrows indicate that mutations in one gene significantly predict mutations in the second gene (at the end of the arrow) in the same evolved EZ55 culture. Diamond nodes indicate genes where only nonsynonymous or promoter region mutations were observed; circular nodes indicate genes with potential knock-out mutations (e.g. frameshifts and nonsense mutations). For *nosR*, mutational profiles were very different for cyanobacteria and eukaryotes, so two node types are included to reflect this. Node color indicates in which phytoplankton cocultures mutations were observed: Green, MIT9312; pink, CC9311; brown, both eukaryotic strains. Gene names in italics represent the names of the closest match in *Escherichia* or *Pseudomonas* genomes; capitalized letters represent genes without clearly annotated identities. TBDR, TonB-dependent receptor; MCP, methyl-accepting chemotaxis protein; PATP, PepSY-associated transmembrane protein; AFDBP, arc family DNA binding protein; CDGC, cyclic-di-GMP cyclase. Gene names in gold have products involved in transmembrane transport; names in blue have products involved in environmental sensing and transcriptional regulation. Arrow color reflects the Spearman correlation coefficient between the two genes according to the color bar at the bottom of the figure.

Relatively few *Alteromonas* genes that passed our filter were shared between *Alteromonas* evolved with *Prochlorococcus* vs. *Synechococcus* or between *Alteromonas* evolved with cyanobacteria versus eukaryotes, whereas a large suite of genes was shared between *Alteromonas* partnered with each of the two eukaryotic phytoplankton (Fig. 4C, Data S3), with strong overrepresentation for genes involved in amino acid metabolism, but also including central carbon metabolic pathways, vitamin and nucleic acid metabolism, and secretion/export pathways (Fig. 5A), suggesting a variety of possible vectors for interaction or metabolic product exchange with their phytoplankton partners.

Substantial evolution of *Alteromonas’* single plasmid was also detected, with coverage data (Table S2) suggesting that most evolved populations had significant accumulations of plasmid-free segregants (Fig. S17A), reduced-sized plasmids (Fig. S17B), or possible insertion of a fragment of the plasmid by homologous recombination into the primary chromosome (Fig. S17C). Most populations evolved alongside *Synechococcus* lost the free plasmid entirely, whereas most populations evolved with *E. huxleyi* appear to have retained it intact, with those evolved at 800 ppm pCO_2_ in some cases carrying multiple plasmid copies per cell (Fig. S19B). Overall, *Alteromonas* evolved with cyanobacteria were significantly more likely to lose the free plasmid entirely than were those evolved alongside eukaryotes (Fisher’s exact test, *P* = .009, Fig. S17A), and copy numbers were generally higher in 800 ppm pCO_2_ evolved lineages (Fig. S17A insert, linear model, *P* < .01). The *Alteromonas* EZ55 plasmid primarily consists of transposable elements, metal and antibiotic resistance genes, and genes for metabolizing toluene and xylene [50], so it is unclear what selective pressures underlie these trends.

### Adaptation of *Alteromonas* to different partners

Metrics of directional selection (Figs 4B, S11C, D), counts of multiply mutated genes (Figs 4C, S15), and overmutated pathways (Fig. 5A, S16) support the hypothesis that *Alteromonas* EZ55 rapidly evolved in response to the environment created by eukaryotic partners. We investigated whether the switch between partners was driven by the evolution of core sets of partner-specific genes by examining the correlational network of mutations in the *Alteromonas* genome across lineages (Fig. 5B). For 17 genes in the *Alteromonas* genome, the presence of mutations in one gene was significantly predictive of mutations in at least one other gene. Most of these were genes related to transcriptional regulation, environmental sensing, or transmembrane transport, and many included frameshift or nonsense mutations, suggesting that the adaptive benefit of the mutations may involve loss of function. The gene with the most incoming predictive edges was only found mutated in *Alteromonas* paired with eukaryotic phytoplankton, and it encodes a gene matching the *aceK* phosphatase/kinase that regulates the switch between the full TCA cycle and the abbreviated glyoxylate shunt in *E. coli*. Phosphorylation of isocitrate dehydrogenase by AceK bypasses the loss of 2 C atoms during oxidative growth, allowing growth on simple compounds such as acetate or glycolate [70]. Mutations in genes related to growth on complex carbon compounds strongly predicted likely knock-out mutations in *aceK* in eukaryotic cultures, suggesting a shift away from simple carbon substrates as *Alteromonas* evolves away from partnering with *Prochlorococcus*. Previous work suggested that catabolism of small organic acids was an important component of *Alteromonas’* response to short-term coculture with both *Prochlorococcus* and *Synechococcus* [6], so the inactivation of a key pathway for metabolizing these compounds in long-term coculture with eukaryotes is particularly striking. Potential loss of function mutations in genes related to denitrification (e.g. molybdenum transport gene *modF* and nitrite reductase gene *nosR*) were also predictive of mutations in the cluster of genes connecting to *aceK*, possibly signaling changes in *Alteromonas’* N demand when cocultured with eukaryotes. These changes are suggestive of broad alterations in core metabolic processes as *Alteromonas* adapts to partners other than *Prochlorococcus*.

### Evolution of the Prochlorococcus/Alteromonas “helper” interaction

**Fig. 6:**
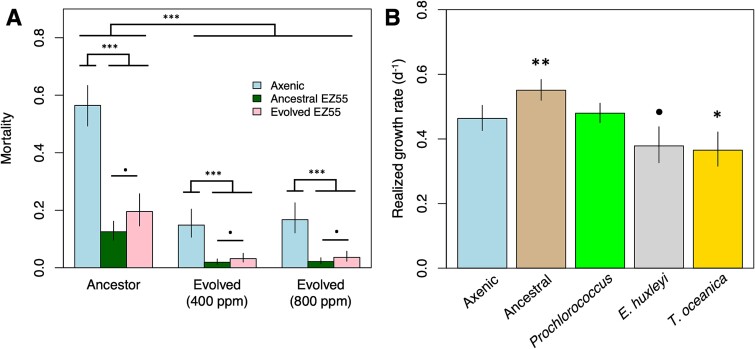
Effects of experimental evolution on the “helping” ability of *Alteromonas* EZ55. (A) Bars represent model estimates from a binomial logistic regression model predicting the likelihood of culture failure/death for *Prochlorococcus* cultures grown either axenically or as cocultures with ancestral EZ55 or with EZ55 evolved with *Prochlorococcus*. Because EZ55 clones from both pCO_2_ treatments had statistically identical effects on mortality reduction, they are included together in these estimates. The impact of pCO_2_ on mortality was independent of the EZ55 treatment and is shown in Fig. S21. (B) *Prochlorococcus* was grown either axenically, in coculture with ancestral EZ55, or with clones of EZ55 isolated from cultures of *Prochlorococcus*, *E. huxleyi*, or *T. oceanica* after 500 generations of evolution at 400 ppm pCO_2_. G and L indicate significantly (*P* < .05) greater or lower growth rates based on the results of a Dunnett’s test comparing each EZ55 treatment to the axenic control, whereas n.s. indicates the result of the comparison was nonsignificant. Error bars represent the 95% confidence intervals of the extended marginal means estimate of the growth rate. ^•^*P* < .1; ^*^P < .05; ^*^^*^P < .01, ^*^^*^^*^P < .001.

Collectively, these mutational observations (Fig. 5) suggest that the speed and extent of adaptive evolution were faster for *Alteromonas* when it was paired with partners that were more distinct from its historical partner, *Prochlorococcus*. Of the phytoplankton, only *Prochlorococcus* showed strong evidence of general directional selection (Figs 4A, S11A, B), and the fact that multiple targets related to oxidative stress (e.g. PTOX and thioredoxin reductase, Data S1) were detected suggested that the “helping” activity of *Alteromonas* may have been downgraded during evolution, forcing *Prochlorococcus* to evolve stronger antioxidant defenses of its own. These two observations raised the possibility that *Alteromonas* may specialize on particular partners, which might also impact its “helper” ability if adaptations that improve fitness with alternative partners are antagonistic to those that facilitate *Prochlorococcus*’ growth. We therefore sought to measure changes in *Alteromonas’* “helping” ability by conducting a series of experiments where various combinations of evolved and ancestral *Prochlorococcus* and *Alteromonas* were paired in coculture. First, we discovered that evolved *Prochlorococcus* cultures retained a need for “help” from *Alteromonas*, with significantly elevated mortality risk (i.e. likelihood that a culture failed to grow after transfer) in axenic culture (Fig. 6A) and at elevated pCO_2_ (Fig. S18) even after 500 generations of evolution. However, the magnitude of this impediment had significantly decreased during evolution, suggesting that at least some protective mutations (e.g. the PTOX and thioredoxin reductase regulatory mutations described above) had arisen in *Prochlorococcus* genomes. We also found that the phytoplankton partner with which *Alteromonas* evolved affected its helping ability. *Alteromonas* isolates from the evolved *Prochlorococcus* cultures (regardless of evolutionary pCO_2_ treatment) were generally less effective as helpers of ancestral *Prochlorococcus*, allowing greater mortality than ancestral *Alteromonas* (Fig. 6A). Moreover, whereas the *Alteromonas* ancestor increased the *Prochlorococcus* growth rate relative to axenic cultures, the evolved *Alteromonas* isolates either did not (Figs 6B, S19). In fact, *Alteromonas* evolved alongside eukaryotes actually slowed the growth rates of *Prochlorococcus* relative to axenic strains (Figs 6B, S19), suggesting that the divergent evolutionary trajectory of *Alteromonas* with eukaryotes had a tradeoff in terms of its ability to facilitate *Prochlorococcus*’ growth.

## Discussion

We draw four central conclusions from the results of our evolution experiment. First, we observed rapid directional evolution in all the cultures we observed. Growth rates in our cyanobacterial cultures increased significantly in both pCO_2_ conditions (Figs 1 and 2). Evolution under the 400 ppm regime likely reflected adaptation to the novel culture conditions of the experiment independently of pCO_2_, e.g. the presence of *Alteromonas*, the low nutrient medium, or the regular dilution schedule. *Prochlorococcus*, which had the most negative response to future pCO_2_ conditions prior to evolution, was able to compensate for its growth deficiencies within 500 generations of growth at year 2100 pCO_2_ (Fig. 2A), suggesting that in situ populations will be able to evolve fast enough to avoid major changes to their range in coming decades (e.g. [4]). Whereas the growth rates of eukaryotic phytoplankton were less affected by evolution, genomic evidence indicated that their *Alteromonas* partners experienced strong directional evolution (Figs 4B, S11C, D), especially under elevated pCO_2_ (Fig. S15).

A second conclusion was that correlated growth rate responses after evolution suggested important differences between our two eukaryotic phytoplankton (Fig. 3). In the case of the diatom *T. oceanica*, growth rates increased under the evolutionary pCO_2_ condition but decreased under the opposite condition, providing evidence of a physiological trade-off and specialization on a particular pCO_2_ regime. On the other hand, after 500 generations of growth at 800 ppm pCO_2_, *E. huxleyi*’s growth rates were higher under both pCO_2_ treatments than lineages evolved at 400 ppm pCO_2_, indicating cost-free adaptation. This observation is consistent with previous work showing that *E. huxleyi* evolved at elevated pCO_2_ did not show significantly decreased growth rates under current conditions [24]. Further work will be necessary to understand the metabolic basis for these differences. The very different profile of mutations in *T. oceanica* lineages between the two pCO_2_ treatments (Fig. S14) may provide insight into the tradeoffs, and it is possible that these metabolic alterations will cause unexpected secondary effects relevant to community function and nutrient cycling [71–73].

A third conclusion was that there was striking evidence of partner-specific, and to a lesser degree pCO_2_-specific, evolution in *Alteromonas*. Metrics of directional selection were much stronger in *Alteromonas* paired with eukaryotes (Figs 4B, S11C, D), and a very different suite of genes were convergently mutated with eukaryotes than with cyanobacteria (Figs 5A, S16), especially at elevated pCO_2_ (Fig. S15). The presence of mutations in particular genes was predictive of mutations in a suite of other genes (Fig. 5B), with a possibly causal cascade of changes (i.e. many paths leading to the same mutations) occurring during adaptation to growth with eukaryotic phytoplankton. Other studies have shown that strains of *Alteromonas macleodii* including EZ55 are metabolically diverse and specialized for a wide variety of environments, underlying their ubiquity in marine habitats [50]. Our results demonstrate that *Alteromonas* EZ55 has remarkable evolutionary flexibility even without gaining new genetic material by horizontal gene transfer. This work also demonstrates under controlled conditions the process of ecotype differentiation observed in natural populations of *Vibrio* [74] and underscores the importance of considering the evolutionary potential of heterotrophic bacteria in the context of ecological response to anthropogenic change in the ocean.

As a fourth and final conclusion, we believe that our results conclusively demonstrate that the “helper” interaction between *Alteromonas* and *Prochlorococcus* is not strictly or stably mutualistic. Instead, we saw evidence that each organism adapted to improve its own fitness and potentially disentangle itself from its partner. For instance, *Prochlorococcus* clearly became less dependent on *Alteromonas* for stress protection, evidenced by its reduced mortality risk in axenic culture compared to its ancestor (Fig. 6A). At the same time, *Alteromonas’* capacity to protect *Prochlorococcus* decreased even in clones isolated from evolved *Prochlorococcus* cultures, and this effect was more pronounced after coevolution with more distantly related phytoplankton partners (Fig. 6B). In previous work involving short-term cocultures [6, 7] we showed that *Prochlorococcus*’ poor growth at 800 ppm occurred because *Alteromonas* decreased expression of its catalase genes under these conditions, possibly because of changes in the carbon compounds *Prochlorococcus* excreted under a higher CO_2_:O_2_ ratio atmosphere. We hypothesized that, over evolutionary time, *Alteromonas* might adjust its gene expression to restore its “helper” ability at 800 ppm pCO_2_, but instead the evidence suggests that the two partners evolved greater independence from each other. This result is consistent with the concept that the outwardly mutualistic interaction between *Prochlorococcus* and helpers like *Alteromonas* is a result of reductive Black Queen evolution [75, 76]. Whereas true mutualisms are positively reinforcing, Black Queen mutualisms are the result of dynamic equilibria between a more efficient, streamlined beneficiary and a helper. Laboratory experiments show that these equilibria fluctuate as one partner or the other obtains beneficial mutations during evolution [77, 78]. Both chemostat [79] and transcriptomic evidence [6] indicate that *Alteromonas* competes for N with phytoplankton in coculture, so it is reasonable to expect that its apparent “helping” ability should decrease as it evolves to more efficiently compete with *Prochlorococcus*, but that the general dynamic would persist. An open question, however, is why *Prochlorococcus* in more complex natural assemblages does not appear to favor the putative antioxidant mutations observed in this experiment, but instead maintains a much higher degree of vulnerability to oxidative stress than is strictly necessary given its genomic capacities.

In conclusion, this work demonstrates that phytoplankton are sufficiently evolutionarily plastic that results from short-term experiments are unlikely to be strong predictors of the behavior of taxa under future pCO_2_ conditions, and likely other environmental changes such as warming or changes in light or nutrient regimes as well. However, some taxa may be extensively modified during this adaptation as evidenced by the hundreds of nonsynonymous mutations sampled during this relatively short period, and metabolic changes are likely to alter the character of exuded photosynthates which will result in compensatory alterations in pelagic bacterial communities with unknown impacts on the rest of the food web. Finally, we see evidence of rapid diversification and partner specialization in *Alteromonas*, suggesting that this ubiquitous taxon may undergo similar evolution in nature in response to local phytoplankton communities. Future work should examine gene expression and metabolomics to explore the mechanisms underlying the shift for *Alteromonas* between cyanobacteria and eukaryote specialization, and why this shift results in apparently exploitative interactions with *Prochlorococcus*. We propose that the evolved organisms from this experiment present an opportunity for future investigators to study this phenomenon and other aspects of algal:bacterial coevolution in greater detail.

## Supplementary Material

Lu_Supplemental_Text_wrae259

DataS1_wrae259

DataS2_wrae259

DataS3_wrae259

## Data Availability

Sequence data are available via Genbank and SRA archives at NCBI, using accession numbers described in the Materials and Methods. All code and raw data necessary to replicate the analyses described in this paper are permanently archived at BCO-DMO (DOI 10.26008/1912/bco-dmo.925841 and 10.26008/1912/bco-dmo.925872). Ancestral and evolved populations of phytoplankton and bacteria are cryopreserved at UAB and are available to qualified researchers upon request.
