# Supplementary material for: Coevolution of marine phytoplankton and Alteromonas bacteria in response to pCO2 and coculture
Source: ISME J. 2024 Dec 24;19(1):wrae259. doi: 10.1093/ismejo/wrae259 (PMC11748131; doi:10.1093/ismejo/wrae259)
Supplement: Lu_Supplemental_Text_wrae259 [file lu_supplemental_text_wrae259.pdf]

Supplemental Material for:

Coevolution of marine phytoplankton and *Alteromonas* bacteria in response to pCO_2 and co-culture

Zhiying Lu[1], Elizabeth Entwistle[1], Matthew D. Kuhl[1], Alexander R. Durrant[1], Marcelo Malisano Barreto Filho[1], Anuradha Goswami[1], and J. Jeffrey Morris[1,*]

[1] Department of Biology, University of Alabama at Birmingham
[*] Author for correspondence: evolve@uab.edu

Materials included:

Tables S1 – S6

Figures S1 – S30

Data S1 – S3 are provided as .csv files for download

Table S1. Culture properties.

Strain	Designation	Organism	Partner	pCO2	Medium	Transfer Density[1]	Start Date	End Date	Transfers	Generations	Crash Restarts[2]	Contamination Restarts[2]	Move Restarts[2]
LTPE191	C-1	Synechocystis PCC6803	None	400ppm	SEv	2.60E+05	8/21/14	5/16/17	125	588	0	3	1
LTPE192	C-2	Synechocystis PCC6803	None	400ppm	SEv	2.60E+05	8/14/14	8/7/17	129	606	0	3	1
LTPE193	C-3	Synechocystis PCC6803	None	400ppm	SEv	2.60E+05	8/14/14	8/7/17	136	639	0	4	1
LTPE194	C-4	Synechocystis PCC6803	None	400ppm	SEv	2.60E+05	8/21/14	8/9/17	133	625	0	2	1
LTPE195	C-5	Synechocystis PCC6803	None	400ppm	SEv	2.60E+05	8/21/14	8/4/17	129	606	0	4	1
LTPE196	C+1	Synechocystis PCC6803	None	800ppm	SEv	2.60E+05	8/21/14	7/12/17	129	606	0	3	1
LTPE197	C+2	Synechocystis PCC6803	None	800ppm	SEv	2.60E+05	8/14/14	8/20/17	107	503	0	3	1
LTPE198	C+3	Synechocystis PCC6803	None	800ppm	SEv	2.60E+05	8/14/14	8/22/17	120	564	0	2	1
LTPE199	C+4	Synechocystis PCC6803	None	800ppm	SEv	2.60E+05	8/20/14	8/7/17	117	550	0	4	1
LTPE200	C+5	Synechocystis PCC6803	None	800ppm	SEv	2.60E+05	8/21/14	8/7/17	121	569	0	3	1
LTPE397	M-1	Prochlorococcus MIT9312	Alteromonas EZ55	400ppm	PEv	2.60E+06	1/23/16	7/1/18	108	508	5	0	0
LTPE398	M-2	Prochlorococcus MIT9312	Alteromonas EZ55	400ppm	PEv	2.60E+06	1/10/16	10/31/18	77	362	3	1	0
LTPE399	M-3	Prochlorococcus MIT9312	Alteromonas EZ55	400ppm	PEv	2.60E+06	1/10/16	4/14/18	107	503	1	0	0
LTPE400	M-4	Prochlorococcus MIT9312	Alteromonas EZ55	400ppm	PEv	2.60E+06	1/10/16	10/15/18	90	423	3	0	0
LTPE401	M-5	Prochlorococcus MIT9312	Alteromonas EZ55	400ppm	PEv	2.60E+06	1/10/16	4/7/18	108	508	2	0	0
LTPE402	M-6	Prochlorococcus MIT9312	Alteromonas EZ55	400ppm	PEv	2.60E+06	1/21/16	2/17/18	108	508	0	0	0
LTPE403	M+1	Prochlorococcus MIT9312	Alteromonas EZ55	800ppm	PEv	2.60E+06	1/23/16	10/15/18	108	508	4	1	0
LTPE404	M+2	Prochlorococcus MIT9312	Alteromonas EZ55	800ppm	PEv	2.60E+06	1/17/16	6/29/18	108	508	2	1	0
LTPE405	M+3	Prochlorococcus MIT9312	Alteromonas EZ55	800ppm	PEv	2.60E+06	1/10/16	8/12/18	108	508	3	1	0
LTPE406	M+4	Prochlorococcus MIT9312	Alteromonas EZ55	800ppm	PEv	2.60E+06	1/10/16	12/3/18	93	437	3	1	0
LTPE407	M+5	Prochlorococcus MIT9312	Alteromonas EZ55	800ppm	PEv	2.60E+06	1/10/16	10/15/18	73	343	3	1	0
LTPE408	M+6	Prochlorococcus MIT9312	Alteromonas EZ55	800ppm	PEv	2.60E+06	1/17/16	6/25/18	108	508	3	1	0
LTPE421	W-1	Synechococcus CC9311	Alteromonas EZ55	400ppm	SEv	2.60E+05	6/20/16	6/19/18	107	503	3	0	0
LTPE422	W-2	Synechococcus CC9311	Alteromonas EZ55	400ppm	SEv	2.60E+05	6/20/16	3/12/18	107	503	3	0	0
LTPE423	W-3	Synechococcus CC9311	Alteromonas EZ55	400ppm	SEv	2.60E+05	6/20/16	6/3/18	107	503	5	0	0
LTPE424	W-4	Synechococcus CC9311	Alteromonas EZ55	400ppm	SEv	2.60E+05	6/20/16	12/3/18	96	451	3	0	0
LTPE425	W-5	Synechococcus CC9311	Alteromonas EZ55	400ppm	SEv	2.60E+05	6/20/16	6/3/18	107	503	3	0	0
LTPE426	W-6	Synechococcus CC9311	Alteromonas EZ55	400ppm	SEv	2.60E+05	6/20/16	4/2/18	107	503	2	0	0
LTPE427	W+1	Synechococcus CC9311	Alteromonas EZ55	800ppm	SEv	2.60E+05	6/28/16	7/13/18	107	503	3	1	0
LTPE428	W+2	Synechococcus CC9311	Alteromonas EZ55	800ppm	SEv	2.60E+05	6/28/16	5/7/18	107	503	2	1	0
LTPE429	W+3	Synechococcus CC9311	Alteromonas EZ55	800ppm	SEv	2.60E+05	6/28/16	5/9/18	107	503	1	1	0
LTPE430	W+4	Synechococcus CC9311	Alteromonas EZ55	800ppm	SEv	2.60E+05	6/28/16	9/11/18	107	503	7	1	0
LTPE431	W+5	Synechococcus CC9311	Alteromonas EZ55	800ppm	SEv	2.60E+05	6/28/16	4/19/18	107	503	2	1	0
LTPE432	W+6	Synechococcus CC9311	Alteromonas EZ55	800ppm	SEv	2.60E+05	6/28/16	6/12/18	107	503	5	1	0
LTPE445	T-1	Thalassiosira oceanica CCMP1005	Alteromonas EZ55	400ppm	FEv	2.60E+04	5/10/17	4/2/18	107	503	1	0	0
LTPE446	T-2	Thalassiosira oceanica CCMP1005	Alteromonas EZ55	400ppm	FEv	2.60E+04	5/10/17	4/7/18	107	503	1	0	0
LTPE447	T-3	Thalassiosira oceanica CCMP1005	Alteromonas EZ55	400ppm	FEv	2.60E+04	5/10/17	4/14/18	107	503	1	0	0
LTPE448	T-4	Thalassiosira oceanica CCMP1005	Alteromonas EZ55	400ppm	FEv	2.60E+04	5/10/17	5/11/18	107	503	1	0	0
LTPE449	T-5	Thalassiosira oceanica CCMP1005	Alteromonas EZ55	400ppm	FEv	2.60E+04	5/10/17	4/16/18	106	498	1	0	0
LTPE450	T-6	Thalassiosira oceanica CCMP1005	Alteromonas EZ55	400ppm	FEv	2.60E+04	5/10/17	5/11/18	107	503	1	0	0
LTPE451	T+1	Thalassiosira oceanica CCMP1005	Alteromonas EZ55	800ppm	FEv	2.60E+04	5/10/17	5/5/18	108	508	1	0	0
LTPE452	T+2	Thalassiosira oceanica CCMP1005	Alteromonas EZ55	800ppm	FEv	2.60E+04	5/10/17	5/12/18	107	503	1	0	0
LTPE453	T+3	Thalassiosira oceanica CCMP1005	Alteromonas EZ55	800ppm	FEv	2.60E+04	5/10/17	4/19/18	107	503	1	0	0
LTPE454	T+4	Thalassiosira oceanica CCMP1005	Alteromonas EZ55	800ppm	FEv	2.60E+04	5/10/17	6/9/18	107	503	1	0	0
LTPE455	T+5	Thalassiosira oceanica CCMP1005	Alteromonas EZ55	800ppm	FEv	2.60E+04	5/10/17	6/14/18	107	503	1	1	0
LTPE456	T+6	Thalassiosira oceanica CCMP1005	Alteromonas EZ55	800ppm	FEv	2.60E+04	5/10/17	5/9/18	107	503	1	0	0
LTPE469	E-1	Emiliania huxleyi CCMP371	Alteromonas EZ55	400ppm	FEv	2.60E+04	5/11/17	6/23/18	108	508	1	0	0
LTPE470	E-2	Emiliania huxleyi CCMP371	Alteromonas EZ55	400ppm	FEv	2.60E+04	5/12/17	6/28/18	107	503	1	0	0
LTPE471	E-3	Emiliania huxleyi CCMP371	Alteromonas EZ55	400ppm	FEv	2.60E+04	5/13/17	7/9/18	107	503	1	0	0
LTPE472	E-4	Emiliania huxleyi CCMP371	Alteromonas EZ55	400ppm	FEv	2.60E+04	5/14/17	7/20/18	107	503	1	0	0
LTPE473	E-5	Emiliania huxleyi CCMP371	Alteromonas EZ55	400ppm	FEv	2.60E+04	5/15/17	7/20/18	107	503	1	0	0
LTPE474	E-6	Emiliania huxleyi CCMP371	Alteromonas EZ55	400ppm	FEv	2.60E+04	5/16/17	7/14/18	108	508	1	0	0
LTPE475	E+1	Emiliania huxleyi CCMP371	Alteromonas EZ55	800ppm	FEv	2.60E+04	5/17/17	8/30/18	107	503	0	2	0
LTPE476	E+2	Emiliania huxleyi CCMP371	Alteromonas EZ55	800ppm	FEv	2.60E+04	5/18/17	8/18/18	107	503	0	2	0
LTPE477	E+3	Emiliania huxleyi CCMP371	Alteromonas EZ55	800ppm	FEv	2.60E+04	5/19/17	8/24/18	107	503	0	2	0
LTPE478	E+4	Emiliania huxleyi CCMP371	Alteromonas EZ55	800ppm	FEv	2.60E+04	5/20/17	9/13/18	107	503	0	2	0
LTPE479	E+5	Emiliania huxleyi CCMP371	Alteromonas EZ55	800ppm	FEv	2.60E+04	5/21/17	9/30/18	107	503	0	2	0
LTPE480	E+6	Emiliania huxleyi CCMP371	Alteromonas EZ55	800ppm	FEv	2.60E+04	5/22/17	9/8/18	107	503	0	2	0

[1] Cell density (in cells mL^{-1}) at which a culture was transferred into fresh media. In practice, cultures were generally transferred at cell densities up to 50% of this value to avoid overshooting.

[2] How often the lineage had to be restarted from a backup culture or freezer stock due to failure to grow (crash), detection of contaminants, or lab relocation. See Materials and Methods for details.

Table S2. Genomic re-sequencing coverage.

Culture	Phytoplankton Coverage[1]	EZ55 Chromosome Coverage	EZ55 Plasmid Coverage[2]	pSYSA coverage	pSYSG coverage	pSYSM Coverage	pSYSX coverage
PCC6803	102 +/- 20.9	n/a	n/a	111 +/- 14.1	111 +/- 15.4	146 +/- 16.3	142 +/- 14.1
MIT9312	253 +/- 86.6	112 +/- 19.8	72.2 +/- 22.6	n/a	n/a	n/a	n/a
CC9311	123 +/- 52.4	613 +/- 194	271 +/- 121	n/a	n/a	n/a	n/a
CCMP371	20.9 +/- 4.16	491 +/- 255	426.5 +/- 250	n/a	n/a	n/a	n/a
CCMP1005	32.5 +/- 6.37	567 +/- 215	293 +/- 89.0	n/a	n/a	n/a	n/a

[1] Coverage values are given as means plus/minus 95% confidence intervals of replicate lineages.
[2] EZ55 plasmid coverage is given as the average estimate across the entire ancestral plasmid. See text for descriptions of various plasmid variants that may have evolved.

Table S3. Mutation calling workflow in cyanobacterial genomes. "Total" indicates the number of mutations identified by breseq, excluding putative new junctions. "Ancestral" indicates the number of mutations shared across all genomes. "Final" indicates the number of mutations considered for downstream analysis.

		pCO2	Total	Ancestral	Final
Synechocystis	LTPE191	400 ppm	336	26	310
	LTPE192	400 ppm	290	26	264
	LTPE193	400 ppm	637	26	611
	LTPE194	400 ppm	163	26	137
	LTPE195	400 ppm	708	26	682
	LTPE196	800 ppm	321	26	295
	LTPE197	800 ppm	334	26	308
	LTPE198	800 ppm	301	26	275
	LTPE199	800 ppm	530	26	504
	LTPE200	800 ppm	327	26	301
Prochlorococcus	LTPE397	400 ppm	217	23	194
	LTPE398	400 ppm	65	23	42
	LTPE399	400 ppm	252	23	229
	LTPE400	400 ppm	89	23	66
	LTPE401	400 ppm	57	23	34
	LTPE402	400 ppm	70	23	47
	LTPE403	800 ppm	114	23	91
	LTPE404	800 ppm	60	23	37
	LTPE405	800 ppm	126	23	103
	LTPE406	800 ppm	86	23	63
	LTPE407	800 ppm	76	23	53
	LTPE408	800 ppm	54	23	31
Synechococcus	LTPE421	400 ppm	410	35	375
	LTPE422	400 ppm	442	35	407
	LTPE423	400 ppm	159	35	124
	LTPE424	400 ppm	124	35	89
	LTPE425	400 ppm	313	35	278
	LTPE426	400 ppm	267	35	232
	LTPE427	800 ppm	109	35	74
	LTPE428	800 ppm	235	35	200
	LTPE429	800 ppm	154	35	119
	LTPE430	800 ppm	115	35	80
	LTPE431	800 ppm	558	35	523
	LTPE432	800 ppm	449	35	414

Table S4. Mutation calling workflow in eukaryotic phytoplankton genomes. Column A is the number of mutations called by breseq; Column B is the number of mutations in A that were also found in our re-assembly of the reference genome; Column D are differences we found in the reference genome that were NOT found by breseq in the re-sequenced genomes; Column F indicates the number of breseq-called mutations that were identified as heterozygous in the re-assembled genome; Column G is the number of those heterozygous loci that remained heterozygous in the re-sequenced genomes; Column I indicates loci that were heterozygous in the re-assembled reference but were fixed in the re-sequenced gnome; and Column K are the number of putative ancestral mutations shared amongst all strains relative to the reference genome. Column L shows the number of mutations considered in our downstream analyses.

			A	B	C	D	E	F	G	H	I	J	K	L
		**pCO2**	**Total**	**Same as refseq**	**Refseq removed (A-B)**	**Diff. from refseq**	**Diff. added back (C+D)**	**Hetero-zygous in refseq**	**Still hetero-zygous**	**Without hetero-zygous (E-G)**	**Hetero-zygous that fixed**	**All (H+I)**	**Ancestral**	**All minus ancestral (J-K)**
T. oceanica	LTPE445	400 ppm	717076	825	716251	3654	719905	67395	66276	653629	29161	682790	23027	659763
	LTPE446	400 ppm	664468	836	663632	3643	667275	64990	63753	603522	31566	635088	23027	612061
	LTPE447	400 ppm	694471	813	693658	3666	697324	65931	64719	632605	30625	663230	23027	640203
	LTPE448	400 ppm	777339	840	776499	3639	780138	70266	69261	710877	26290	737167	23027	714140
	LTPE449	400 ppm	832426	845	831581	3634	835215	72342	71343	763872	24214	788086	23027	765059
	LTPE450	400 ppm	590826	825	590001	3654	593655	61739	60180	533475	34817	568292	23027	545265
	LTPE451	800 ppm	767716	853	766863	3626	770489	70280	69317	701172	26276	727448	23027	704421
	LTPE452	800 ppm	933689	837	932852	3642	936494	74450	73651	862843	22106	884949	23027	861922
	LTPE453	800 ppm	861730	841	860889	3638	864527	72909	72042	792485	23647	816132	23027	793105
	LTPE454	800 ppm	969475	831	968644	3648	972292	75315	74564	897728	21241	918969	23027	895942
	LTPE455	800 ppm	762518	868	761650	3611	765261	69701	68461	696800	26855	723655	23027	700628
	LTPE456	800 ppm	875083	868	874215	3611	877826	72928	71938	805888	23628	829516	23027	806489
E. huxleyi	LTPE469	400 ppm	848653	62304	786349	16820	803169	87352	85410	717759	44928	762687	14762	747925
	LTPE470	400 ppm	194850	34327	160523	44584	205107	37020	34437	170670	95260	265930	14762	251168
	LTPE471	400 ppm	895197	63041	832156	15888	848044	90111	88316	759728	42169	801897	14762	787135
	LTPE472	400 ppm	264985	40622	224363	38098	262461	45884	43058	219403	86396	305799	14762	291037
	LTPE473	400 ppm	705864	59835	646029	19149	665178	81206	79018	586160	51074	637234	14762	622472
	LTPE474	400 ppm	732247	60385	671862	18315	690177	82673	80399	609778	49607	659385	14762	644623
	LTPE475	800 ppm	521591	53624	467967	25169	493136	68133	65669	427467	64147	491614	14762	476852
	LTPE476	800 ppm	70562	17155	53407	61669	115076	17822	16154	98922	114458	213380	14762	198618
	LTPE477	800 ppm	1017528	64457	953071	14265	967336	94267	92657	874679	38013	912692	14762	897930
	LTPE478	800 ppm	896996	63193	833803	15508	849311	91138	89365	759946	41142	801088	14762	786326
	LTPE479	800 ppm	984549	63975	920574	14541	935115	94242	92582	842533	38038	880571	14762	865809
	LTPE480	800 ppm	850266	62276	787990	16519	804509	88410	86511	717998	43870	761868	14762	747106

Table S5. Mutations in *Alteromonas* genomes. The first column indicates the phytoplankton species with which *Alteromonas* was paired during evolution. There were no mutations shared across all *Alteromonas* genomes, so we conclude that the reference genome and our ancestral genomes were identical.

		**pCO2**	**Mutations**
Prochlorococcus	LTPE397	400 ppm	620
	LTPE398	400 ppm	197
	LTPE399	400 ppm	715
	LTPE400	400 ppm	1876
	LTPE401	400 ppm	135
	LTPE402	400 ppm	481
	LTPE403	800 ppm	225
	LTPE404	800 ppm	160
	LTPE405	800 ppm	498
	LTPE406	800 ppm	235
	LTPE407	800 ppm	587
	LTPE408	800 ppm	461
Synechococcus	LTPE421	400 ppm	696
	LTPE422	400 ppm	408
	LTPE423	400 ppm	224
	LTPE424	400 ppm	250
	LTPE425	400 ppm	434
	LTPE426	400 ppm	388
	LTPE427	800 ppm	133
	LTPE428	800 ppm	165
	LTPE429	800 ppm	204
	LTPE430	800 ppm	176
	LTPE431	800 ppm	404
	LTPE432	800 ppm	604
T. oceanica	LTPE445	400 ppm	1996
	LTPE446	400 ppm	1413
	LTPE447	400 ppm	1170
	LTPE448	400 ppm	217
	LTPE449	400 ppm	175
	LTPE450	400 ppm	616
	LTPE451	800 ppm	1804
	LTPE452	800 ppm	1787
	LTPE453	800 ppm	1568
	LTPE454	800 ppm	1293
	LTPE455	800 ppm	1289
	LTPE456	800 ppm	281
E. huxleyi	LTPE469	400 ppm	206
	LTPE470	400 ppm	263
	LTPE471	400 ppm	224
	LTPE472	400 ppm	2376
	LTPE473	400 ppm	243
	LTPE474	400 ppm	2681
	LTPE475	800 ppm	1192
	LTPE476	800 ppm	728
	LTPE477	800 ppm	1366
	LTPE478	800 ppm	2043
	LTPE479	800 ppm	3332
	LTPE480	800 ppm	772

Table S6. Mutational multiplicity.

Species	Partner	Mutation Type	Mutations observed[1]	Maximum observed mutations[2]	Maximum dummy mutations[3]
MIT9312	EZ55	Synonymous	104	8	4
		Nonsynonymous	389	20	6
CC9311	EZ55	Synonymous	802	98	9
		Nonsynonymous	1,382	36	11
CCMP1005	EZ55	Synonymous	1,475,350	3,297	864
		Nonsynonymous	1,648,927	2,603	975
CCMP371	EZ55	Synonymous	536,313	13,634	954
		Nonsynonymous	570,103	9,028	996
PCC6803	None	Synonymous	1,689	226	14
		Nonsynonymous	1,358	87	11
EZ55	MIT9312	Synonymous	1,875	93	13
		Nonsynonymous	1,638	31	14
EZ55	CC9311	Synonymous	463	48	7
		Nonsynonymous	1,427	43	12
EZ55	CCMP1005	Synonymous	1,665	22	13
		Nonsynonymous	8,499	69	48
EZ55	CCMP371	Synonymous	2,573	88	21
		Nonsynonymous	9,618	66	52

[1] Sum of all unique mutations observed across replicate lineages.
[2] Maximum number of mutations observed in a single coding sequence across all replicate lineages
[3] Maximum number of mutations observed in a single coding sequence in any of 100 bootstrapped monte carlo mutational distributions (see methods)

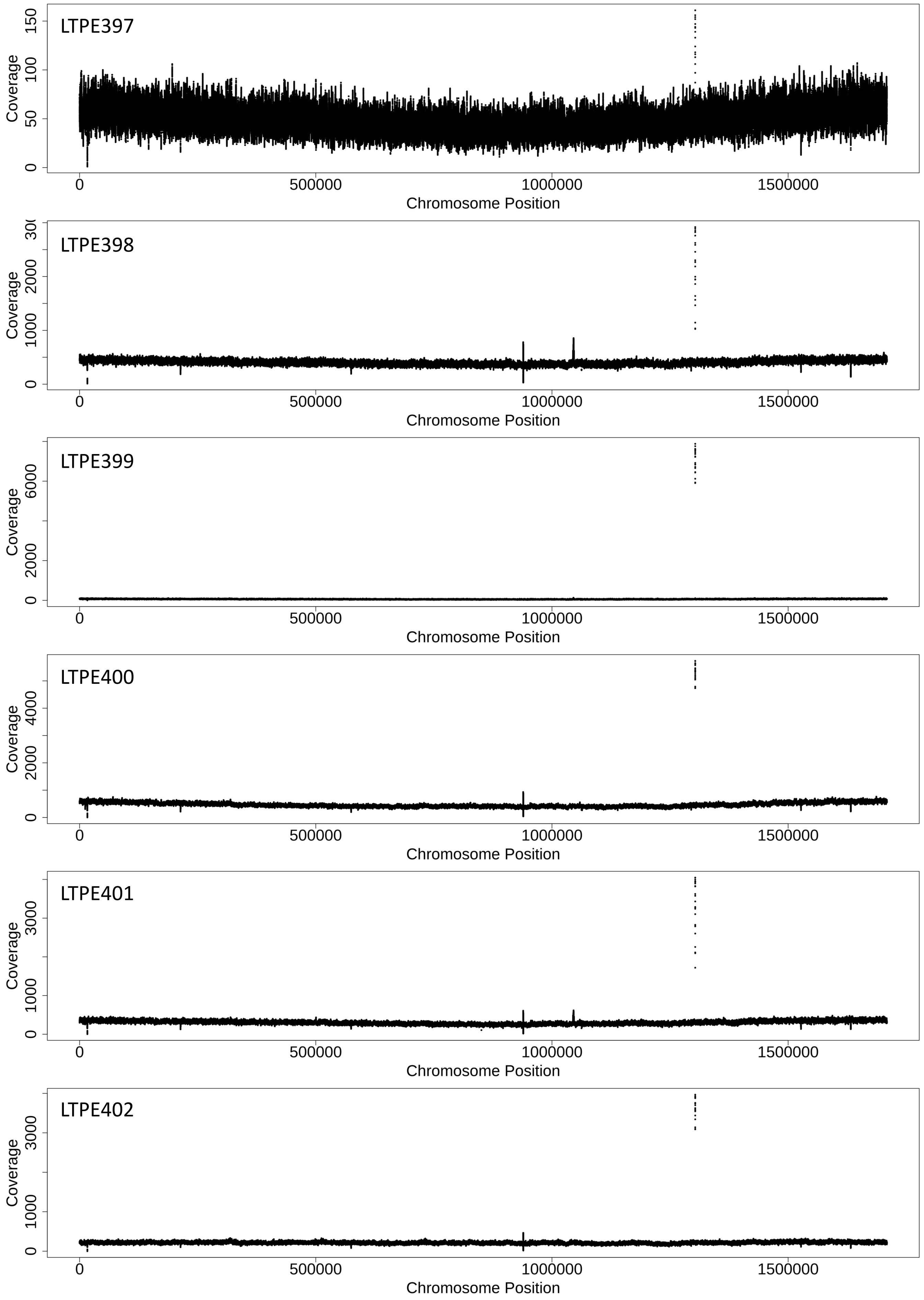
LTPE397
Coverage
0
50
100
150
0
500000
1000000
1500000
Chromosome Position
LTPE398
Coverage
0
1000
2000
0
500000
1000000
1500000
Chromosome Position
LTPE399
Coverage
0
2000
6000
0
500000
1000000
1500000
Chromosome Position
LTPE400
Coverage
0
2000
4000
0
500000
1000000
1500000
Chromosome Position
LTPE401
Coverage
0
1000
3000
0
500000
1000000
1500000
Chromosome Position
LTPE402
Coverage
0
1000
3000
0
500000
1000000
1500000
Chromosome Position

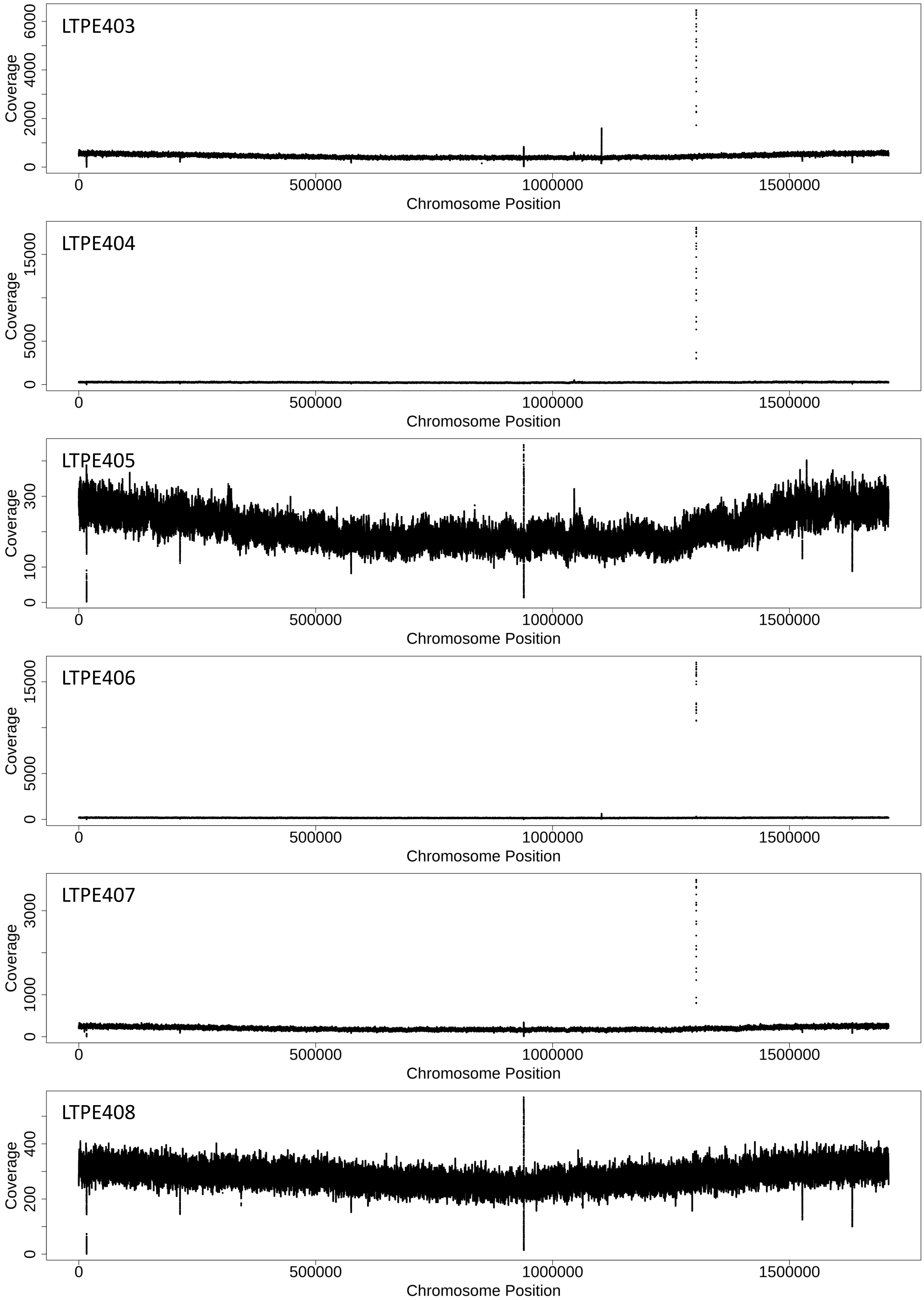
LTPE403
LTPE404
LTPE405
LTPE406
LTPE407
LTPE408
Coverage
Chromosome Position
0
2000
4000
6000
5000
15000
100
300
1000
3000
200
400
500000
1000000
1500000

Figure S1. Sequencing coverage for evolved *Prochlorococcus* MIT9312 genomes. Strains LTPE397 to LTPE402 were evolved at 400 ppm pCO_2; strains LTPE403 to LTPE408 were evolved at 800 ppm pCO_2. The spikes in coverage in most genomes around 1.3MB correspond to AT duplications in the promoter/5' coding sequence of the apolipoprotein N-acyltransferase gene described in the text.

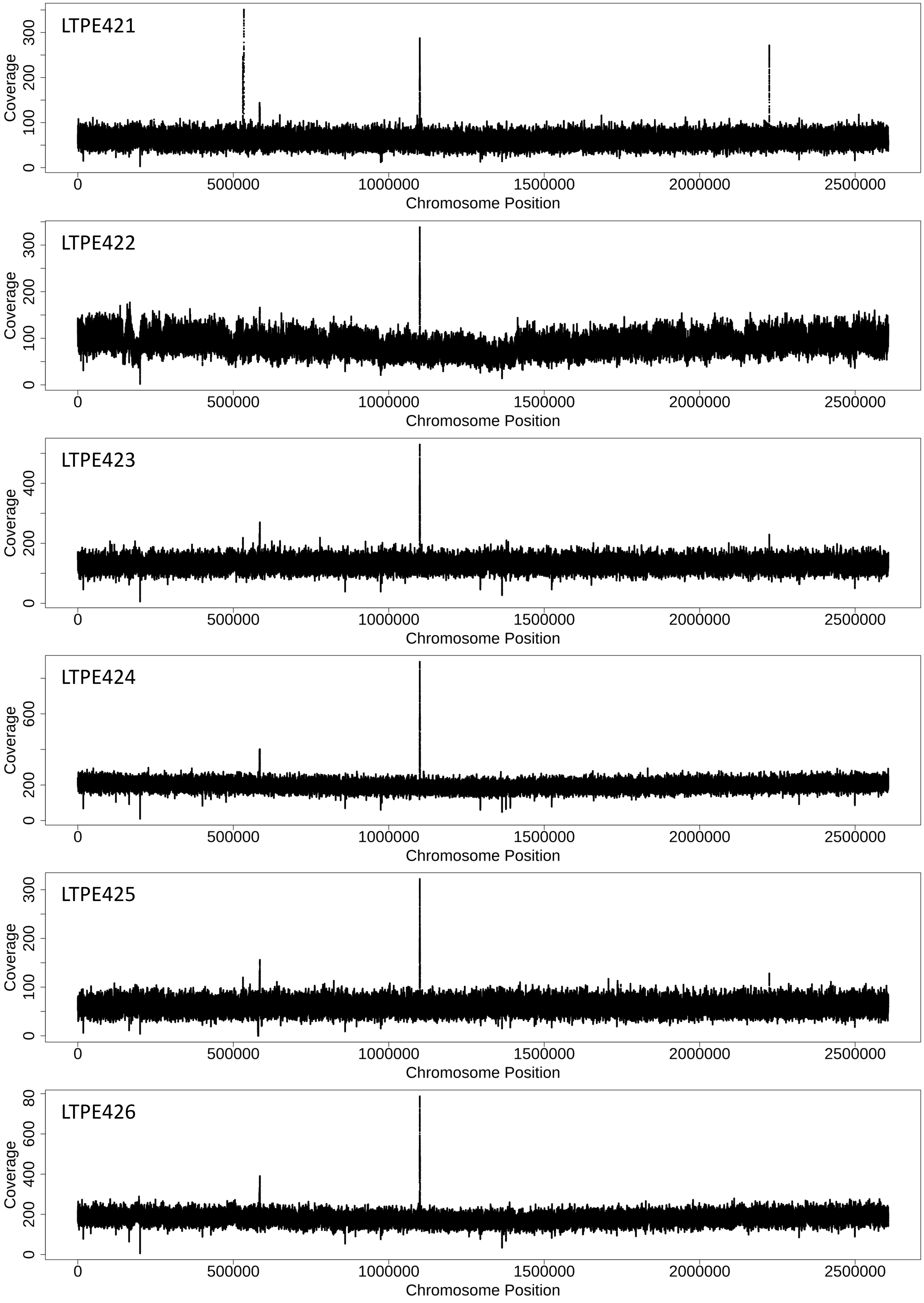
LTPE421
Coverage
0
100
200
300
0
500000
1000000
1500000
2000000
2500000
Chromosome Position
LTPE422
Coverage
0
100
200
300
0
500000
1000000
1500000
2000000
2500000
Chromosome Position
LTPE423
Coverage
0
200
400
0
500000
1000000
1500000
2000000
2500000
Chromosome Position
LTPE424
Coverage
0
200
600
0
500000
1000000
1500000
2000000
2500000
Chromosome Position
LTPE425
Coverage
0
100
200
300
0
500000
1000000
1500000
2000000
2500000
Chromosome Position
LTPE426
Coverage
0
200
400
600
80
0
500000
1000000
1500000
2000000
2500000
Chromosome Position

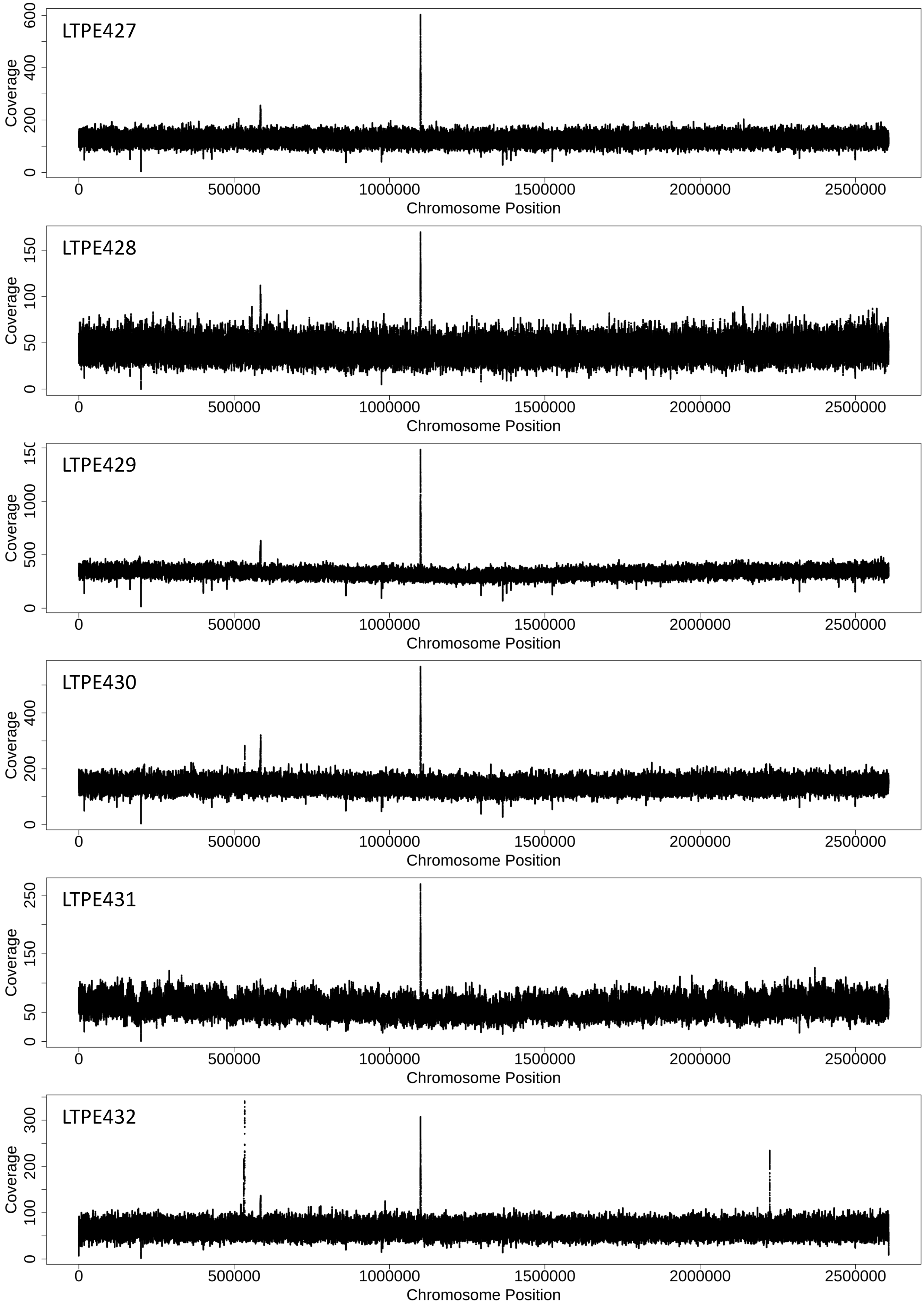
LTPE427
Coverage
0
200
400
600
0
500000
1000000
1500000
2000000
2500000
Chromosome Position
LTPE428
Coverage
0
50
100
150
0
500000
1000000
1500000
2000000
2500000
Chromosome Position
LTPE429
Coverage
0
500
1000
150
0
500000
1000000
1500000
2000000
2500000
Chromosome Position
LTPE430
Coverage
0
200
400
0
500000
1000000
1500000
2000000
2500000
Chromosome Position
LTPE431
Coverage
0
50
150
250
0
500000
1000000
1500000
2000000
2500000
Chromosome Position
LTPE432
Coverage
0
100
200
300
0
500000
1000000
1500000
2000000
2500000
Chromosome Position

Figure S2. Sequencing coverage for evolved *Synechococcus* CC9311 genomes. Strains LTPE421 to LTPE426 were evolved at 400 ppm pCO_2; strains LTPE427 to LTPE432 were evolved at 800 ppm pCO_2.

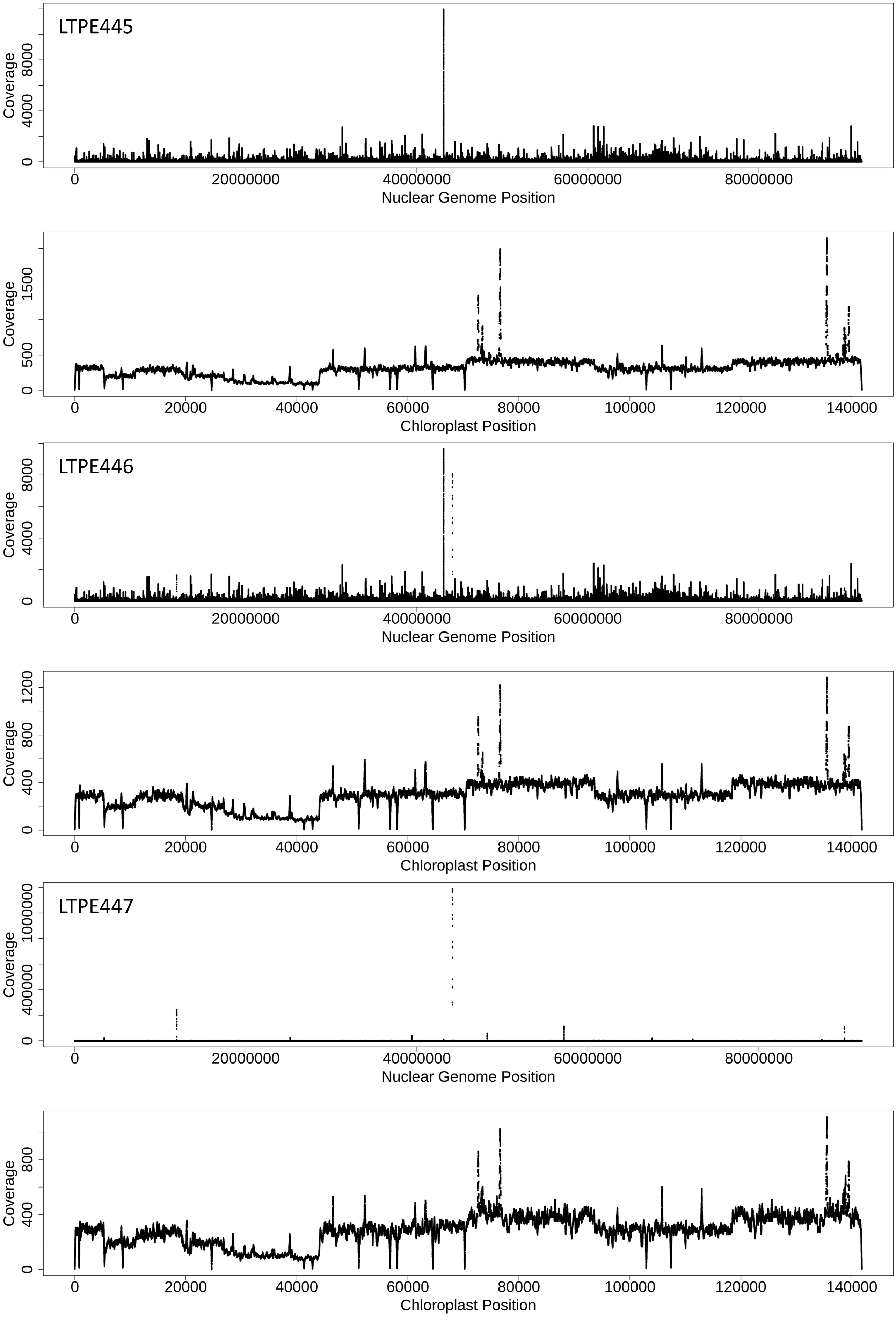

LTPE445
Coverage
0
4000
8000
0
20000000
40000000
60000000
80000000
Nuclear Genome Position
Coverage
0
500
1500
0
20000
40000
60000
80000
100000
120000
140000
Chloroplast Position
LTPE446
Coverage
0
4000
8000
0
20000000
40000000
60000000
80000000
Nuclear Genome Position
Coverage
0
400
800
1200
0
20000
40000
60000
80000
100000
120000
140000
Chloroplast Position
LTPE447
Coverage
0
400000
1000000
0
20000000
40000000
60000000
80000000
Nuclear Genome Position
Coverage
0
400
800
0
20000
40000
60000
80000
100000
120000
140000
Chloroplast Position

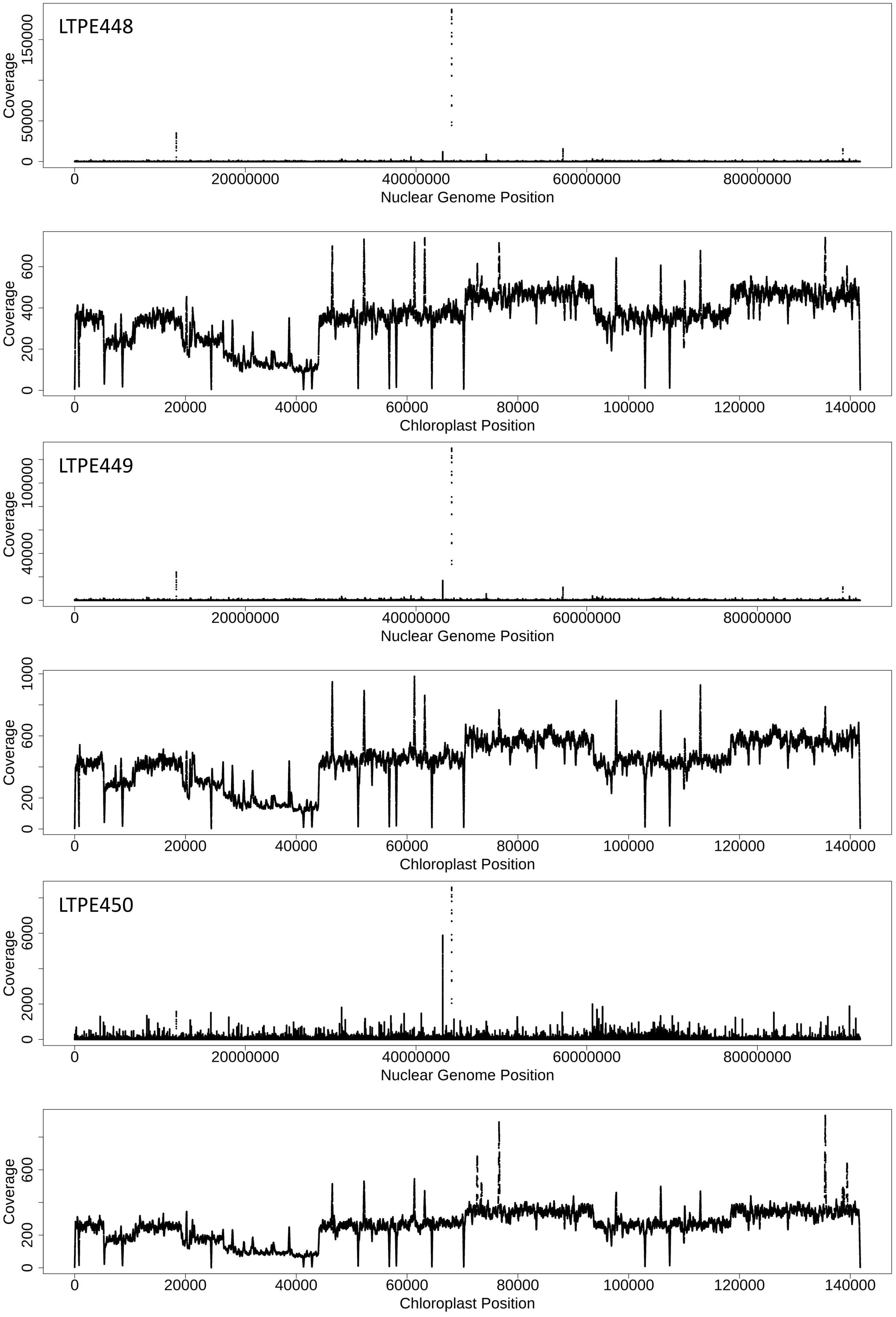
LTPE448
Coverage
0
50000
150000
0
20000000
40000000
60000000
80000000
Nuclear Genome Position
Coverage
0
200
400
600
0
20000
40000
60000
80000
100000
120000
140000
Chloroplast Position
LTPE449
Coverage
0
40000
100000
0
20000000
40000000
60000000
80000000
Nuclear Genome Position
Coverage
0
200
600
1000
0
20000
40000
60000
80000
100000
120000
140000
Chloroplast Position
LTPE450
Coverage
0
2000
6000
0
20000000
40000000
60000000
80000000
Nuclear Genome Position
Coverage
0
200
600
0
20000
40000
60000
80000
100000
120000
140000
Chloroplast Position

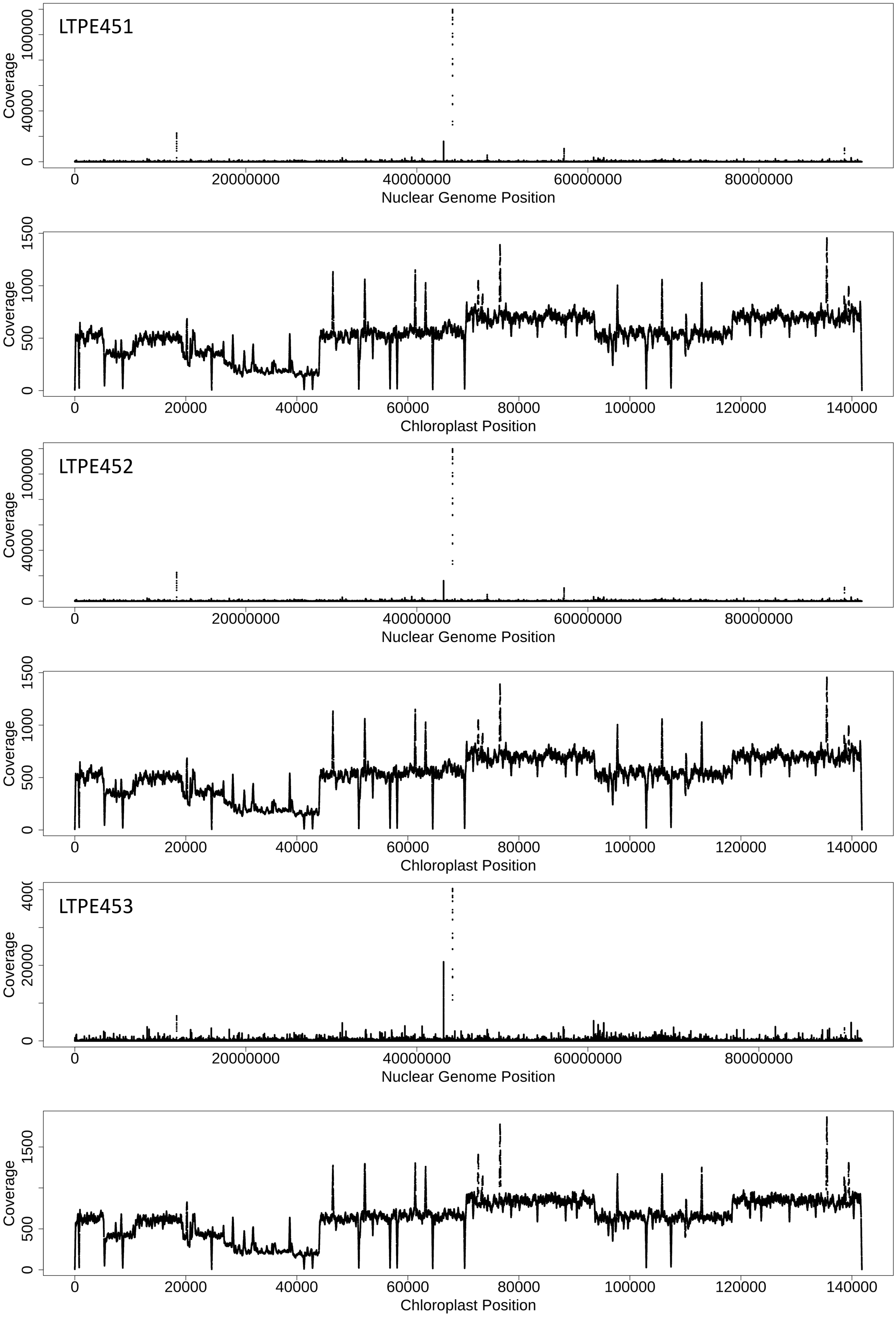
LTPE451
Coverage
0
40000
100000
0
20000000
40000000
60000000
80000000
Nuclear Genome Position
Coverage
0
500
1000
1500
0
20000
40000
60000
80000
100000
120000
140000
Chloroplast Position
LTPE452
Coverage
0
40000
100000
0
20000000
40000000
60000000
80000000
Nuclear Genome Position
Coverage
0
500
1000
1500
0
20000
40000
60000
80000
100000
120000
140000
Chloroplast Position
LTPE453
Coverage
0
20000
0
20000000
40000000
60000000
80000000
Nuclear Genome Position
Coverage
0
500
1500
0
20000
40000
60000
80000
100000
120000
140000
Chloroplast Position

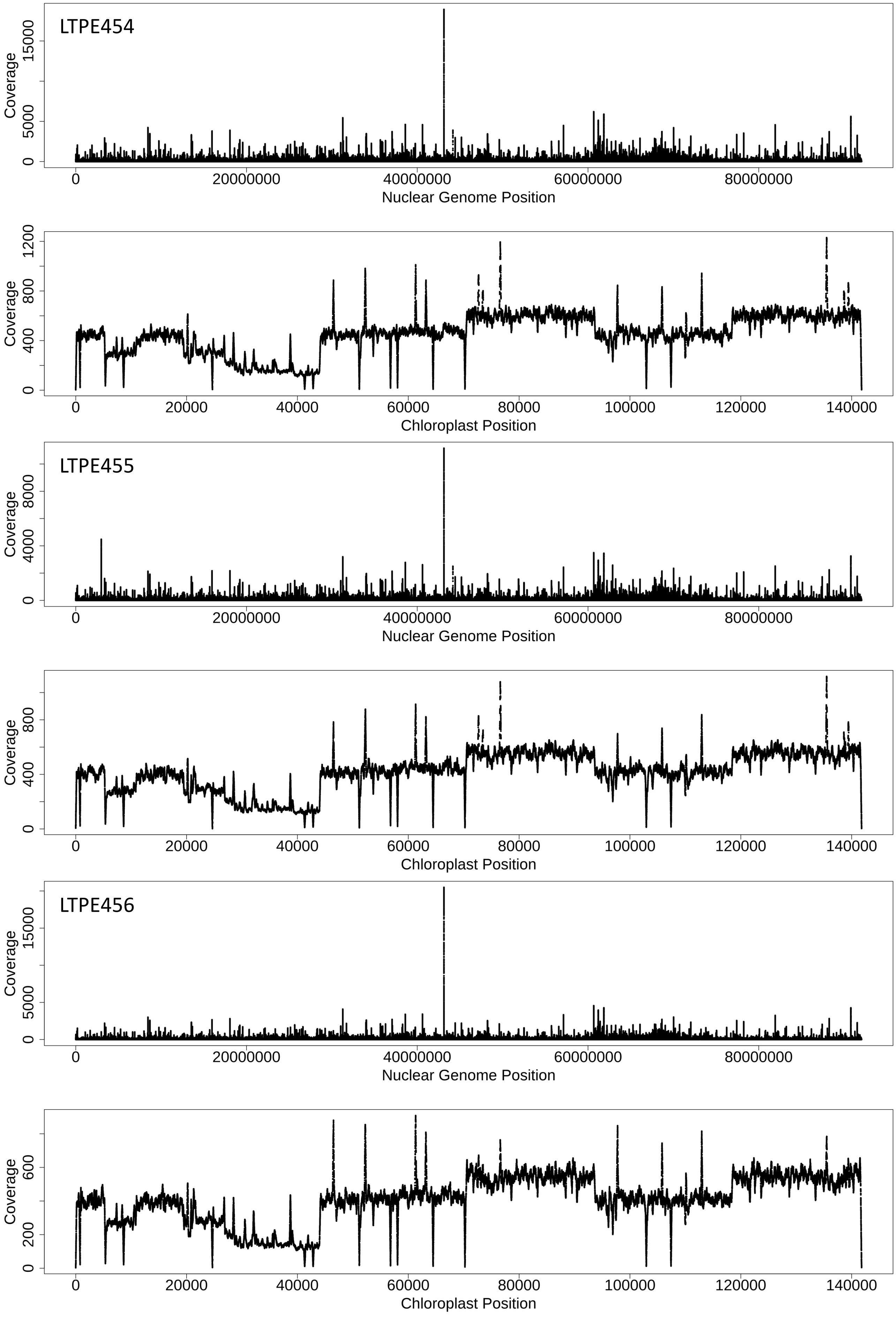
LTPE454
Coverage
0
5000
15000
0
20000000
40000000
60000000
80000000
Nuclear Genome Position
Coverage
0
400
800
1200
0
20000
40000
60000
80000
100000
120000
140000
Chloroplast Position
LTPE455
Coverage
0
4000
8000
Nuclear Genome Position
Coverage
Chloroplast Position
LTPE456
Coverage
Nuclear Genome Position
Coverage
0
200
600
Chloroplast Position

Figure S3. Sequencing coverage for evolved *Thalassiosira oceanica* CCMP1005 genomes. Strains LTPE445 to LTPE450 were evolved at 400 ppm pCO_2; strains LTPE451 to LTPE456 were evolved at 800 ppm pCO_2. For each strain, the nuclear genome and chloroplast genome are shown separately. The CCMP1005 reference genome was a collection of contigs, all of which were concatenated linearly in order to create this plot.

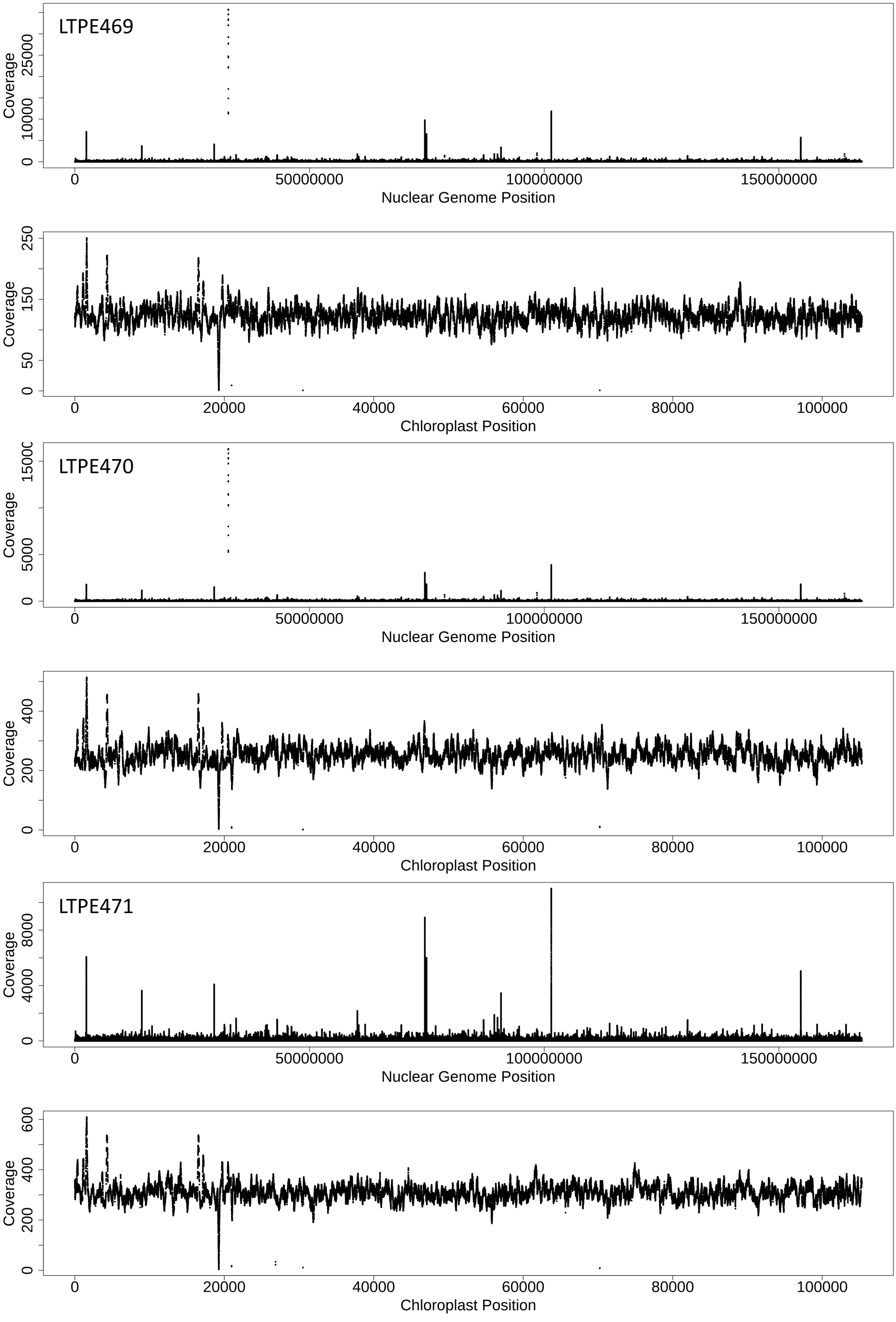

LTPE469
Coverage
0
10000
25000
0
50000000
100000000
150000000
Nuclear Genome Position
Coverage
0
50
150
250
0
20000
40000
60000
80000
100000
Chloroplast Position
LTPE470
Coverage
0
5000
1500
0
50000000
100000000
150000000
Nuclear Genome Position
Coverage
0
200
400
0
20000
40000
60000
80000
100000
Chloroplast Position
LTPE471
Coverage
0
4000
8000
0
50000000
100000000
150000000
Nuclear Genome Position
Coverage
0
200
400
600
0
20000
40000
60000
80000
100000
Chloroplast Position

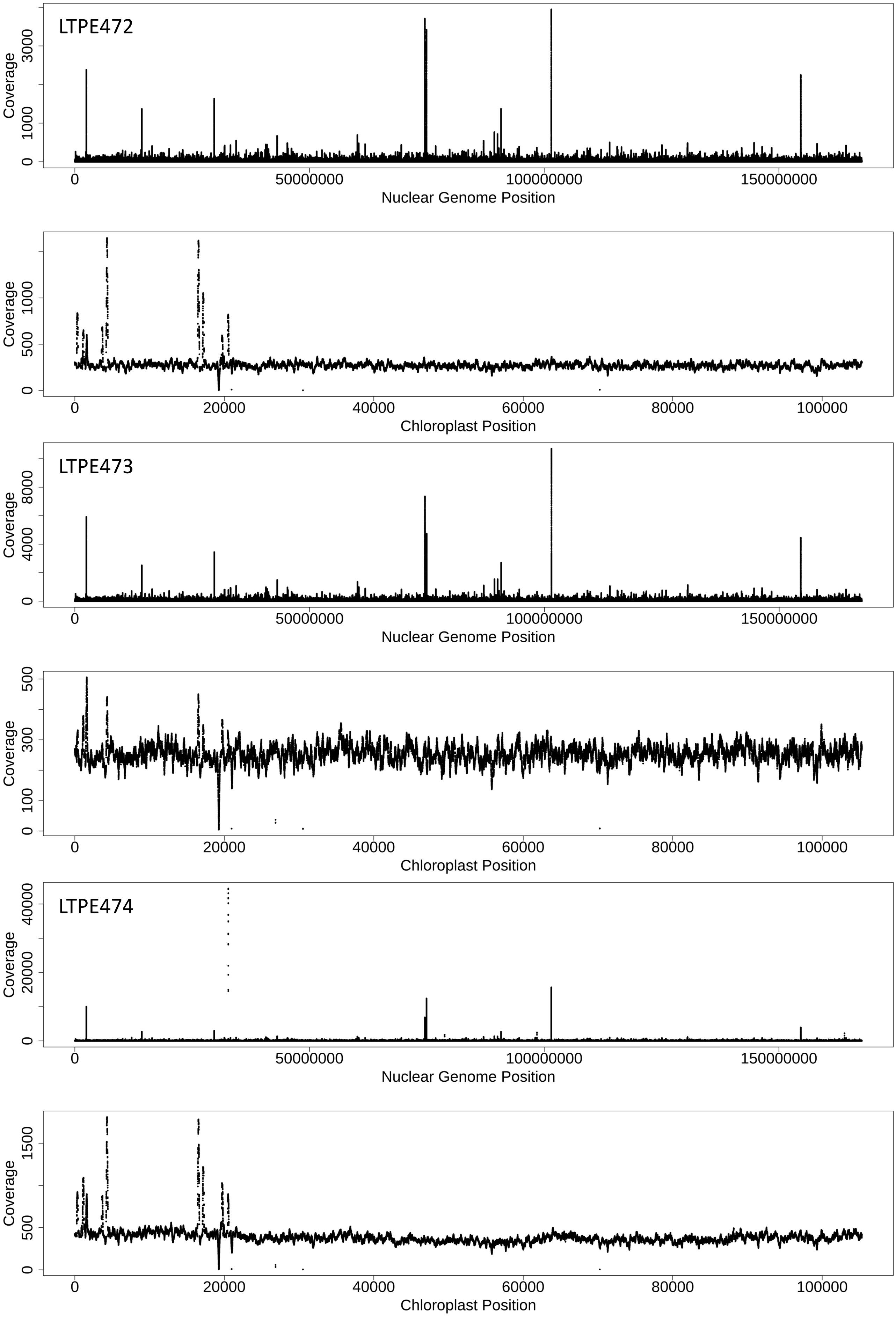

LTPE472
Coverage
0
1000
3000
0
50000000
100000000
150000000
Nuclear Genome Position
Coverage
0
500
1000
0
20000
40000
60000
80000
100000
Chloroplast Position
LTPE473
Coverage
0
4000
8000
0
50000000
100000000
150000000
Nuclear Genome Position
Coverage
0
100
300
500
0
20000
40000
60000
80000
100000
Chloroplast Position
LTPE474
Coverage
0
20000
40000
0
50000000
100000000
150000000
Nuclear Genome Position
Coverage
0
500
1500
0
20000
40000
60000
80000
100000
Chloroplast Position

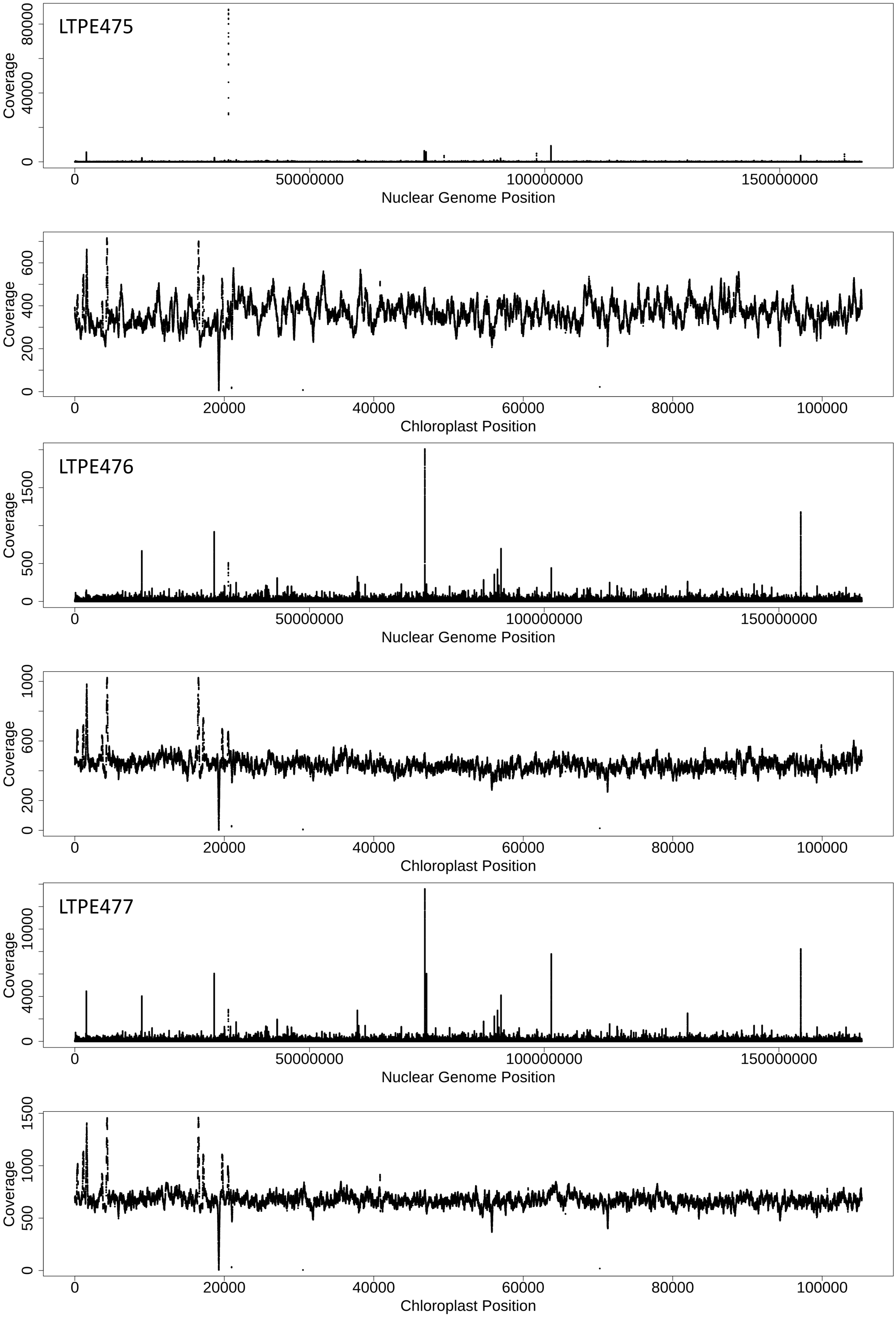
LTPE475
Coverage
0
40000
80000
0
50000000
100000000
150000000
Nuclear Genome Position
Coverage
0
200
400
600
0
20000
40000
60000
80000
100000
Chloroplast Position
LTPE476
Coverage
0
500
1500
0
50000000
100000000
150000000
Nuclear Genome Position
Coverage
0
200
600
1000
0
20000
40000
60000
80000
100000
Chloroplast Position
LTPE477
Coverage
0
4000
10000
0
50000000
100000000
150000000
Nuclear Genome Position
Coverage
0
500
1000
1500
0
20000
40000
60000
80000
100000
Chloroplast Position

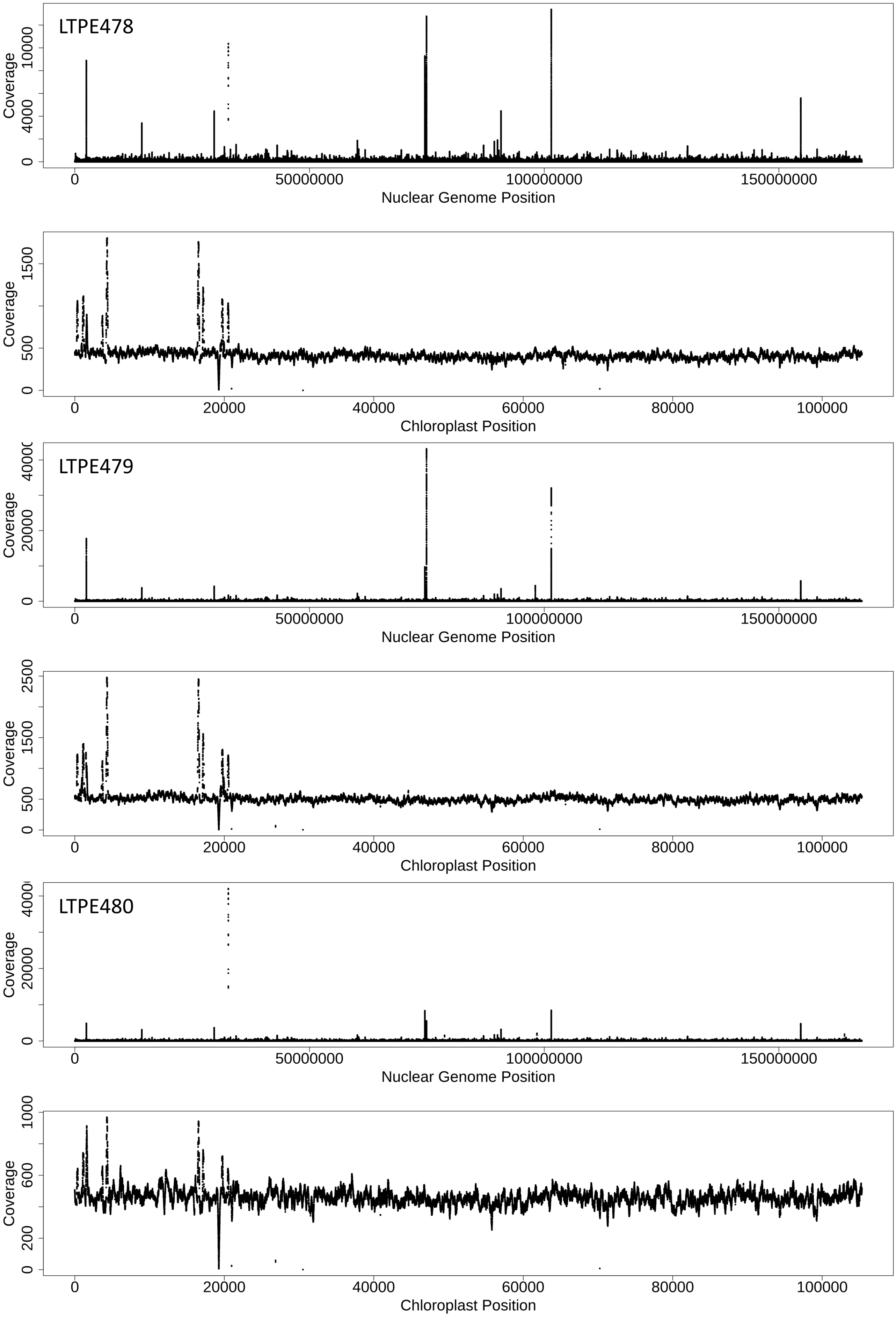
LTPE478
Coverage
Nuclear Genome Position
Coverage
Chloroplast Position
LTPE479
Coverage
Nuclear Genome Position
Coverage
Chloroplast Position
LTPE480
Coverage
Nuclear Genome Position
Coverage
Chloroplast Position

Figure S4. Sequencing coverage for evolved *Emiliania huxleyi* CCMP371 genomes. Strains LTPE469 to LTPE474 were evolved at 400 ppm pCO_2; strains LTPE475 to LTPE480 were evolved at 800 ppm pCO_2. For each strain, the nuclear genome and chloroplast genome are shown separately. The CCMP371 reference genome was a collection of contigs, all of which were concatenated linearly in order to create this plot.

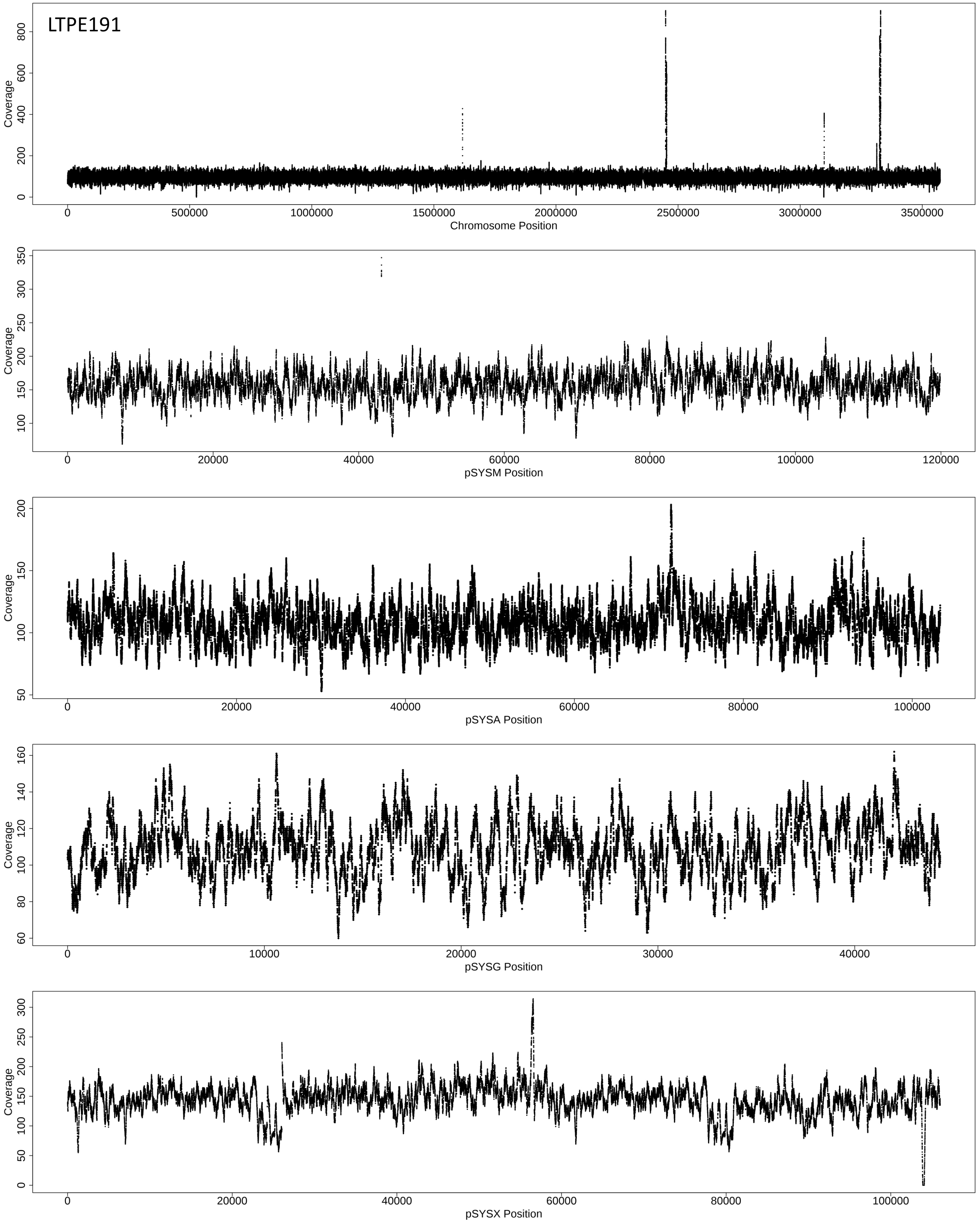

LTPE191
Coverage
Chromosome Position
0
500000
1000000
1500000
2000000
2500000
3000000
3500000
0
200
400
600
800
pSYSM Position
0
20000
40000
60000
80000
100000
120000
100
150
200
250
300
350
pSYSA Position
50
100
150
200
pSYSG Position
10000
30000
40000
60
80
120
140
160
pSYSX Position
50

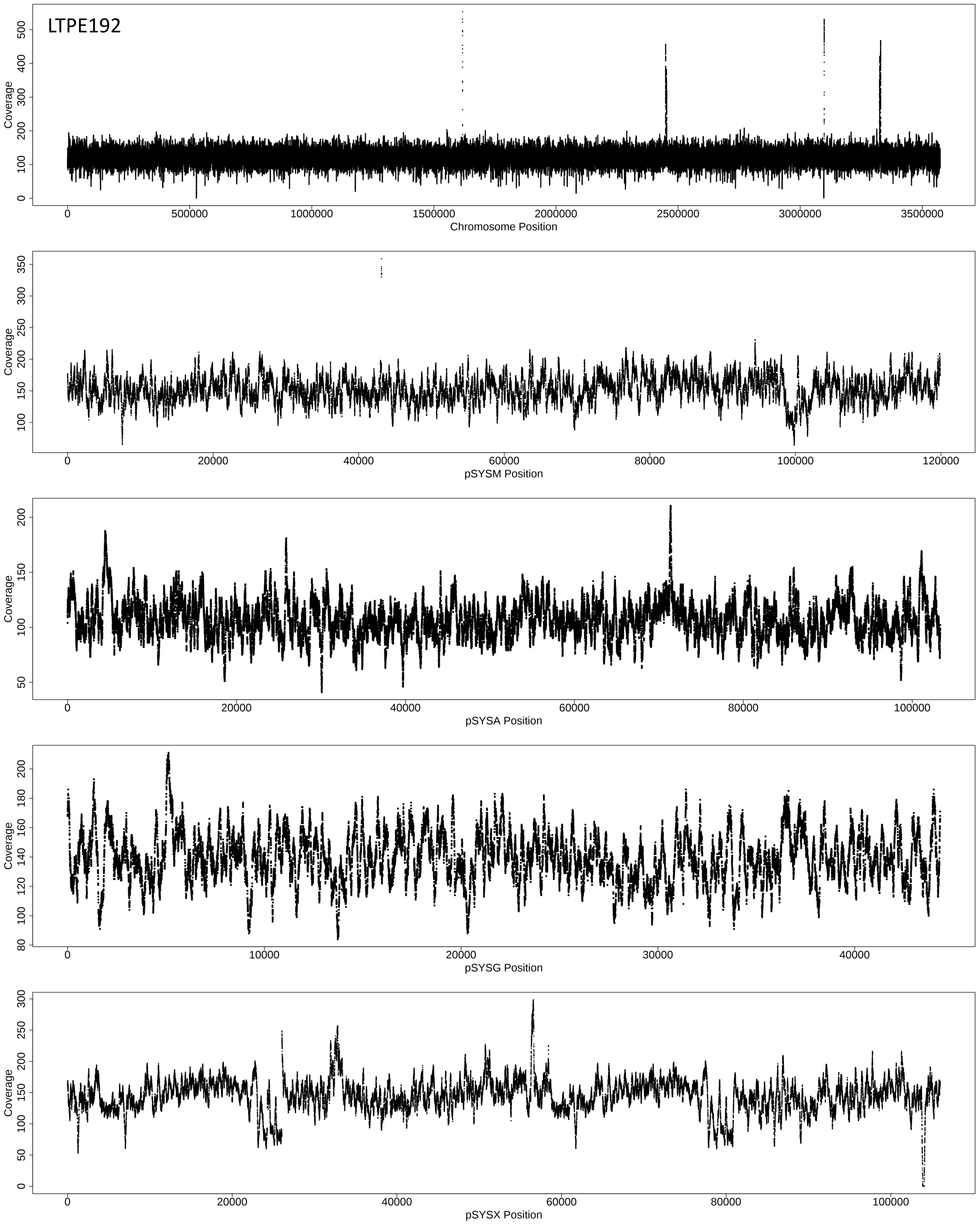

LTPE192
Coverage
Chromosome Position
pSYSM Position
pSYSA Position
pSYSG Position
pSYSX Position

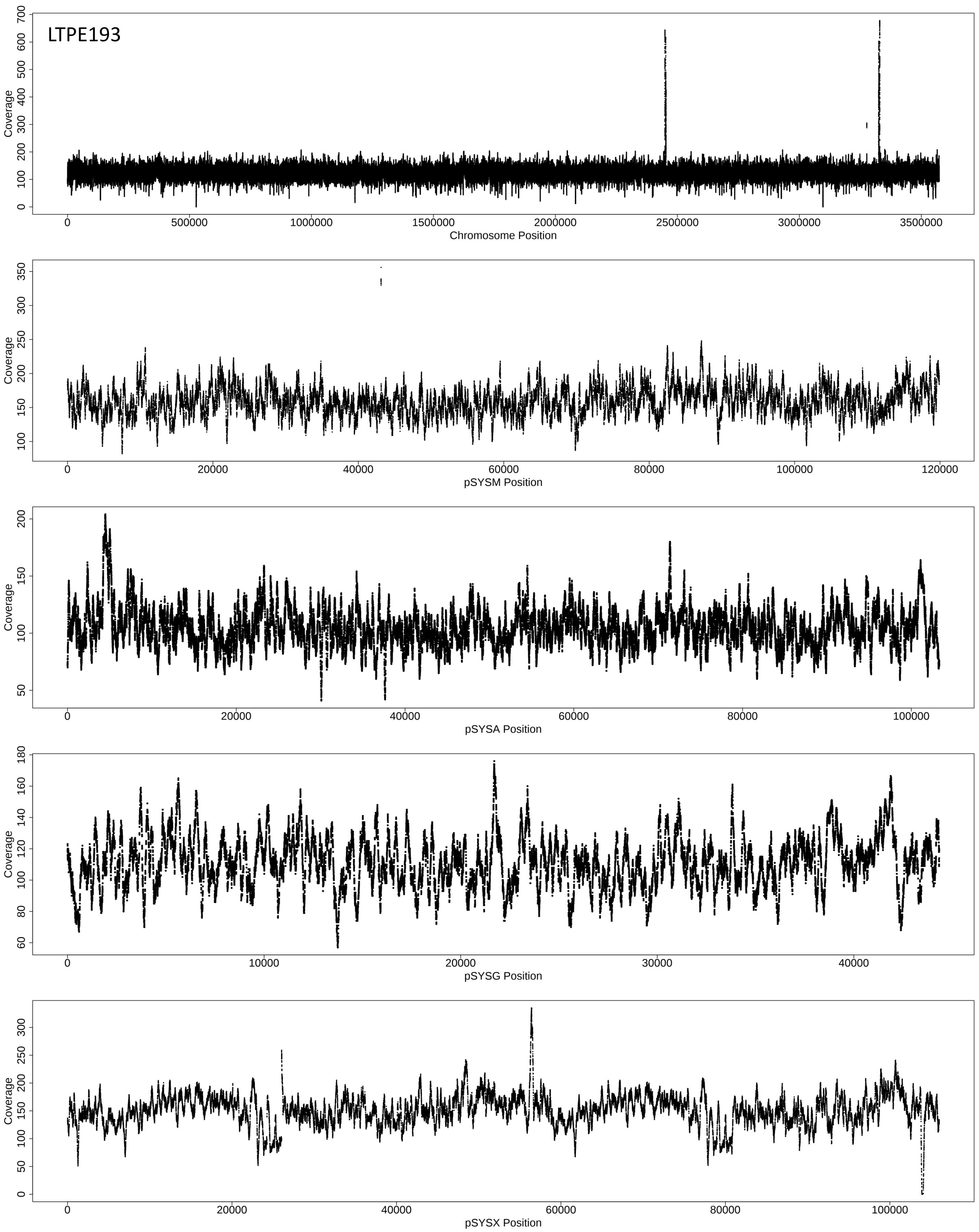
LTPE193
Coverage
Chromosome Position
0
500000
1000000
1500000
2000000
2500000
3000000
3500000
0
100
200
300
400
500
600
700
pSYSM Position
0
20000
40000
60000
80000
100000
120000
100
150
200
250
300
350
pSYSA Position
0
20000
40000
60000
80000
100000
50
100
150
200
pSYSG Position
0
10000
20000
30000
40000
60
80
100
120
140
160
180
pSYSX Position
0
20000
40000
60000
80000
100000
0
50
100
150
200
250
300

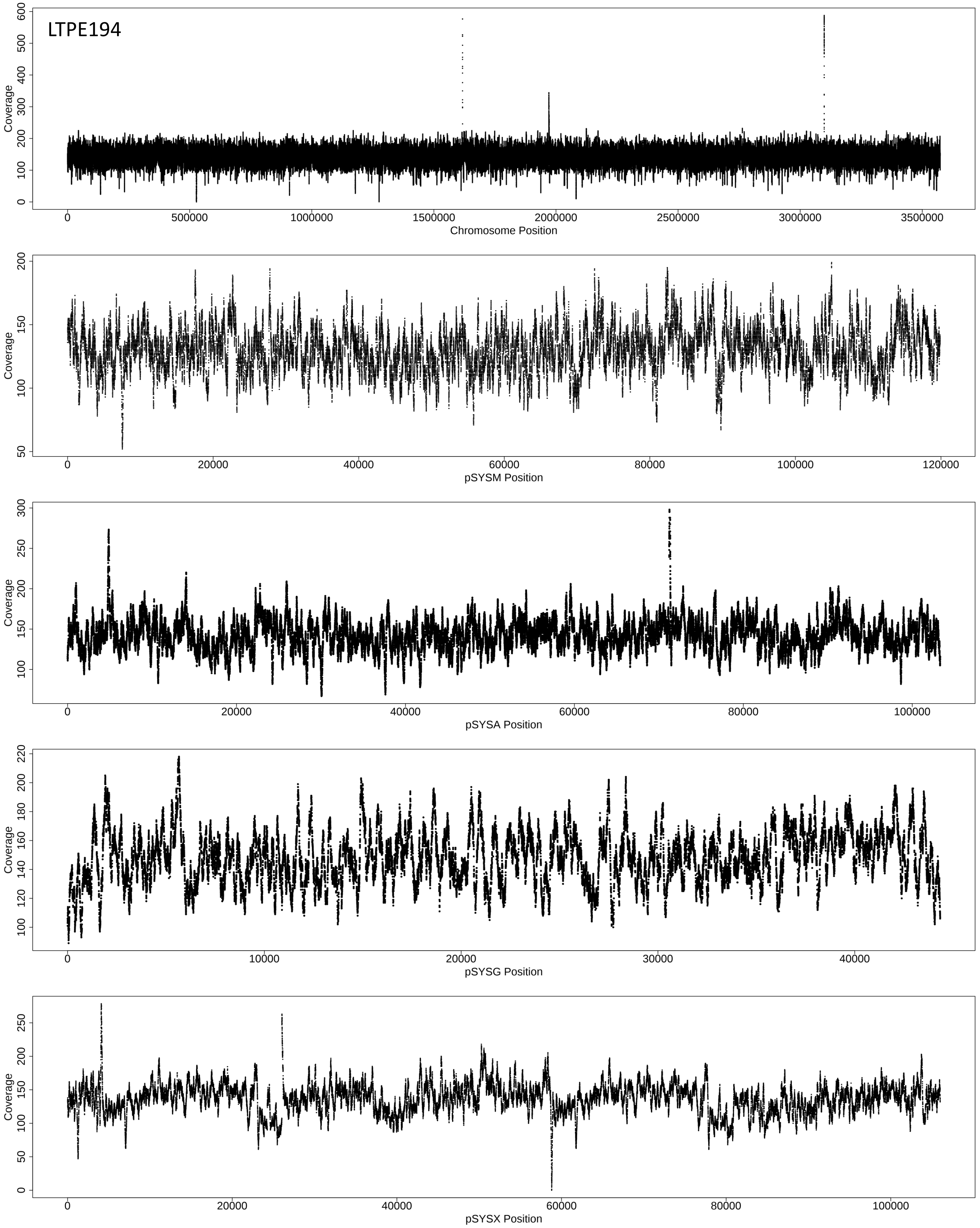

LTPE194
Coverage
Chromosome Position
pSYSM Position
pSYSA Position
pSYSG Position
pSYSX Position

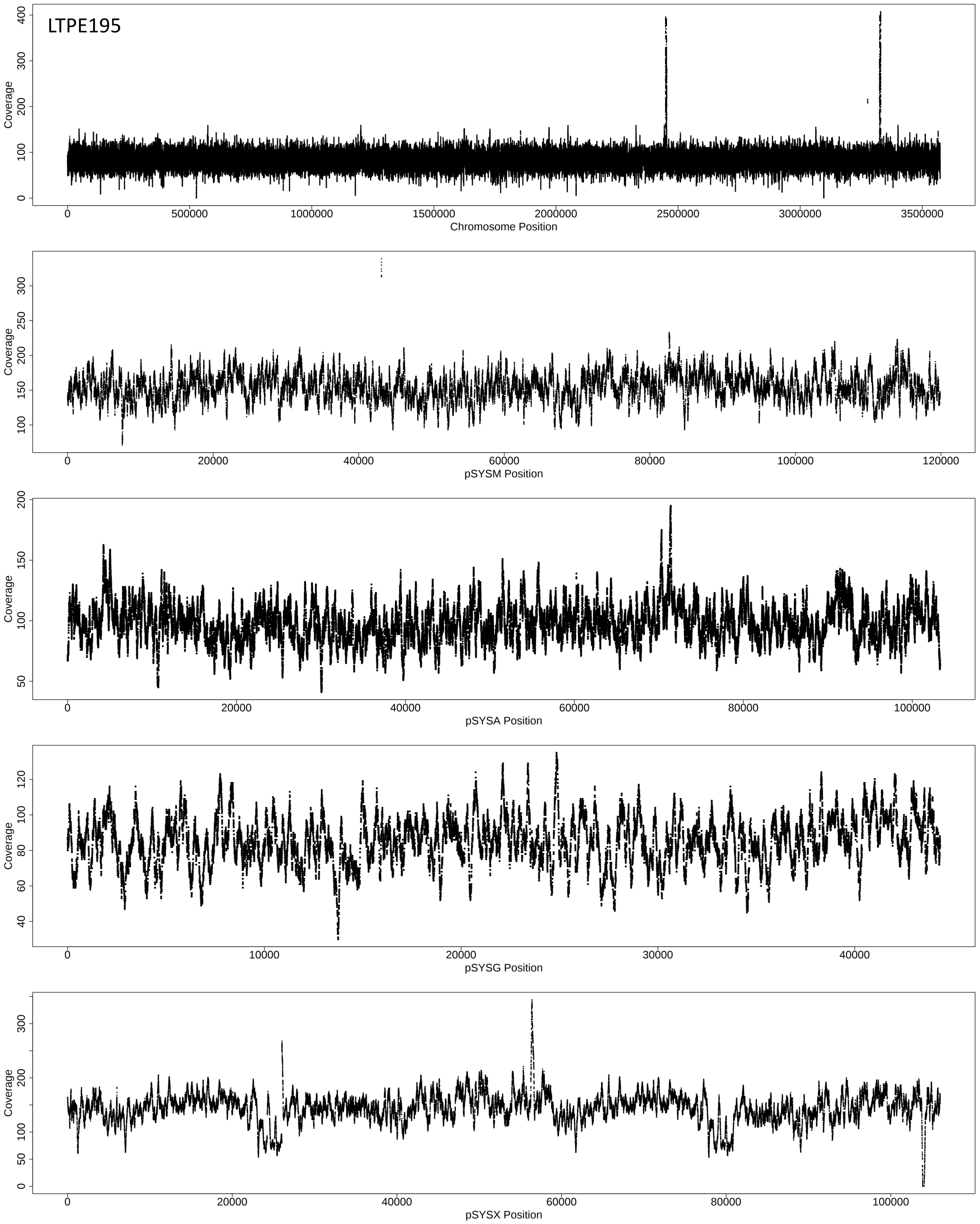
LTPE195
Coverage
0 100 200 300 400
0 500000 1000000 1500000 2000000 2500000 3000000 3500000
Chromosome Position
Coverage
100 150 200 250 300
0 20000 40000 60000 80000 100000 120000
pSYSM Position
Coverage
50 100 150 200
0 20000 40000 60000 80000 100000
pSYSA Position
Coverage
40 60 80 100 120
0 10000 20000 30000 40000
pSYSG Position
Coverage
0 50 100 200 300
0 20000 40000 60000 80000 100000
pSYSX Position

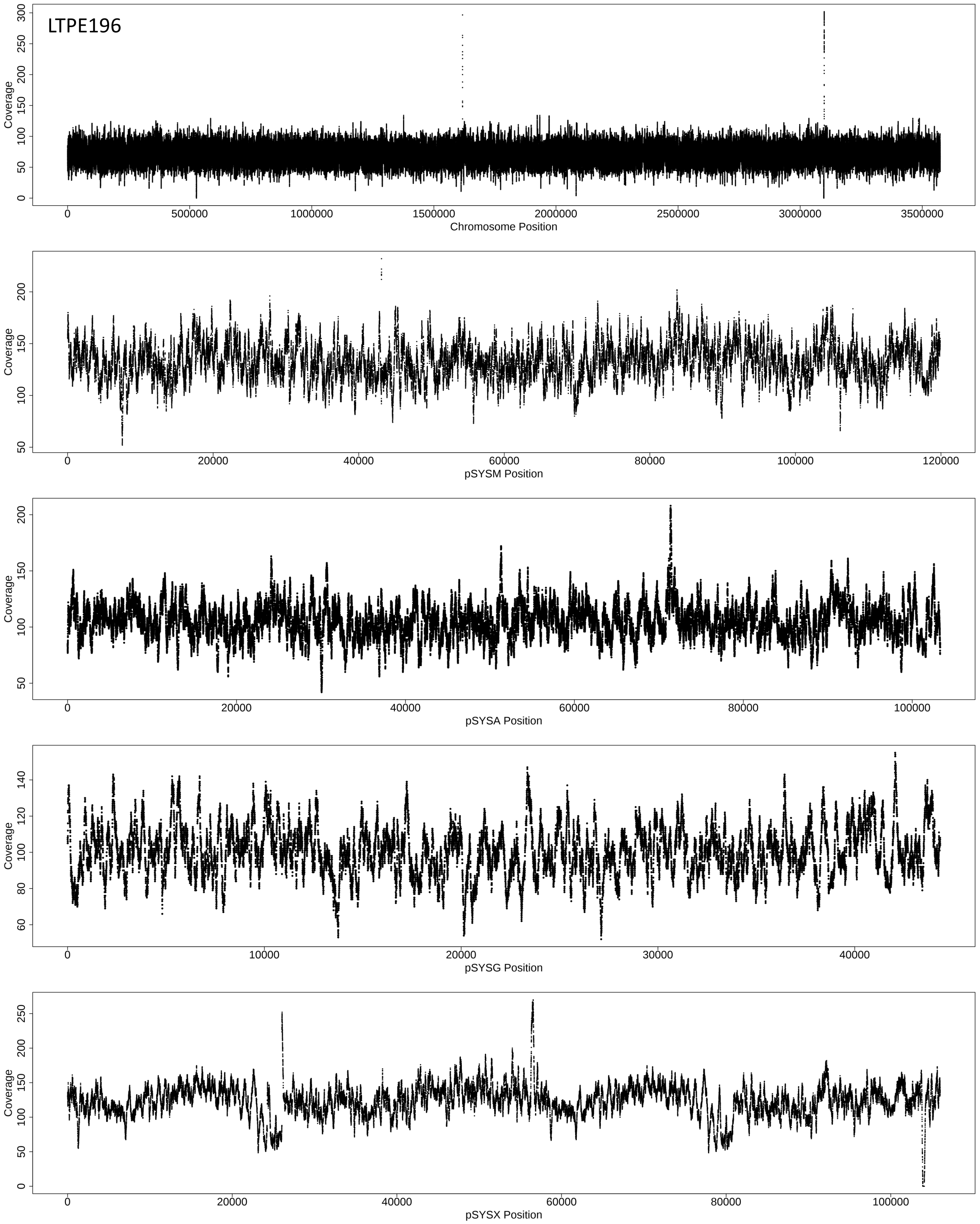
LTPE196
Coverage
Chromosome Position
Coverage
pSYSM Position
Coverage
pSYSA Position
Coverage
pSYSG Position
Coverage
pSYSX Position

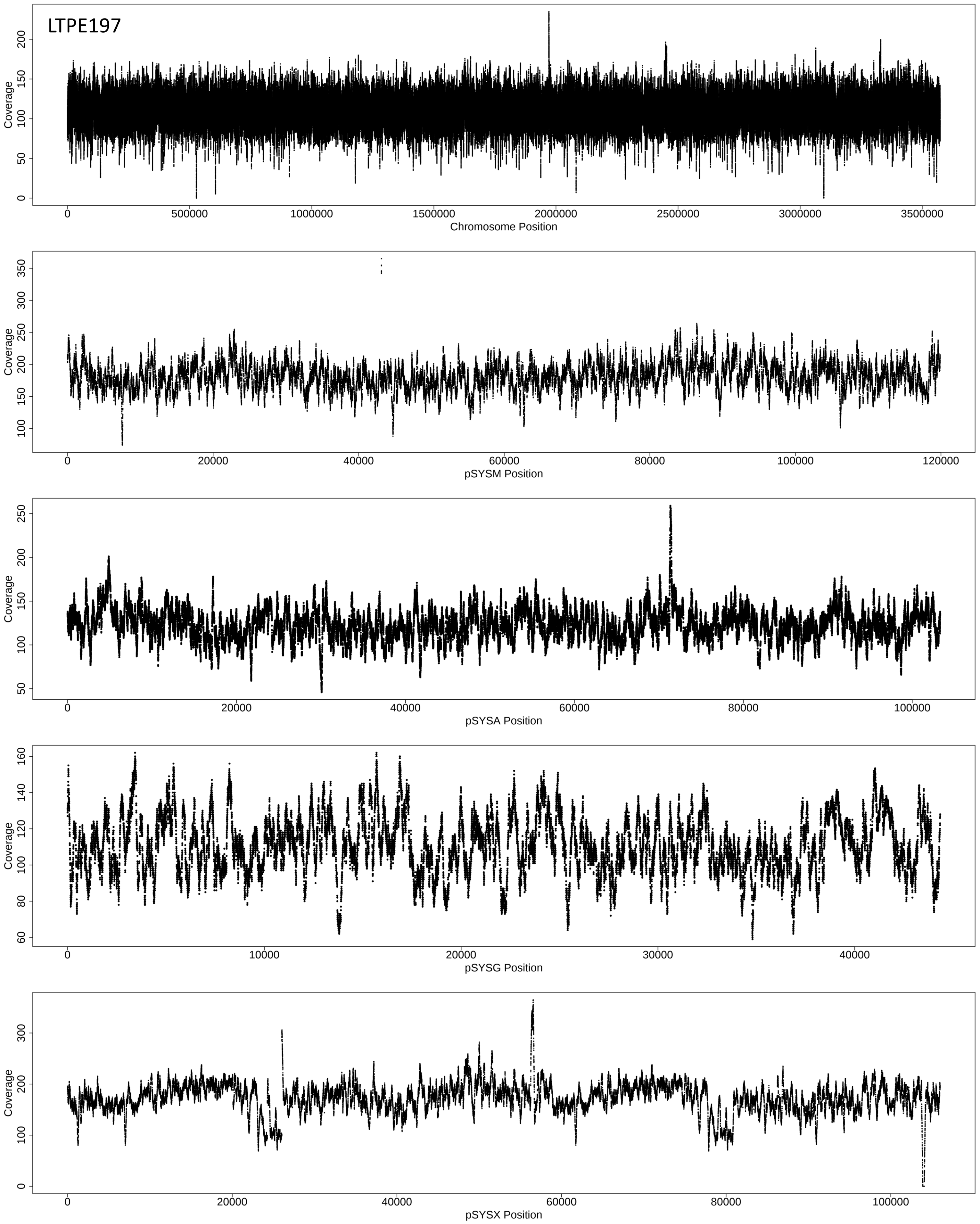
LTPE197
Coverage
Chromosome Position
0
50
100
150
200
500000
1000000
1500000
2000000
2500000
3000000
3500000
pSYSM Position
250
300
350
20000
40000
60000
80000
100000
120000
pSYSA Position
pSYSG Position
60
80
120
140
160
10000
30000
pSYSX Position

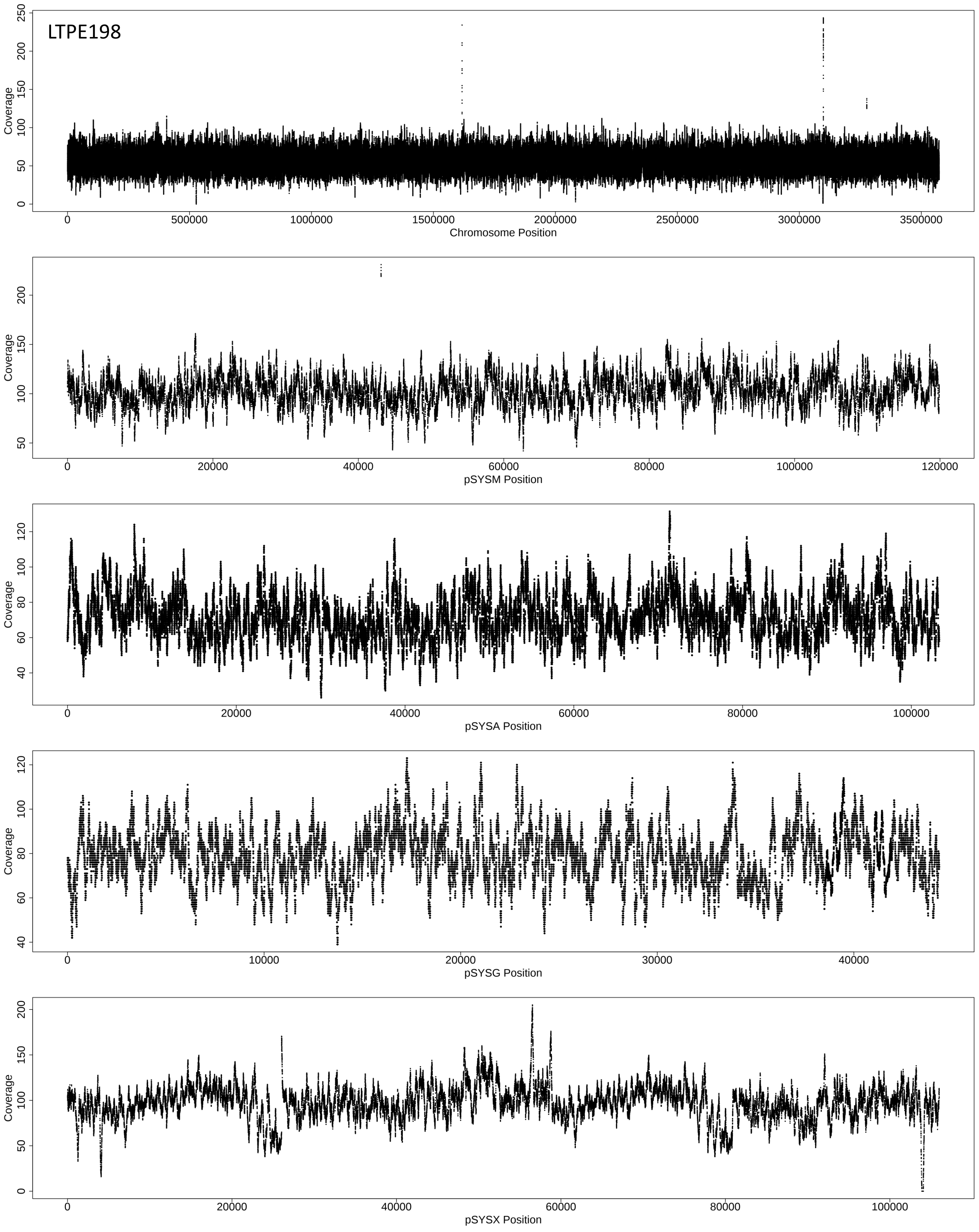
LTPE198
Coverage
Chromosome Position
pSYSM Position
pSYSA Position
pSYSG Position
pSYSX Position

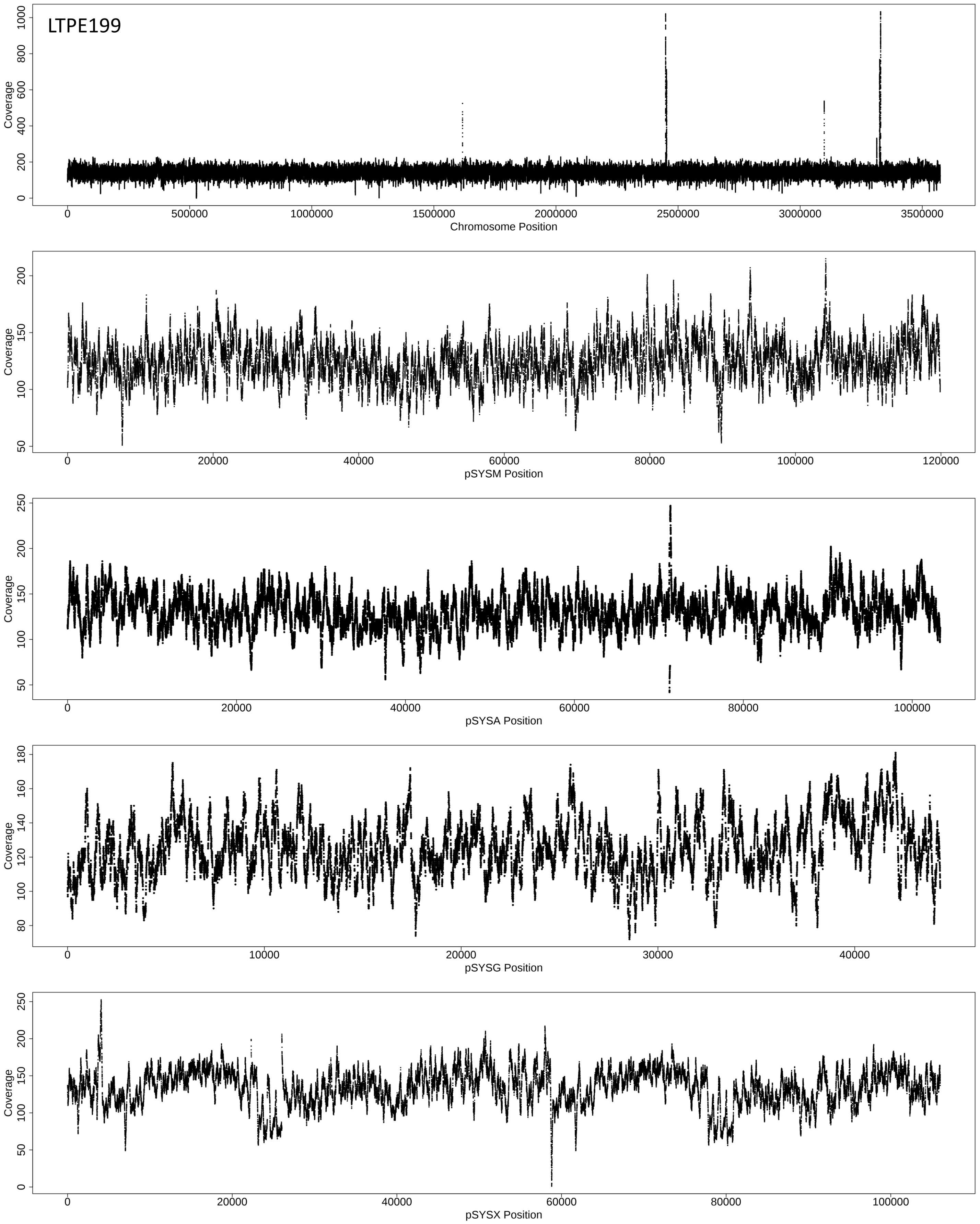
LTPE199
Coverage
0
200
400
600
800
1000
0
500000
1000000
1500000
2000000
2500000
3000000
3500000
Chromosome Position
Coverage
50
100
150
200
0
20000
40000
60000
80000
100000
120000
pSYSM Position
Coverage
50
100
150
200
250
0
20000
40000
60000
80000
100000
pSYSA Position
Coverage
80
100
120
140
160
180
0
10000
20000
30000
40000
pSYSG Position
Coverage
0
50
100
150
200
250
0
20000
40000
60000
80000
100000
pSYSX Position

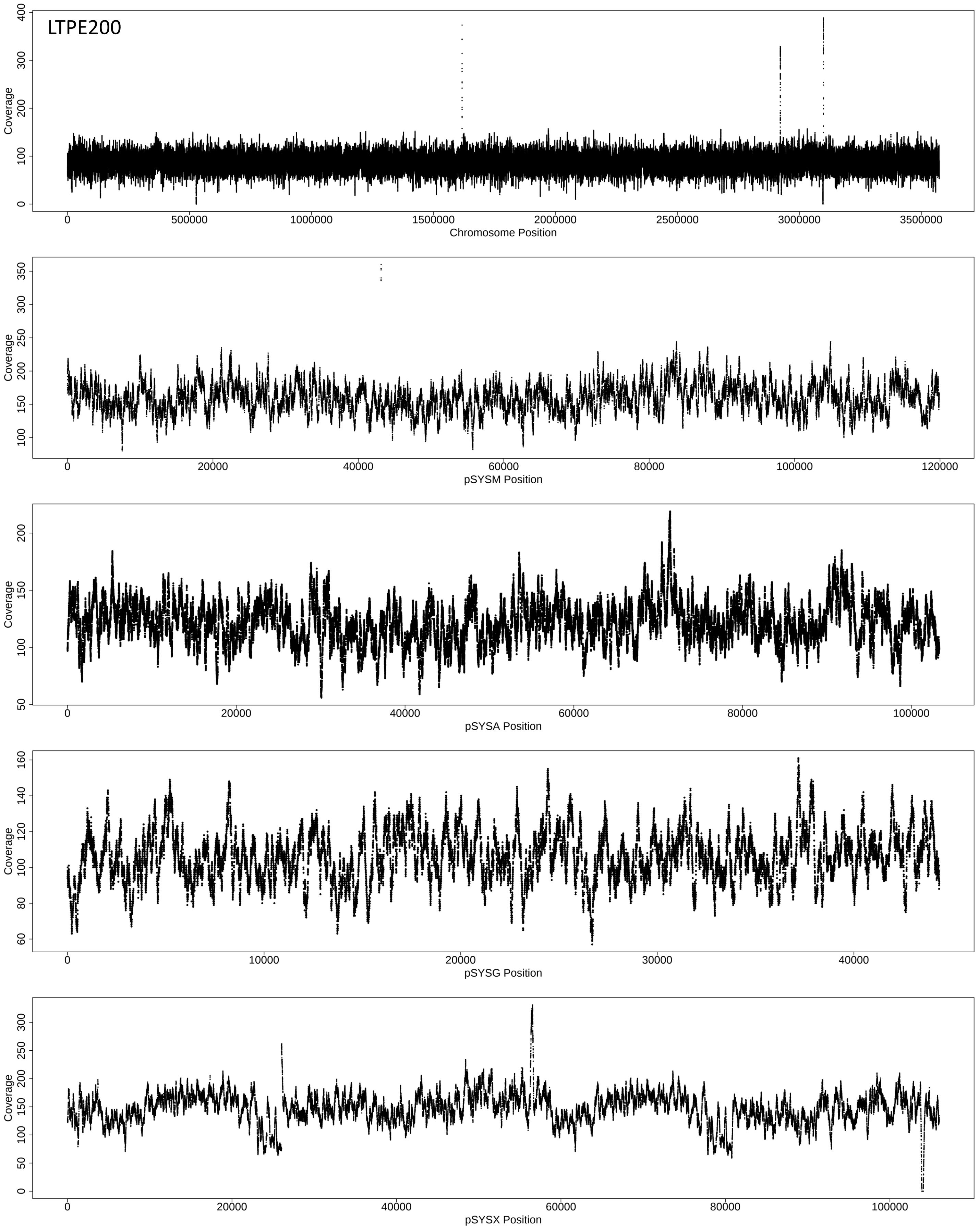
LTPE200
Coverage
0
100
200
300
400
0
500000
1000000
1500000
2000000
2500000
3000000
3500000
Chromosome Position
Coverage
100
150
200
250
300
350
0
20000
40000
60000
80000
100000
120000
pSYSM Position
Coverage
50
100
150
200
0
20000
40000
60000
80000
100000
pSYSA Position
Coverage
60
80
100
120
140
160
0
10000
20000
30000
40000
pSYSG Position
Coverage
0
50
100
150
200
250
300
0
20000
40000
60000
80000
100000
pSYSX Position

Figure S5. Sequencing coverage for evolved *Synechocystis* PCC6803 genomes. Strains LTPE191 to LTPE195 were evolved at 400 ppm pCO_2; strains LTPE196 to LTPE200 were evolved at 800 ppm pCO_2. For each strain, the main chromosome and each of the four plasmids from the reference genome assembly are shown separately.

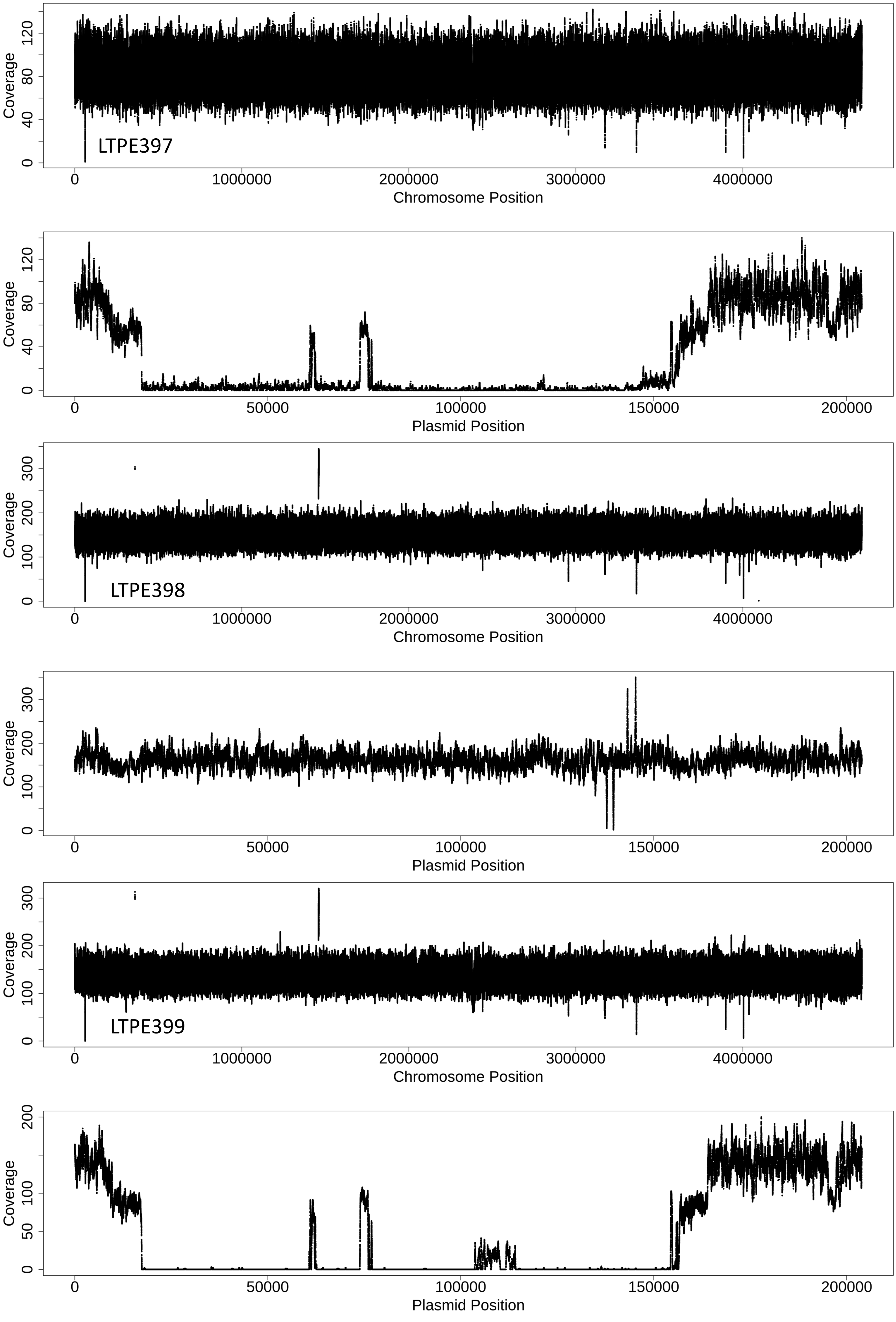

LTPE397
Coverage
0
40
80
120
0
1000000
2000000
3000000
4000000
Chromosome Position
Coverage
0
40
80
120
0
50000
100000
150000
200000
Plasmid Position
LTPE398
Coverage
0
100
200
300
0
1000000
2000000
3000000
4000000
Chromosome Position
Coverage
0
100
200
300
0
50000
100000
150000
200000
Plasmid Position
LTPE399
Coverage
0
100
200
300
0
1000000
2000000
3000000
4000000
Chromosome Position
Coverage
0
50
100
200
0
50000
100000
150000
200000
Plasmid Position

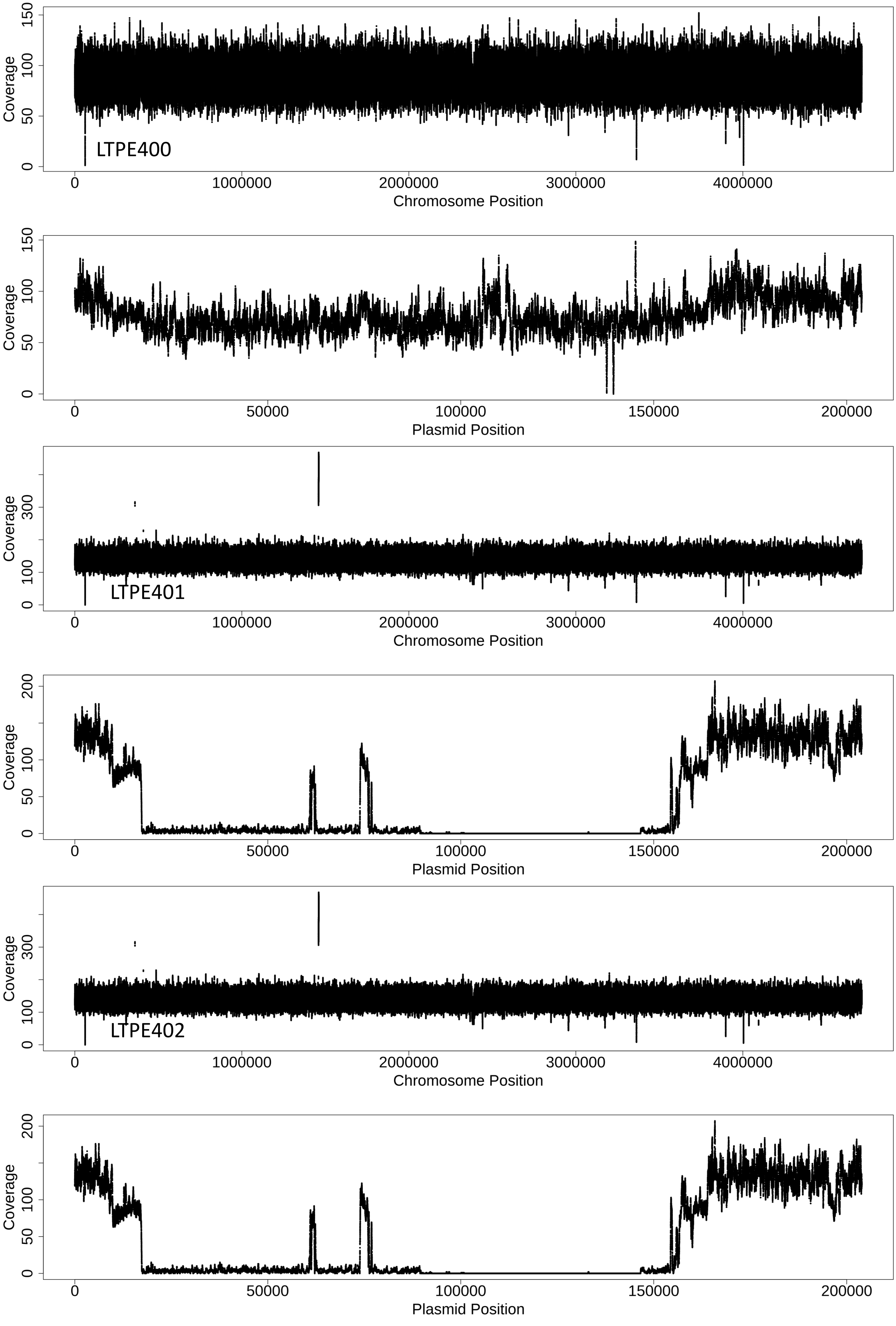
LTPE400
Coverage
0
50
100
150
0
1000000
2000000
3000000
4000000
Chromosome Position
Coverage
0
50
100
150
0
50000
100000
150000
200000
Plasmid Position
LTPE401
Coverage
0
100
300
0
1000000
2000000
3000000
4000000
Chromosome Position
Coverage
0
50
100
200
0
50000
100000
150000
200000
Plasmid Position
LTPE402
Coverage
0
100
300
0
1000000
2000000
3000000
4000000
Chromosome Position
Coverage
0
50
100
200
0
50000
100000
150000
200000
Plasmid Position

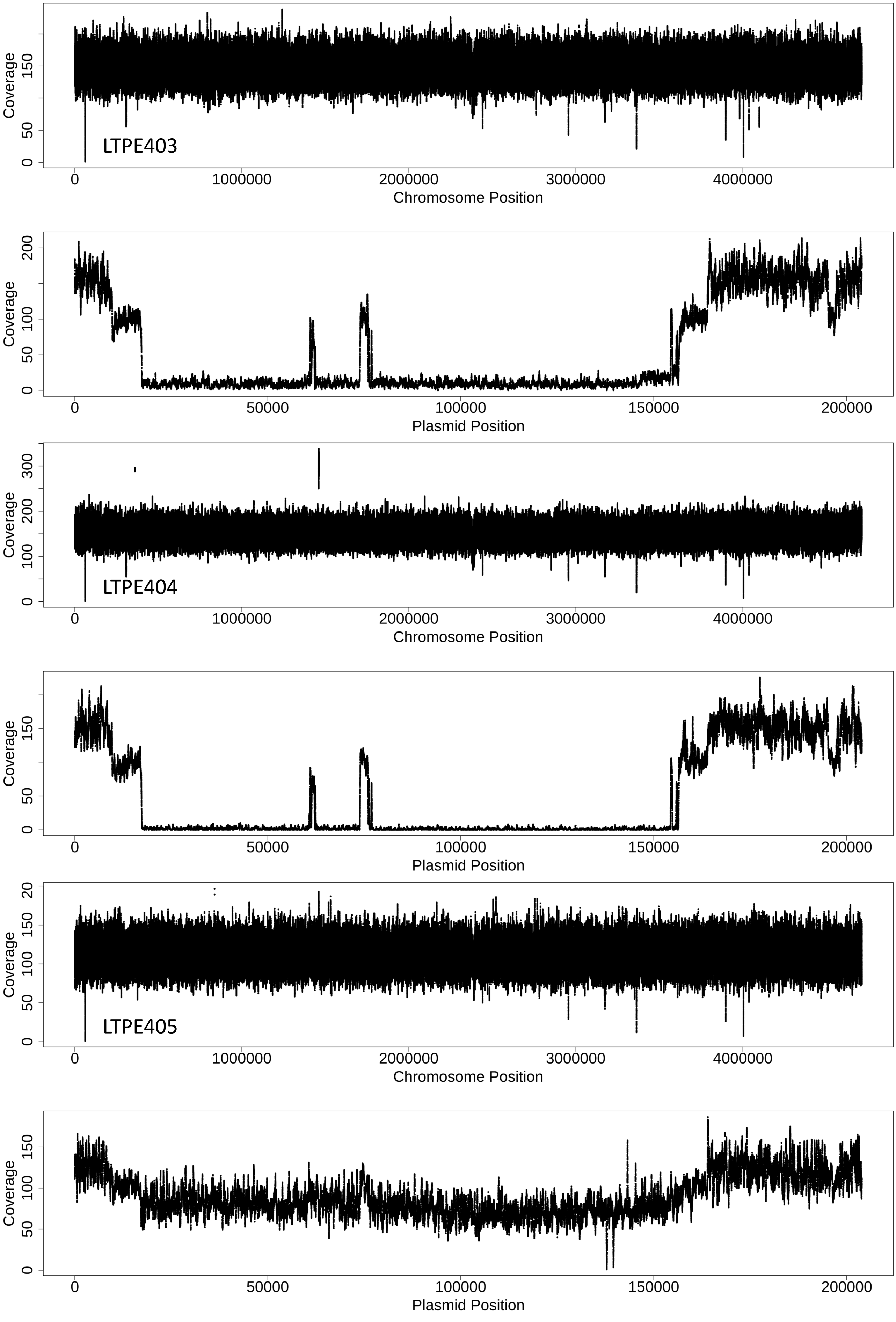
LTPE403
Coverage
0
50
150
0
1000000
2000000
3000000
4000000
Chromosome Position
Coverage
0
50
100
200
0
50000
100000
150000
200000
Plasmid Position
LTPE404
Coverage
0
100
200
300
0
1000000
2000000
3000000
4000000
Chromosome Position
Coverage
0
50
150
0
50000
100000
150000
200000
Plasmid Position
LTPE405
Coverage
0
50
100
150
20
0
1000000
2000000
3000000
4000000
Chromosome Position
Coverage
0
50
100
150
0
50000
100000
150000
200000
Plasmid Position

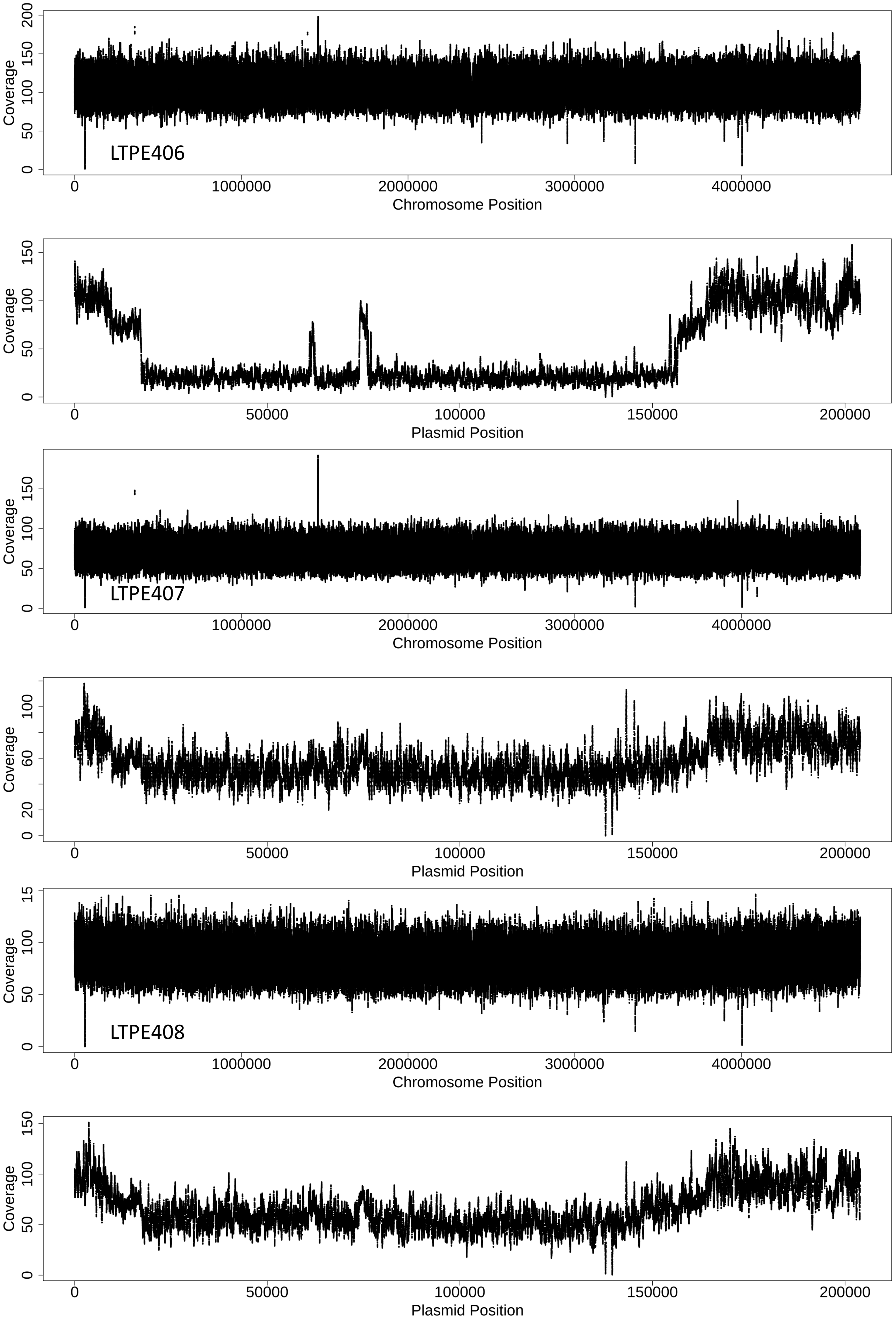
LTPE406
Coverage
Chromosome Position
0
1000000
2000000
3000000
4000000
0
50
100
150
200
Coverage
Plasmid Position
0
50000
100000
150000
200000
LTPE407
Coverage
Chromosome Position
Coverage
Plasmid Position
0
20
60
100
LTPE408
Coverage
Chromosome Position
15
Coverage
Plasmid Position

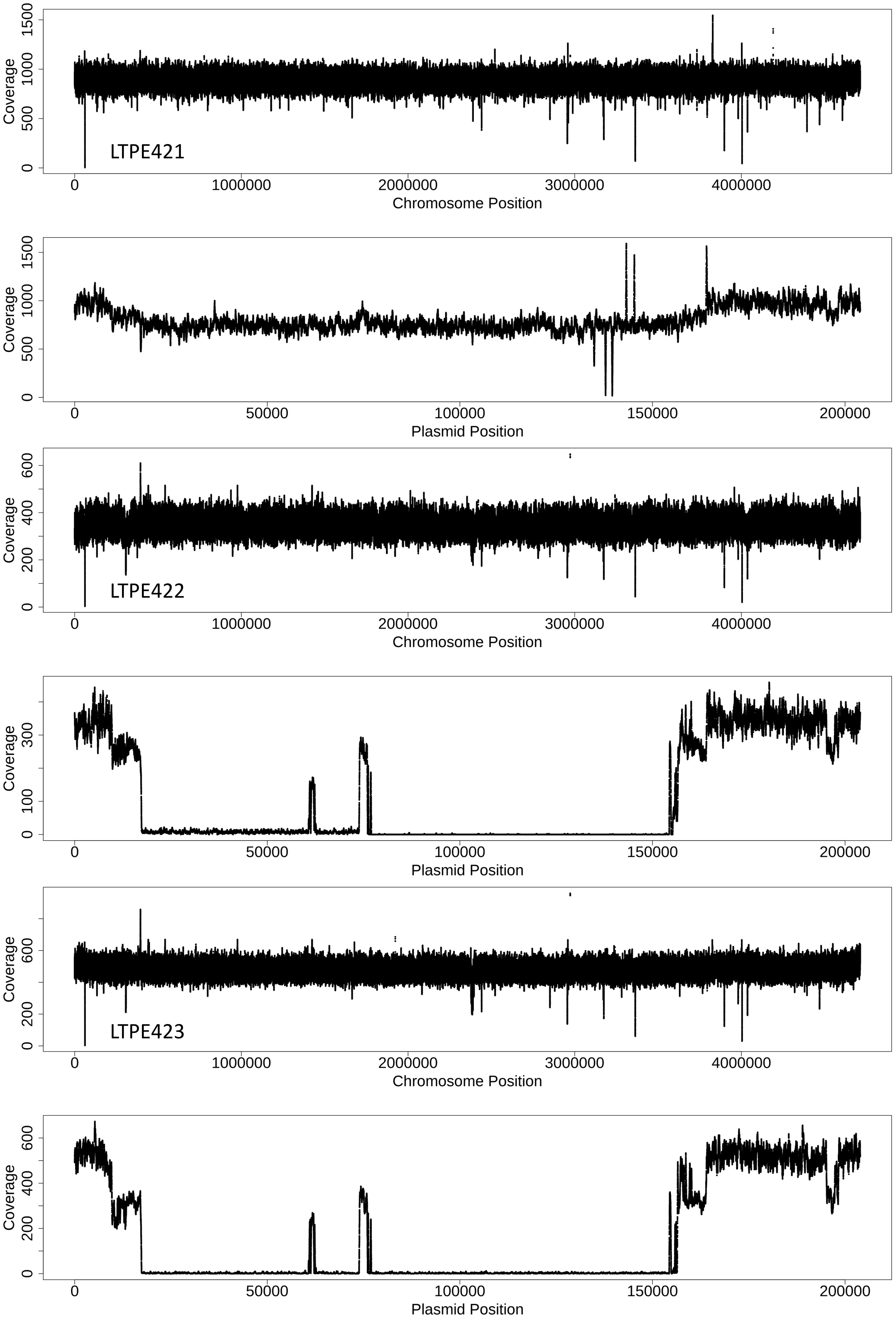
Coverage
0
500
1000
1500
LTPE421
0
1000000
2000000
3000000
4000000
Chromosome Position
Coverage
0
500
1000
1500
0
50000
100000
150000
200000
Plasmid Position
Coverage
0
200
400
600
LTPE422
0
1000000
2000000
3000000
4000000
Chromosome Position
Coverage
0
100
300
0
50000
100000
150000
200000
Plasmid Position
Coverage
0
200
600
LTPE423
0
1000000
2000000
3000000
4000000
Chromosome Position
Coverage
0
200
400
600
0
50000
100000
150000
200000
Plasmid Position

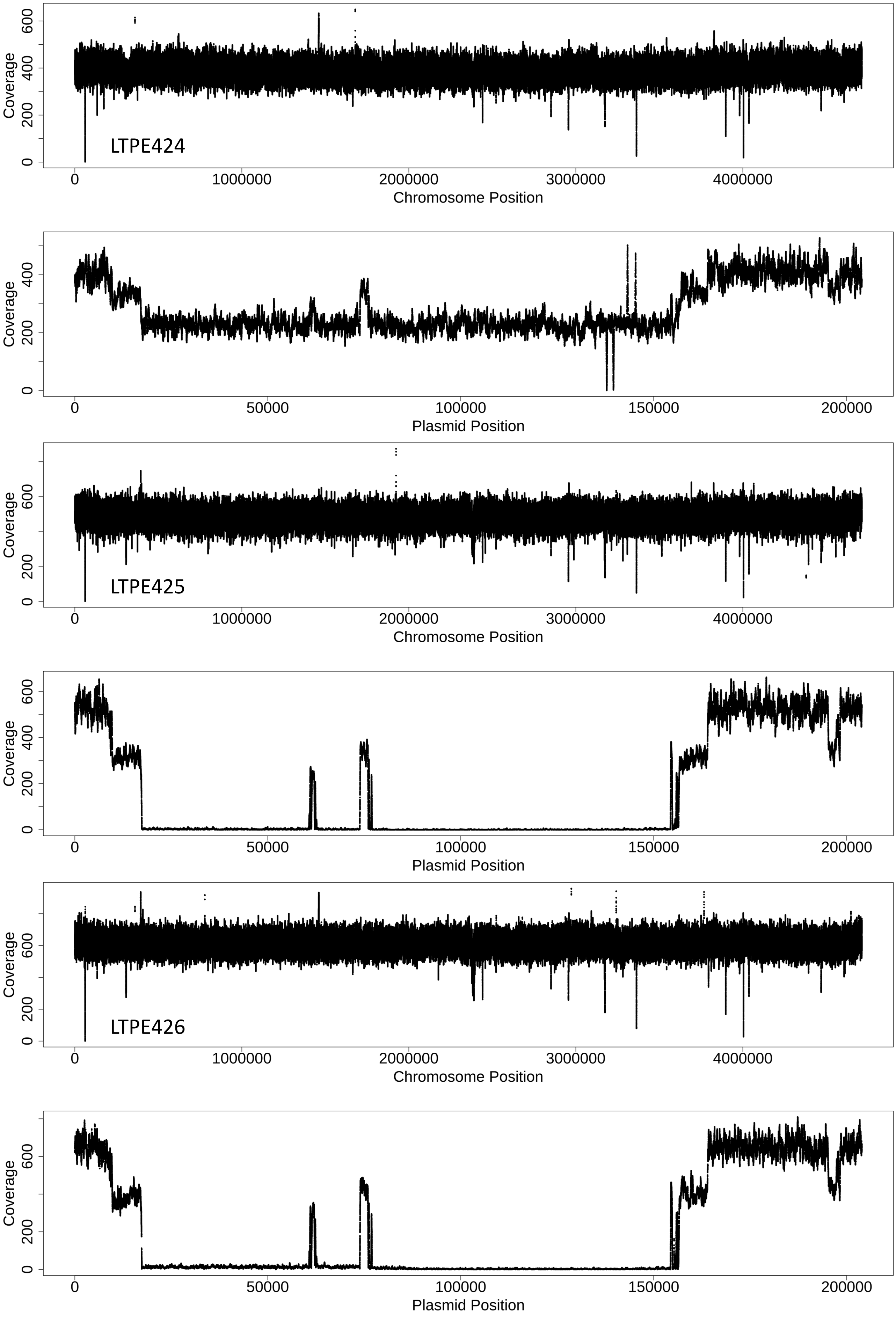

LTPE424
Coverage
0
200
400
600
0
1000000
2000000
3000000
4000000
Chromosome Position
Coverage
0
200
400
0
50000
100000
150000
200000
Plasmid Position
LTPE425
Coverage
0
200
400
600
0
1000000
2000000
3000000
4000000
Chromosome Position
Coverage
0
200
400
600
0
50000
100000
150000
200000
Plasmid Position
LTPE426
Coverage
0
200
400
600
0
1000000
2000000
3000000
4000000
Chromosome Position
Coverage
0
200
400
600
0
50000
100000
150000
200000
Plasmid Position

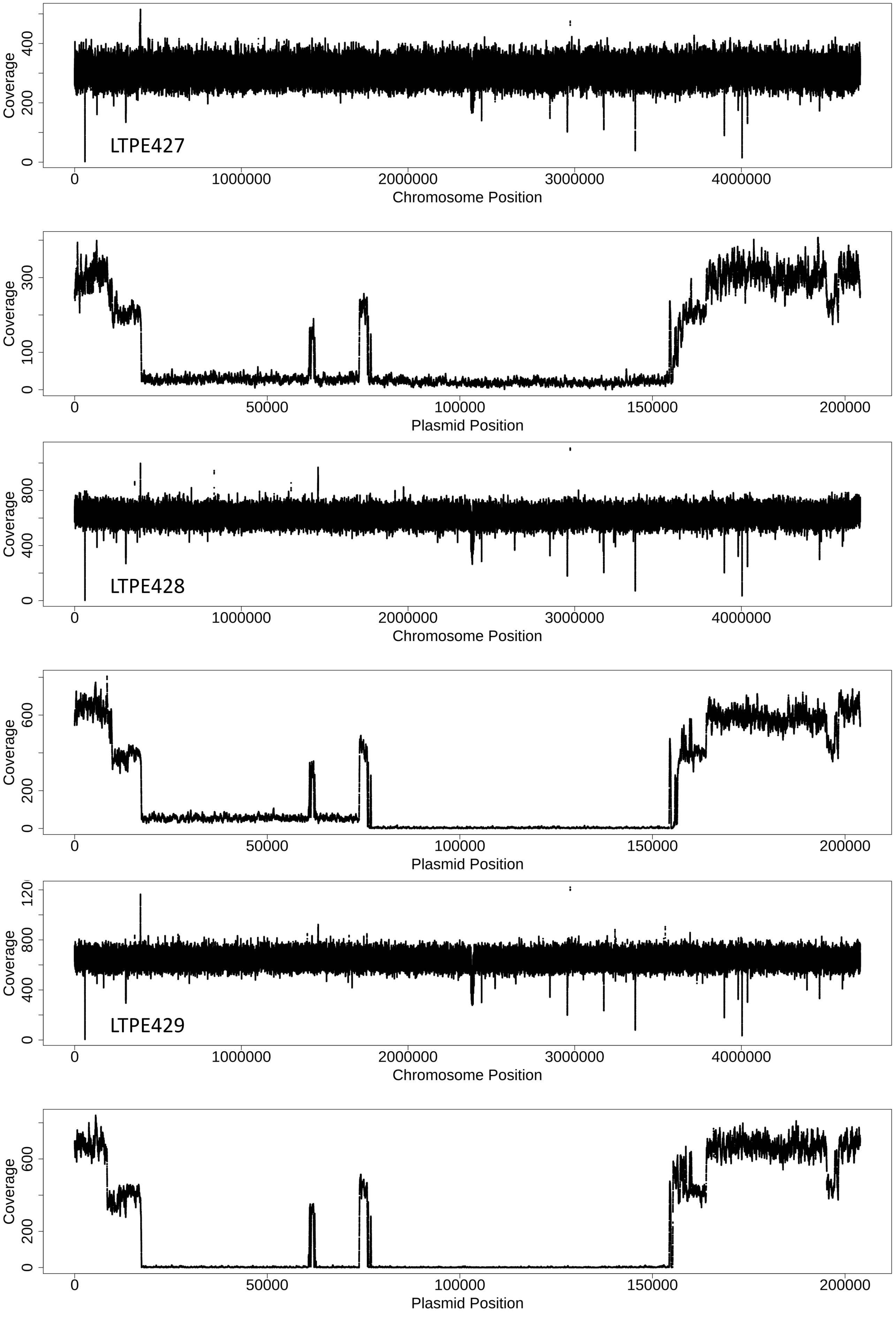

LTPE427
Coverage
0
200
400
0
1000000
2000000
3000000
4000000
Chromosome Position
Coverage
0
100
300
0
50000
100000
150000
200000
Plasmid Position
LTPE428
Coverage
0
400
800
0
1000000
2000000
3000000
4000000
Chromosome Position
Coverage
0
200
600
0
50000
100000
150000
200000
Plasmid Position
LTPE429
Coverage
0
400
800
120
0
1000000
2000000
3000000
4000000
Chromosome Position
Coverage
0
200
600
0
50000
100000
150000
200000
Plasmid Position

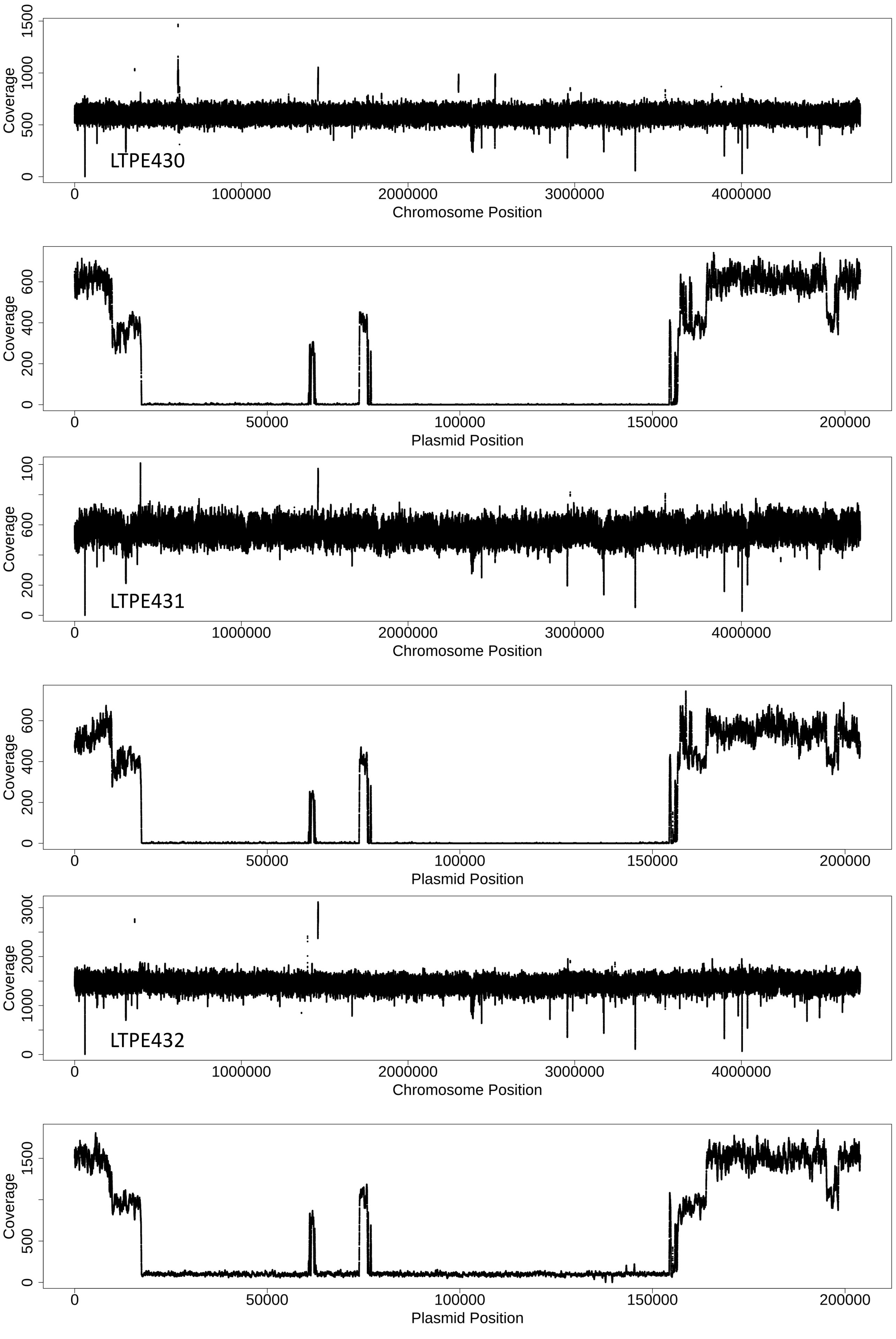

LTPE430
Coverage
0
500
1000
1500
Chromosome Position
0
1000000
2000000
3000000
4000000
Coverage
0
200
400
600
Plasmid Position
0
50000
100000
150000
200000
LTPE431
Coverage
0
200
600
100
Chromosome Position
0
1000000
2000000
3000000
4000000
Coverage
0
200
400
600
Plasmid Position
0
50000
100000
150000
200000
LTPE432
Coverage
0
1000
2000
Chromosome Position
0
1000000
2000000
3000000
4000000
Coverage
0
500
1500
Plasmid Position
0
50000
100000
150000
200000

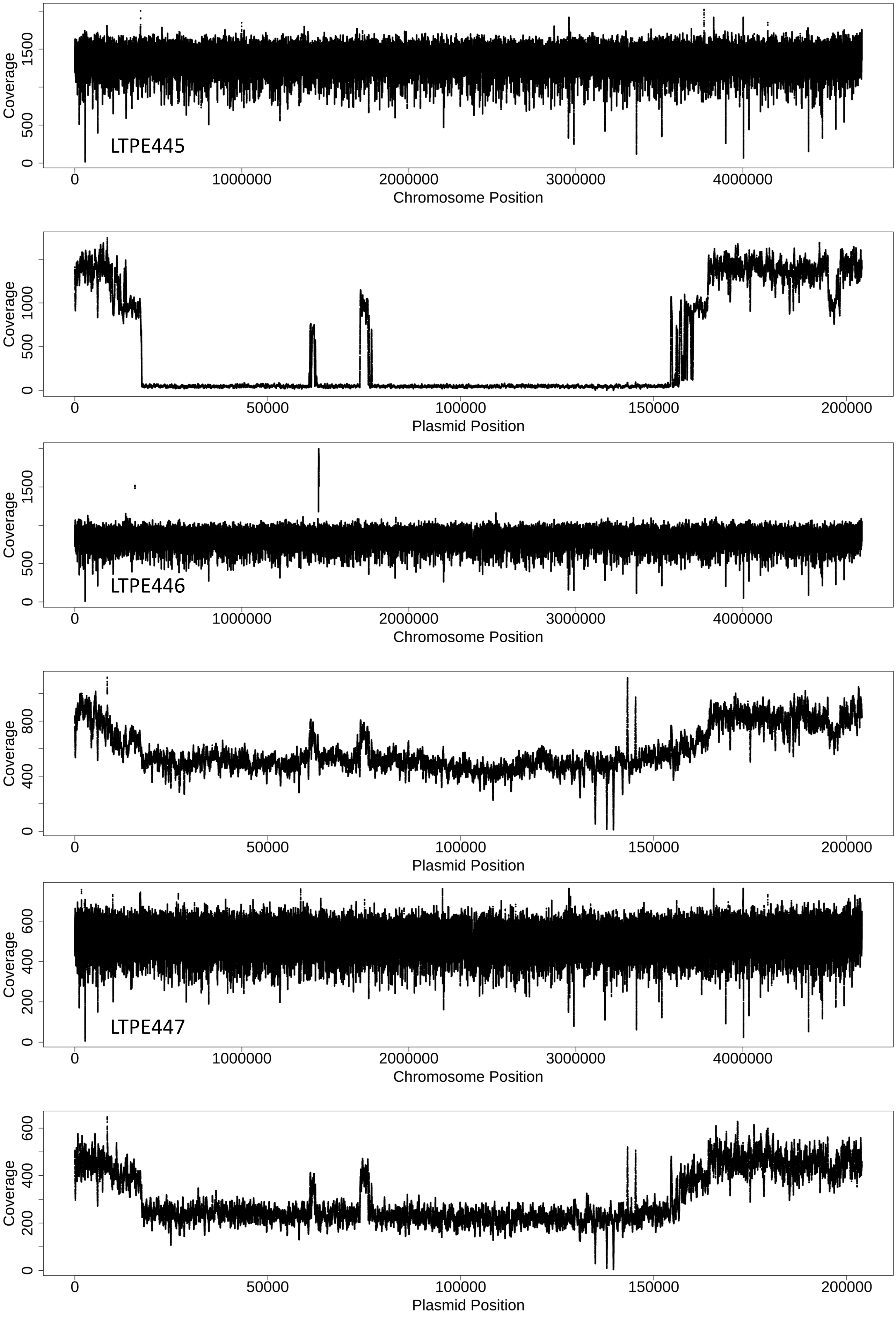
Coverage
0
500
1500
LTPE445
0
1000000
2000000
3000000
4000000
Chromosome Position
Coverage
0
500
1000
0
50000
100000
150000
200000
Plasmid Position
Coverage
0
500
1500
LTPE446
0
1000000
2000000
3000000
4000000
Chromosome Position
Coverage
0
400
800
0
50000
100000
150000
200000
Plasmid Position
Coverage
0
200
400
600
LTPE447
0
1000000
2000000
3000000
4000000
Chromosome Position
Coverage
0
200
400
600
0
50000
100000
150000
200000
Plasmid Position

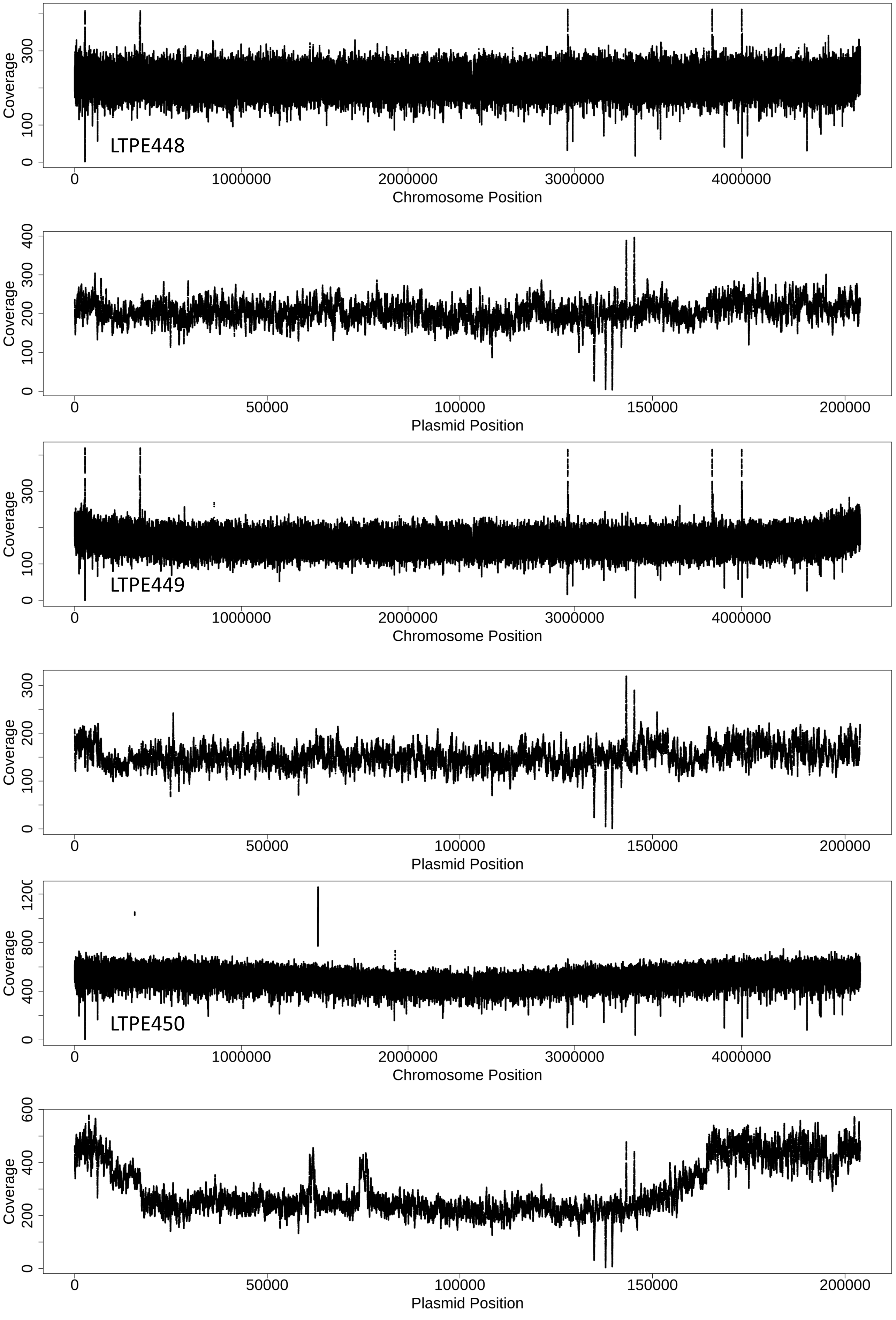

Coverage
0
100
300
LTPE448
0
1000000
2000000
3000000
4000000
Chromosome Position
Coverage
0
100
200
300
400
0
50000
100000
150000
200000
Plasmid Position
Coverage
0
100
300
LTPE449
0
1000000
2000000
3000000
4000000
Chromosome Position
Coverage
0
100
200
300
0
50000
100000
150000
200000
Plasmid Position
Coverage
0
400
800
1200
LTPE450
0
1000000
2000000
3000000
4000000
Chromosome Position
Coverage
0
200
400
600
0
50000
100000
150000
200000
Plasmid Position

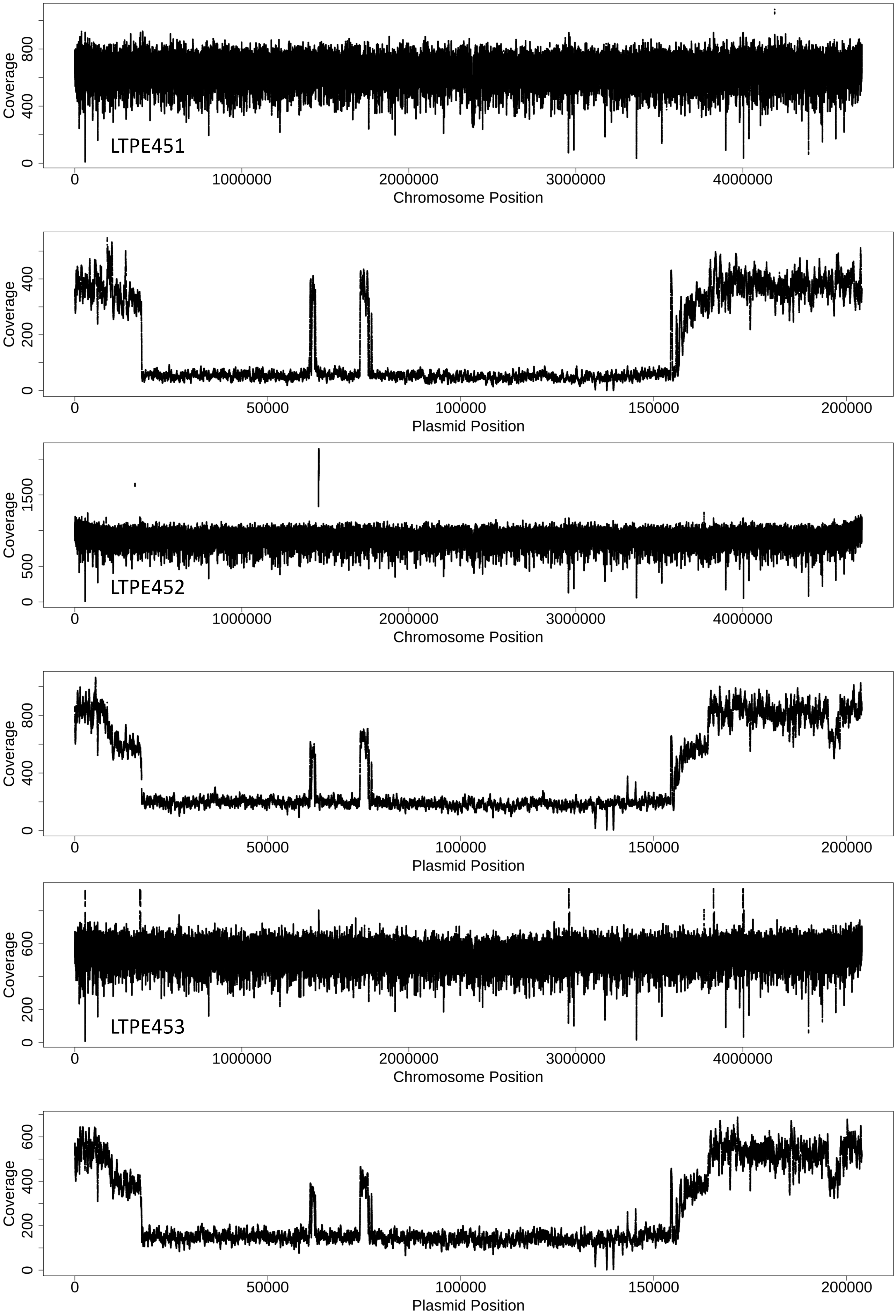

LTPE451
Coverage
Chromosome Position
Coverage
Plasmid Position
LTPE452
Coverage
Chromosome Position
Coverage
Plasmid Position
LTPE453
Coverage
Chromosome Position
Coverage
Plasmid Position

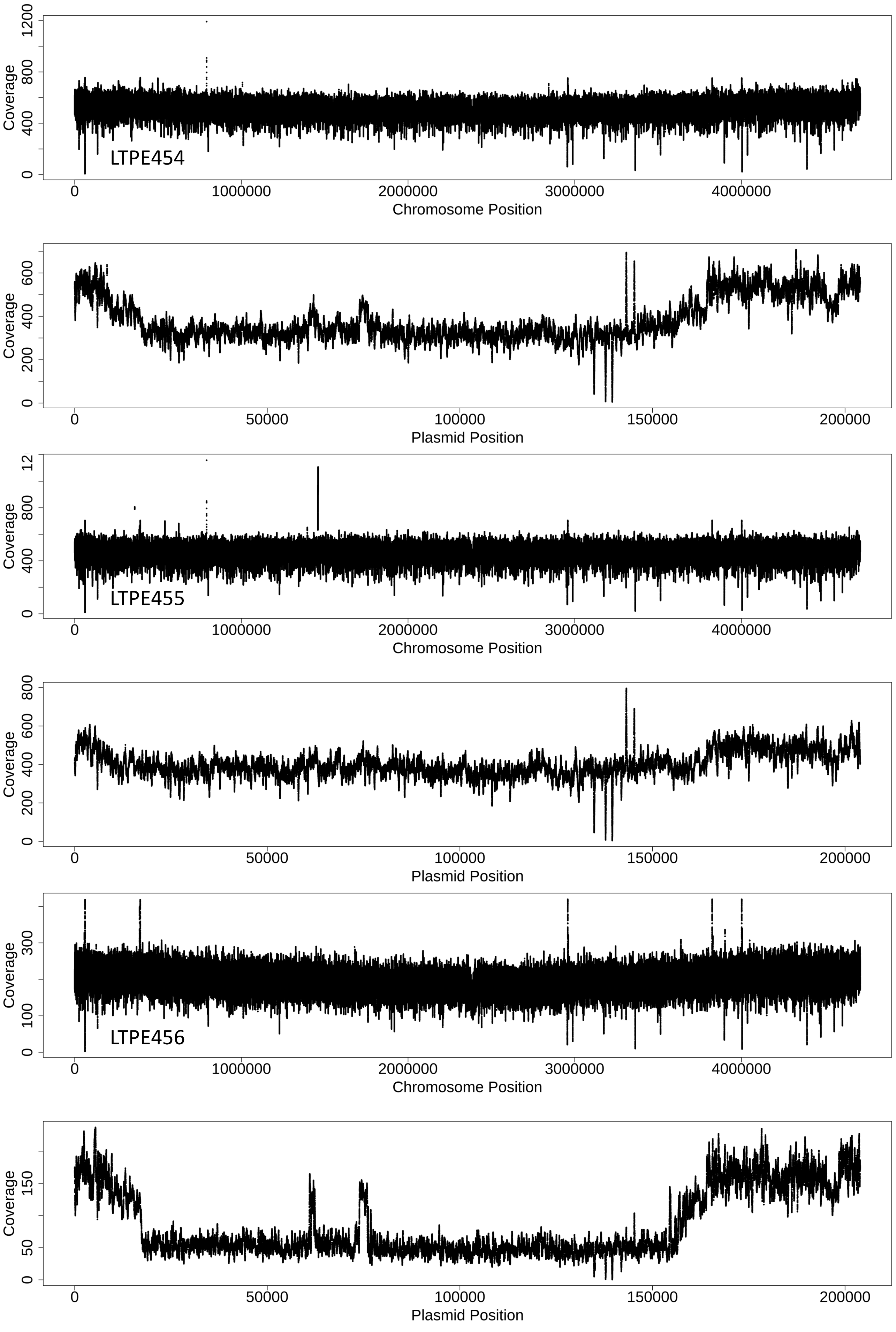
Coverage
0
400
800
1200
LTPE454
0
1000000
2000000
3000000
4000000
Chromosome Position
Coverage
0
200
400
600
0
50000
100000
150000
200000
Plasmid Position
Coverage
0
400
800
LTPE455
0
1000000
2000000
3000000
4000000
Chromosome Position
Coverage
0
200
400
600
800
0
50000
100000
150000
200000
Plasmid Position
Coverage
0
100
300
LTPE456
0
1000000
2000000
3000000
4000000
Chromosome Position
Coverage
0
50
150
0
50000
100000
150000
200000
Plasmid Position

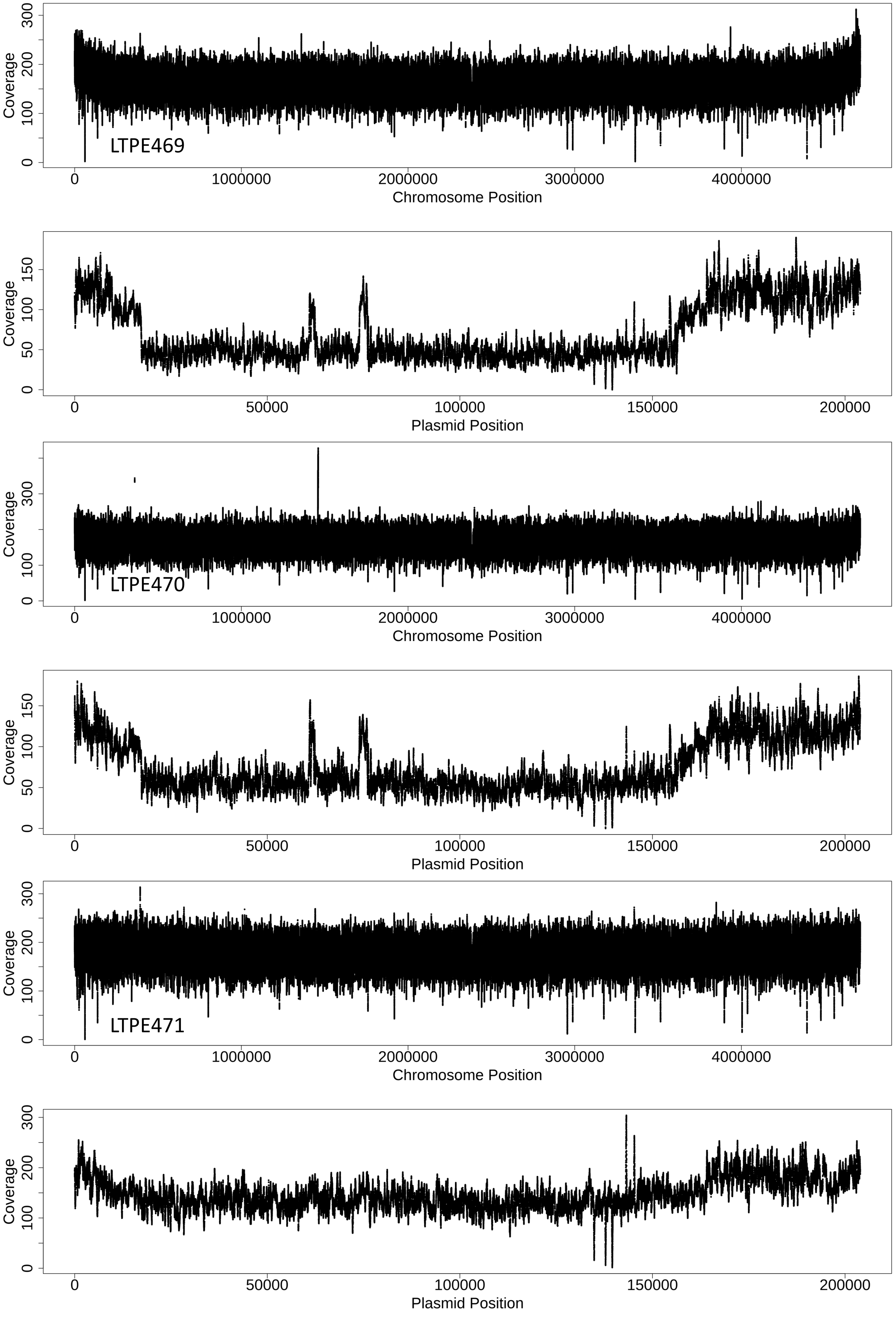

LTPE469
Coverage
0
100
200
300
0
1000000
2000000
3000000
4000000
Chromosome Position
Coverage
0
50
100
150
0
50000
100000
150000
200000
Plasmid Position
LTPE470
Coverage
0
100
300
0
1000000
2000000
3000000
4000000
Chromosome Position
Coverage
0
50
100
150
0
50000
100000
150000
200000
Plasmid Position
LTPE471
Coverage
0
100
200
300
0
1000000
2000000
3000000
4000000
Chromosome Position
Coverage
0
100
200
300
0
50000
100000
150000
200000
Plasmid Position

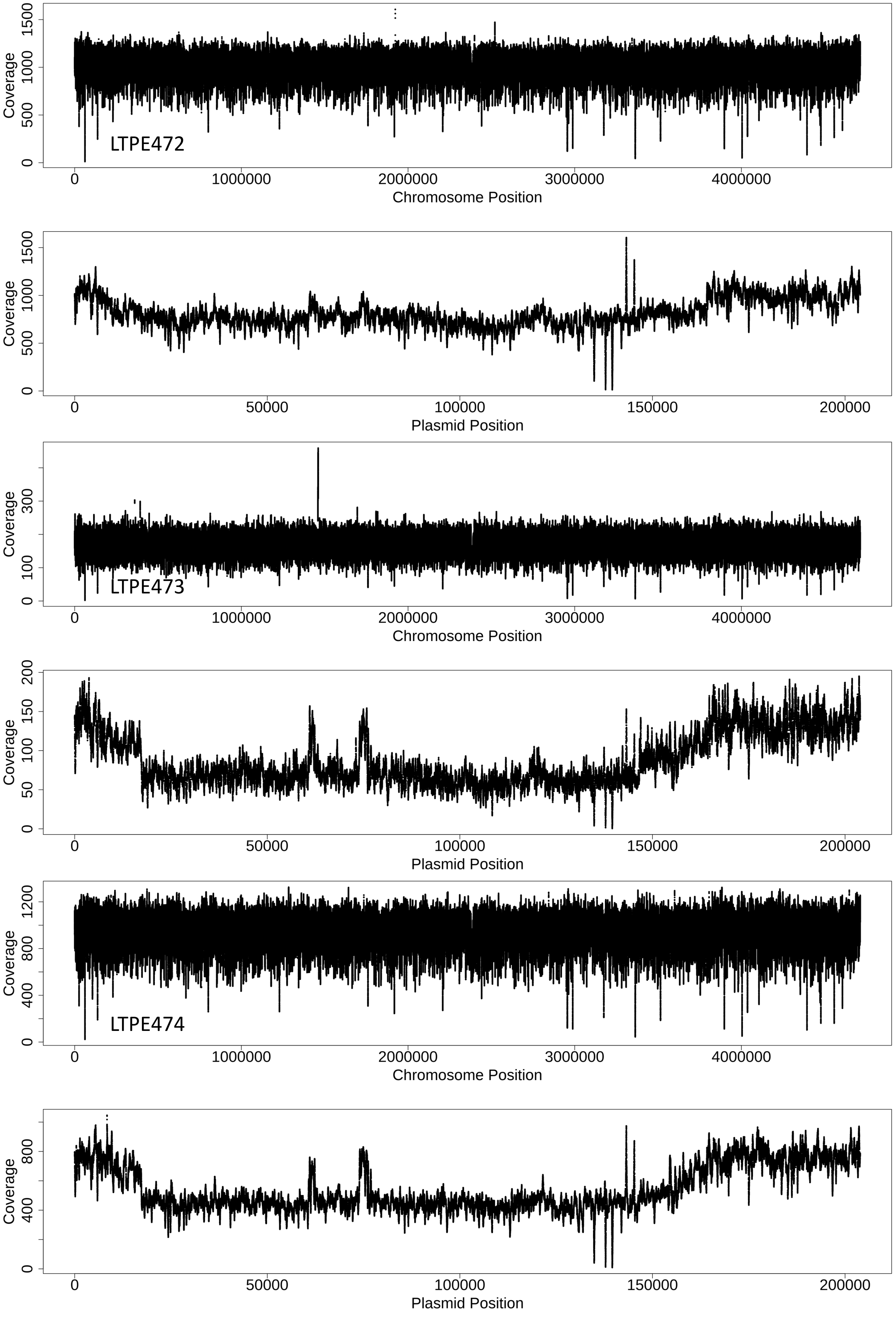

LTPE472
Coverage
0
500
1000
1500
0
1000000
2000000
3000000
4000000
Chromosome Position
Coverage
0
500
1000
1500
0
50000
100000
150000
200000
Plasmid Position
LTPE473
Coverage
0
100
300
0
1000000
2000000
3000000
4000000
Chromosome Position
Coverage
0
50
100
150
200
0
50000
100000
150000
200000
Plasmid Position
LTPE474
Coverage
0
400
800
1200
0
1000000
2000000
3000000
4000000
Chromosome Position
Coverage
0
400
800
0
50000
100000
150000
200000
Plasmid Position

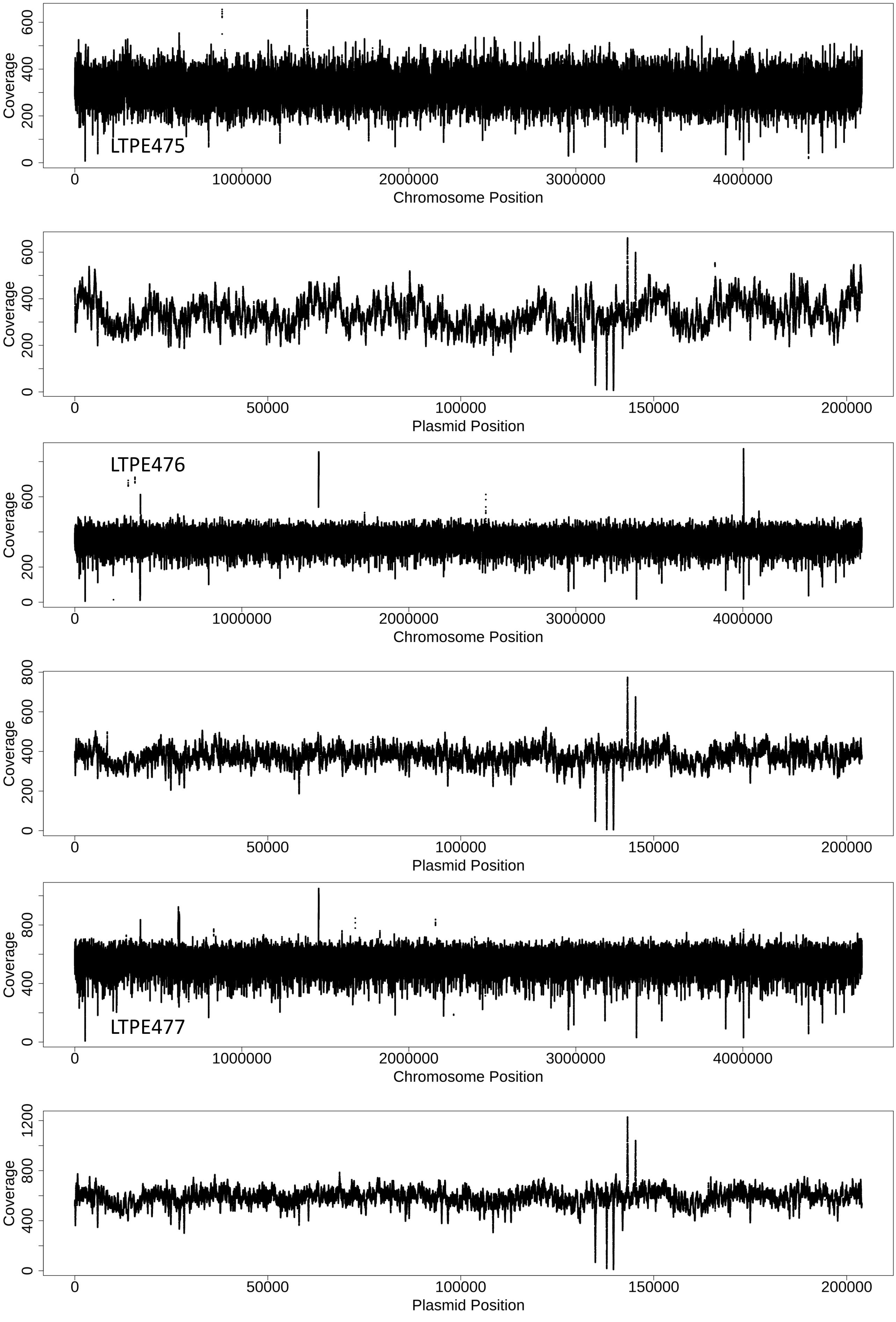
LTPE475
Coverage
Chromosome Position
Coverage
Plasmid Position
LTPE476
Coverage
Chromosome Position
Coverage
Plasmid Position
LTPE477
Coverage
Chromosome Position
Coverage
Plasmid Position
0
1000000
2000000
3000000
4000000
50000
100000
150000
200000
200
400
600
800
1200

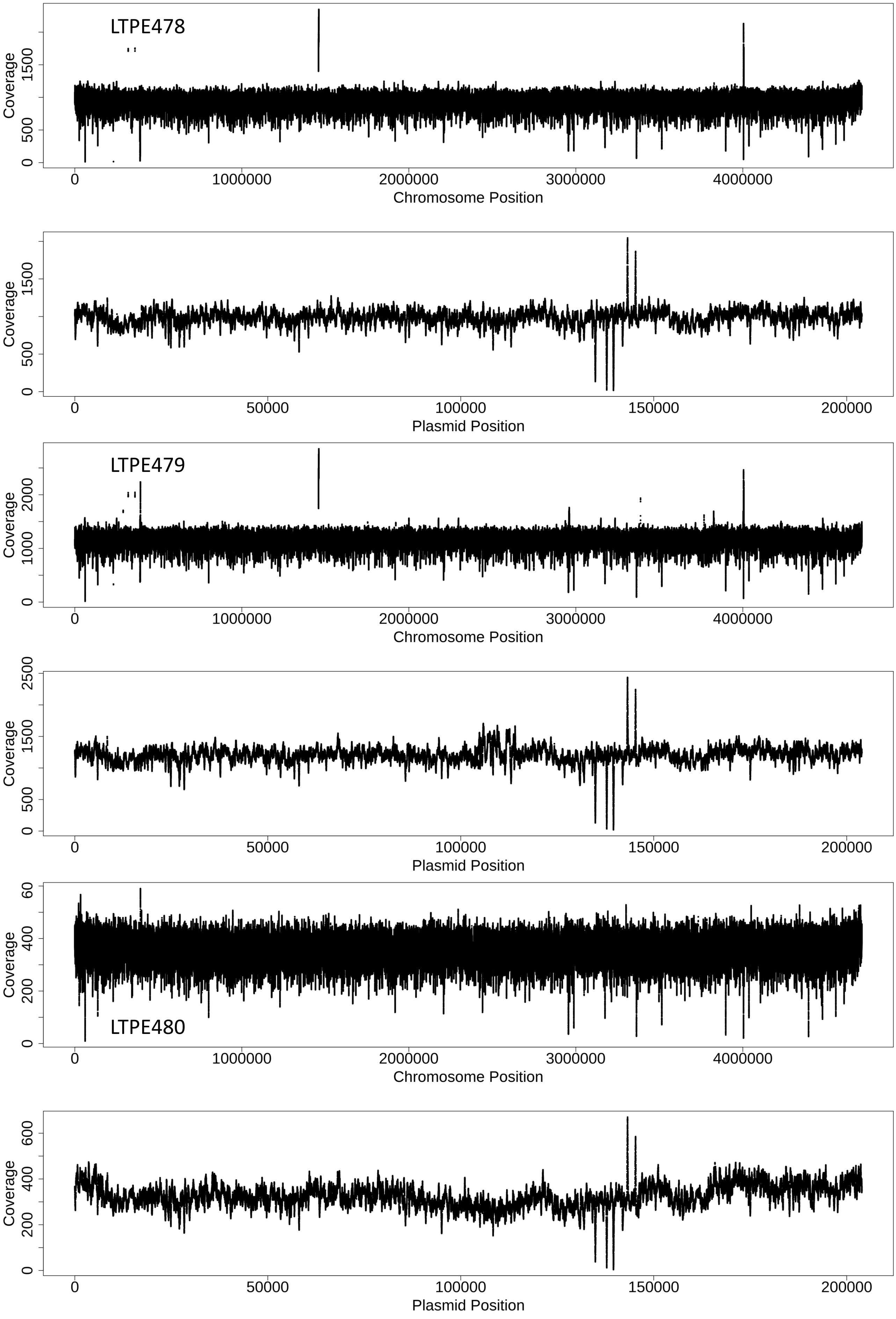
LTPE478
Coverage
Chromosome Position
Coverage
Plasmid Position
LTPE479
Coverage
Chromosome Position
Coverage
Plasmid Position
LTPE480
Coverage
Chromosome Position
Coverage
Plasmid Position

Figure S6. Sequencing coverage for evolved *Alteromonas* EZ55 genomes. Strain numbers indicate the designation of the phytoplankton partner. For each strain, the main chromosome and plasmid are shown separately. “Islands” of coverage in the middle portion of the plasmid represent areas of homology with the main chromosome that caused breseq to mistakenly assign a fraction of coverage to regions of the plasmid that were likely deleted during evolution (see Supplemental Figures S20 and S21).

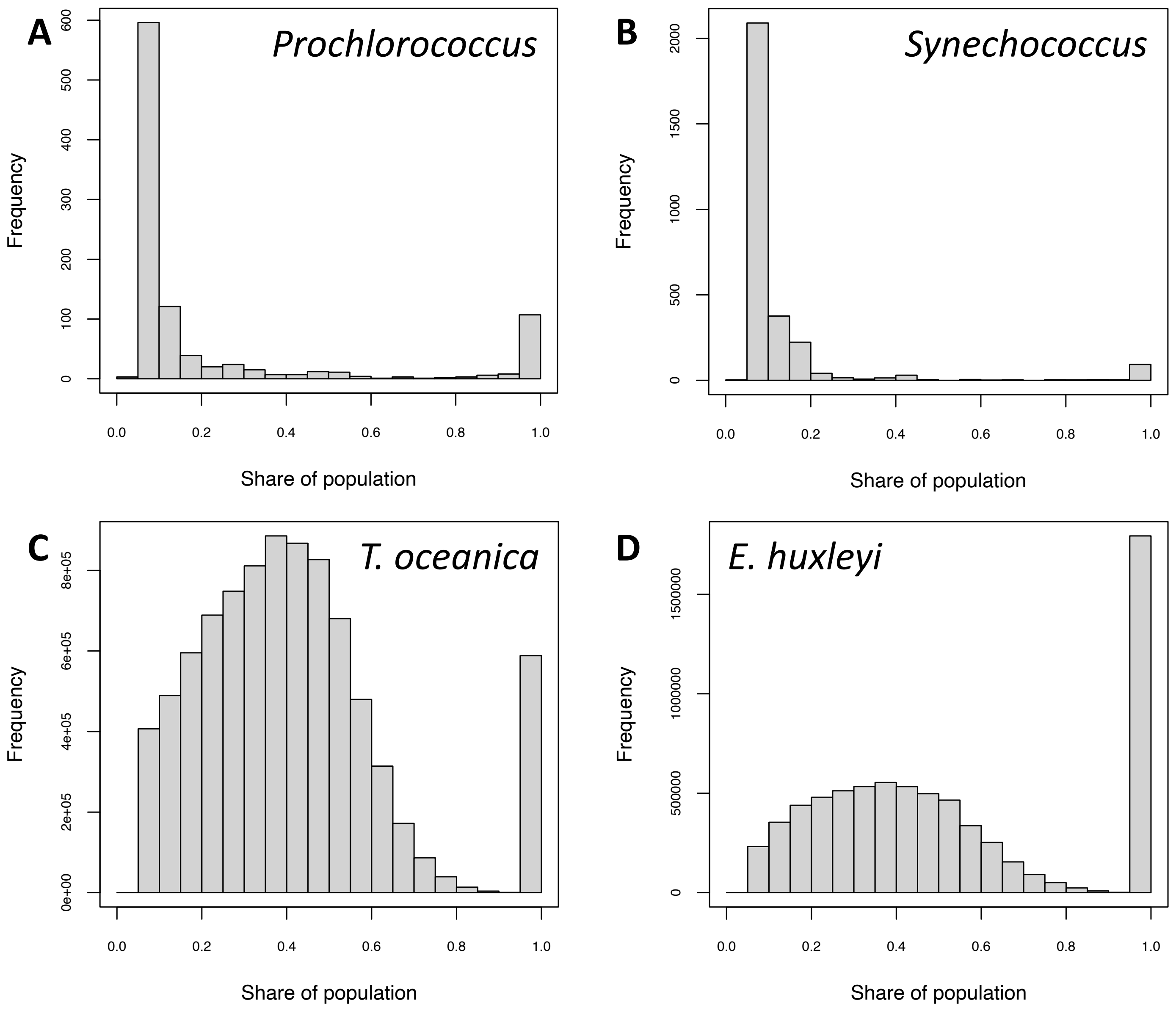

Figure S7. Frequency distributions of mutations in phytoplankton genomes. Histograms show the frequencies of mutations present as a given percentage share of the population, summed across all replicate evolved lineages for A) *Prochlorococcus* MIT9312, B) *Synechococcus* CC9311, C) *T. oceanica* CCMP1005, or D) *E. huxleyi* CCMP371

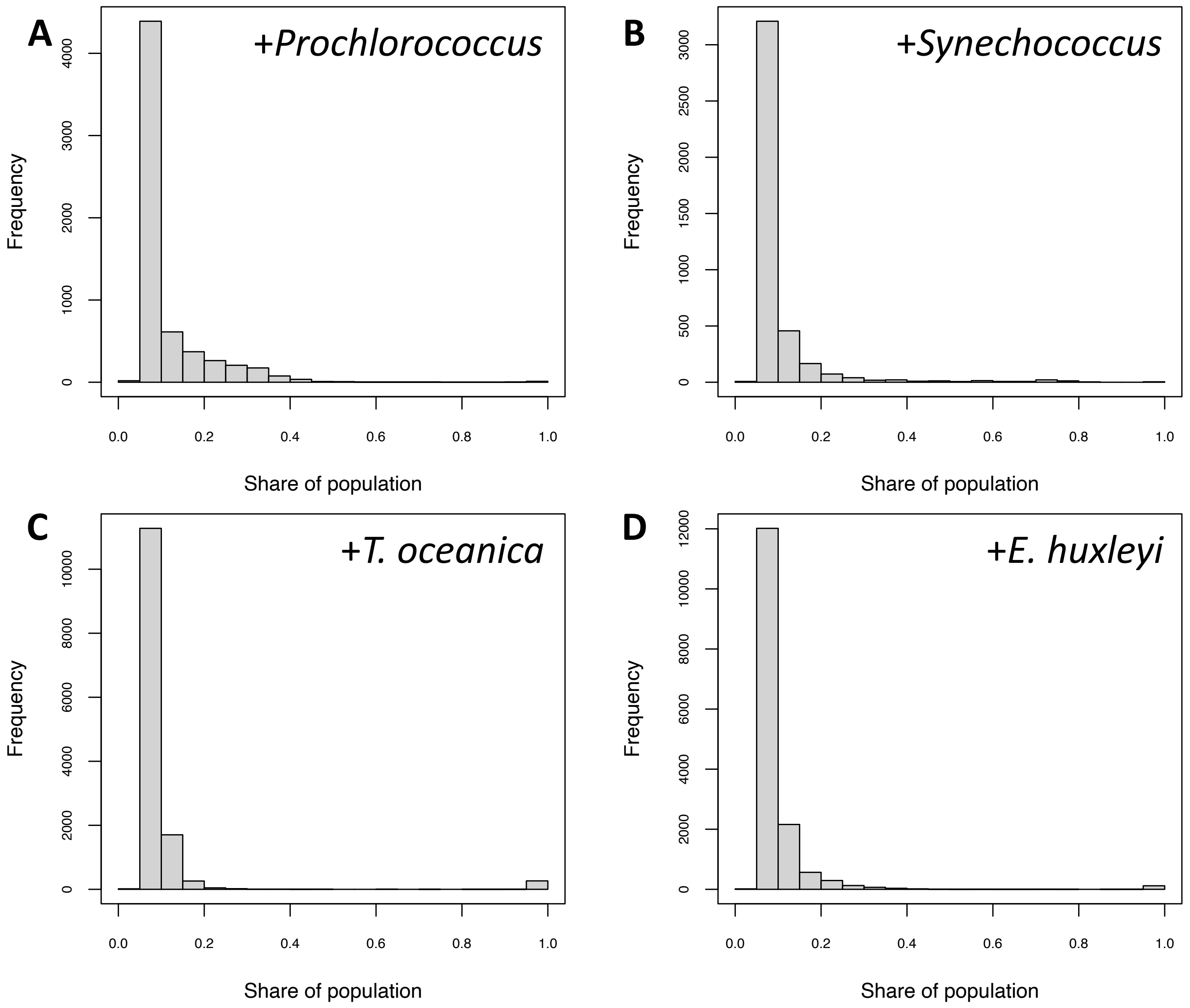

Figure S8. Frequency distributions of mutations in *Alteromonas* genomes. Histograms show the frequencies of mutations present as a given percentage share of the population, summed across all replicate evolved lineages for a given organism. Individual plots represent *Alteromonas* EZ55 genomes evolved alongside A) *Prochlorococcus* MIT9312, B) *Synechococcus* CC9311, C) *T. oceanica* CCMP1005, or D) *E. huxleyi* CCMP371.

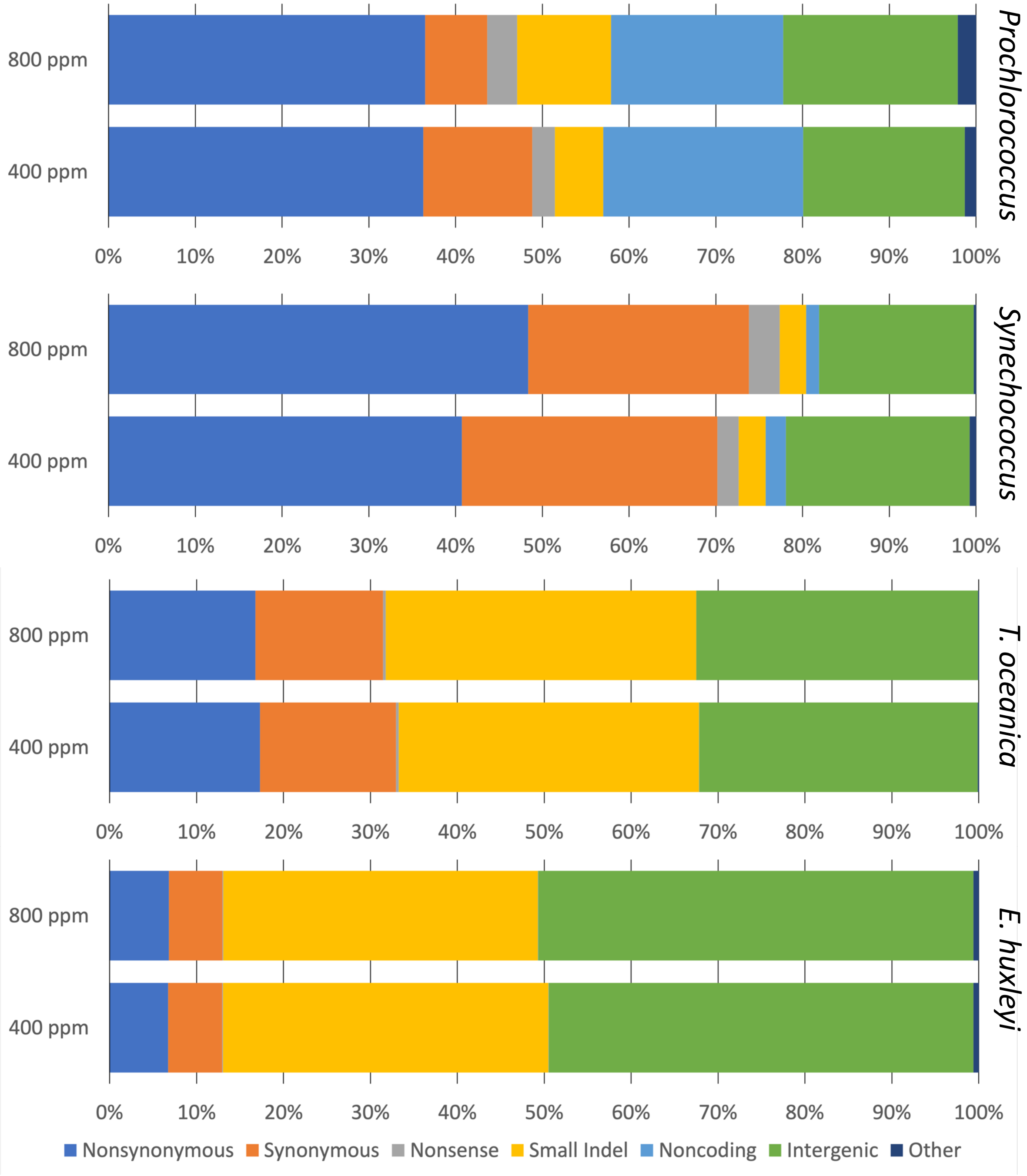

Figure S9. Distributions of mutation types in phytoplankton genomes evolved under different pCO_2 regimes.

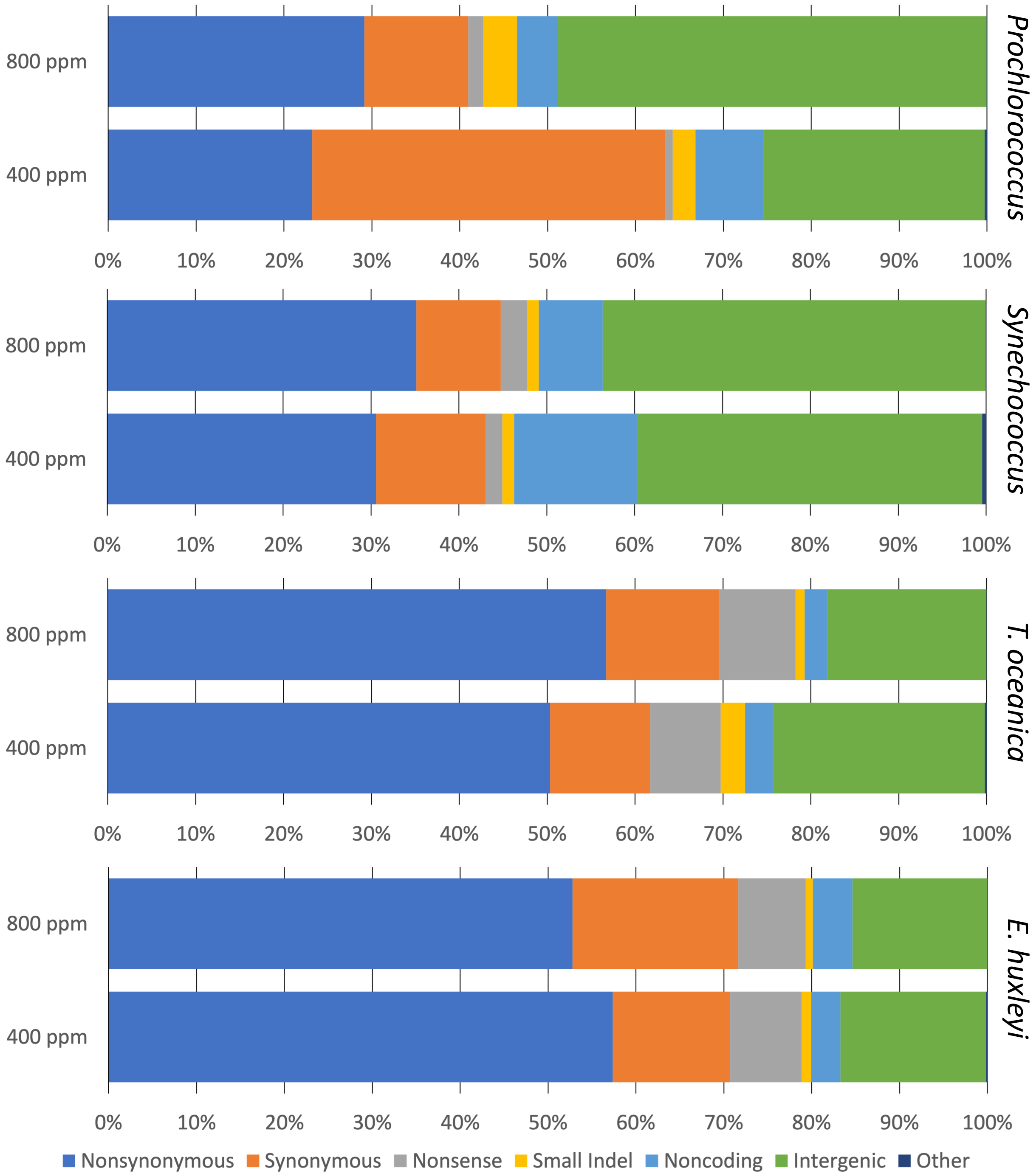

Figure S10. Distributions of mutation types in *Alteromonas* genomes evolved under different pCO_2 regimes in partnership with the indicated phytoplankton strain.

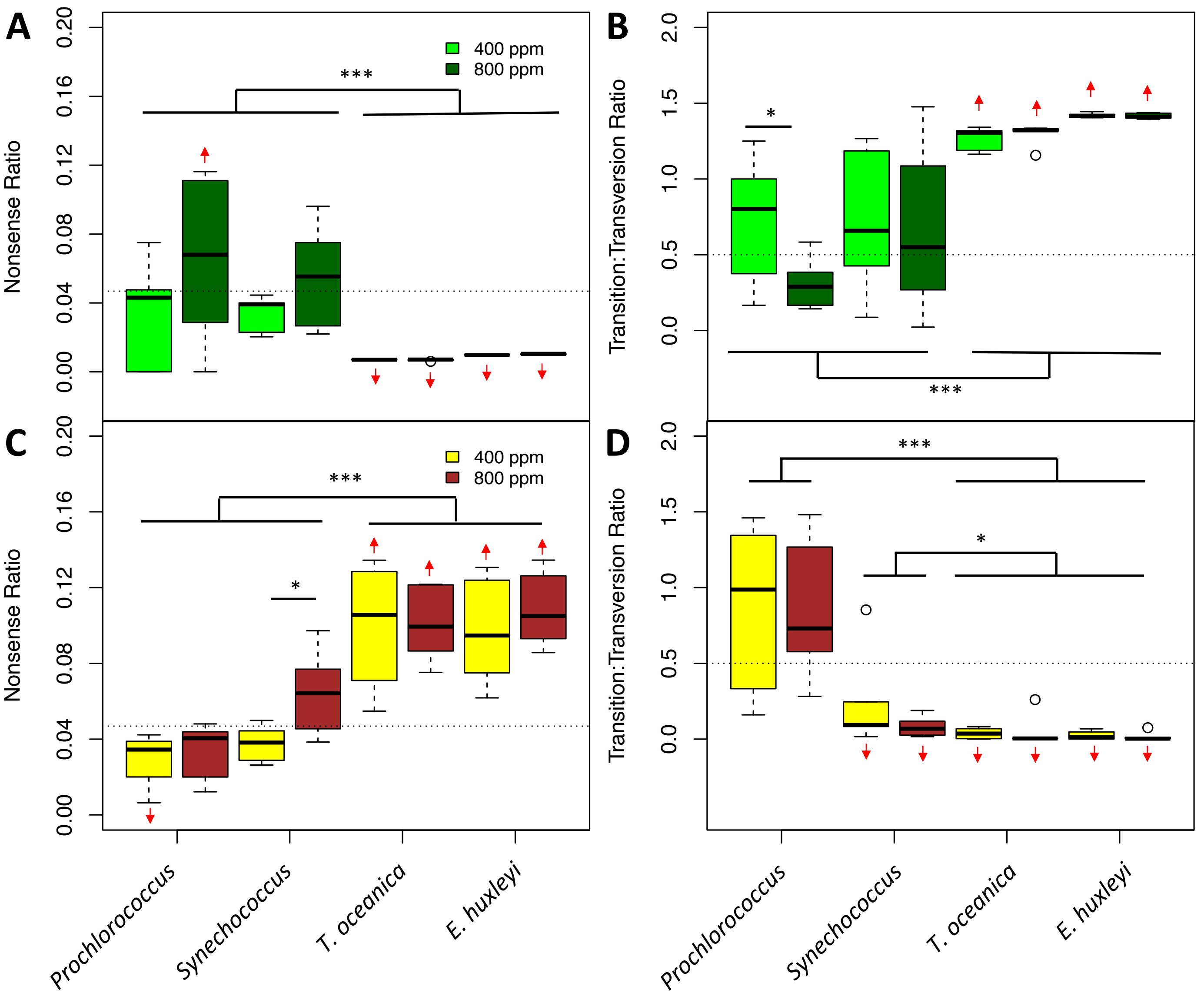

Figure S11. Genomic evidence of adaptive evolution. Plots in A and C show the proportions of codon changes resulting in stop codons (i.e., nonsense mutations) in coding sequences of phytoplankton species (A) or *Alteromonas* strains paired with the indicated phytoplankton species (C). Plots in B and D show the ratios of transitions to transversions. Dashed lines indicate the expected value under neutral evolution; red arrows indicate predicted means significantly higher or lower than this expected value (linear model, 95% confidence interval of the extended marginal mean). Asterisks indicate significant differences between pCO_2 treatments within a species or between species or groups of species: *, $p < 0.05$; ***, $p < 0.001$, ., $p < 0.1$.

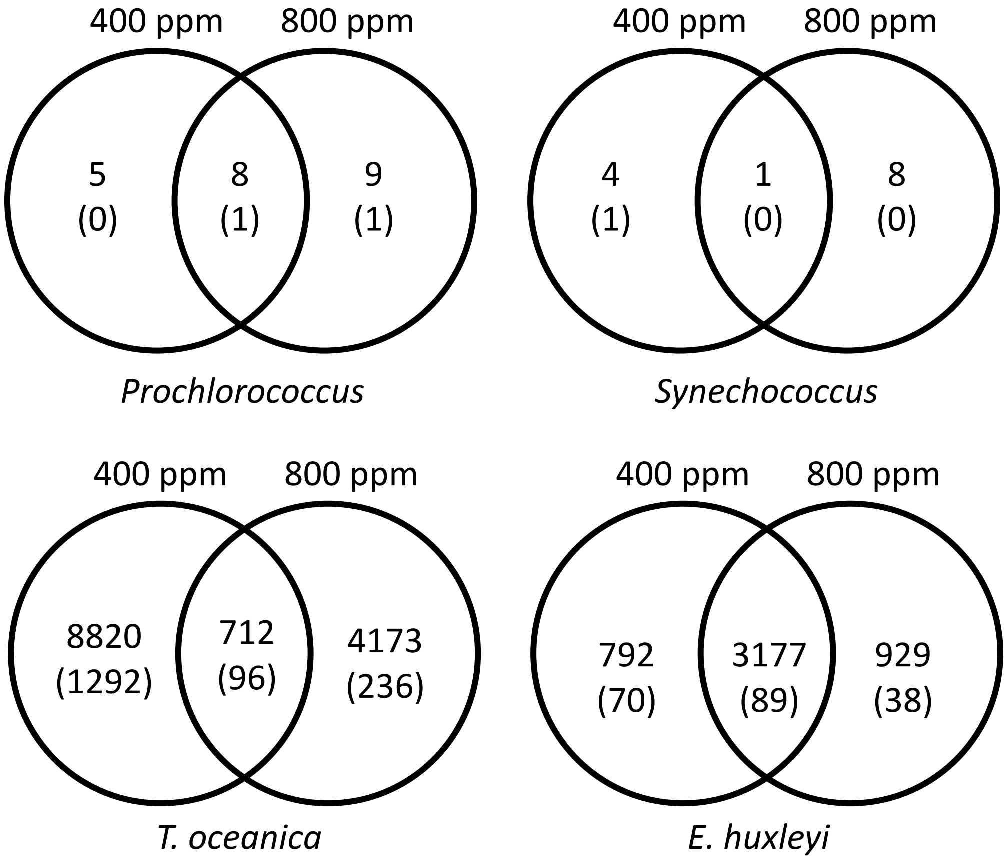

Figure S12. Genes in phytoplankton genomes that were significantly more mutated, by pCO_2 treatment. In order to be considered significantly multiply mutated, a gene had to have either i) more observed nonsynonymous, indel, or promoter mutations than in any of our bootstrapped dummy datasets (see Methods), and to not also have more synonymous mutations than in the dummy datasets, or ii) it had to have at least one observed nonsynonymous mutation in at least 50% of replicately evolved lineages. Values indicate the number of genes passing these criteria in only one of the pCO_2 treatments versus in both; values in parentheses indicate the number of thus identified genes that were also marked as statistically significantly differentially mutated between pCO_2 treatments in a linear model.

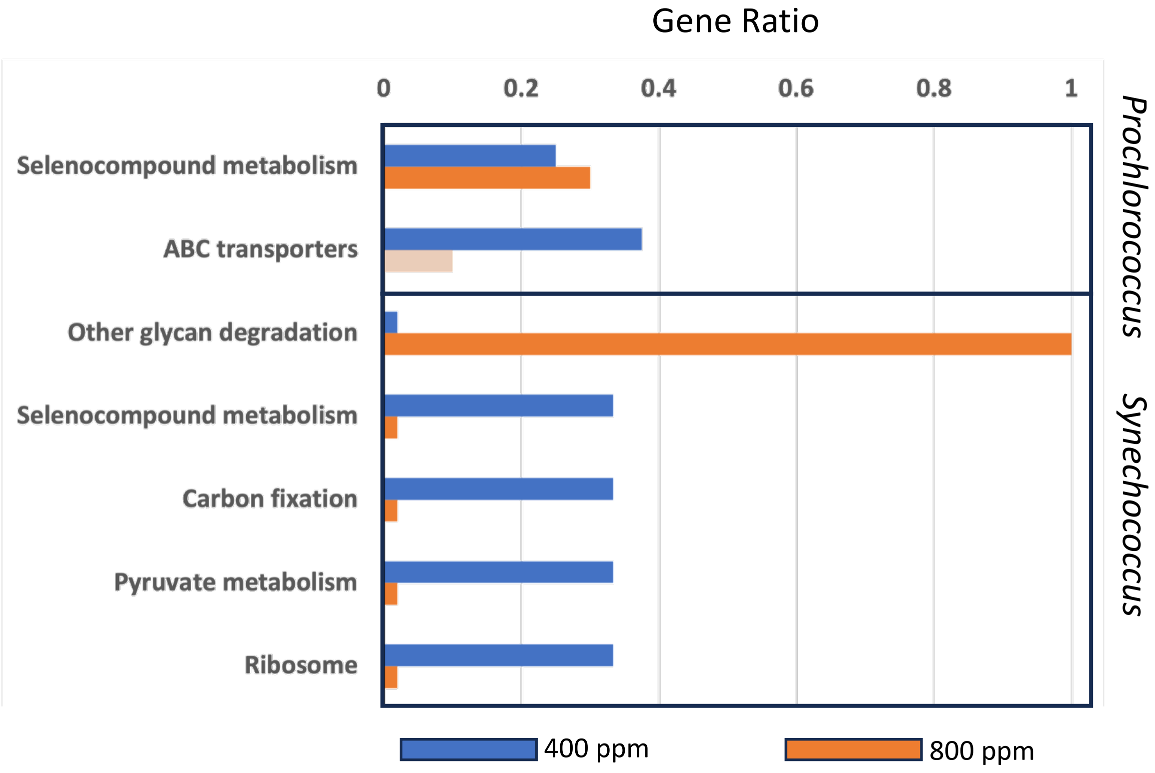

Figure S13. Over-representation analysis of cyanobacterial mutations. Genes passing our multiple mutation screening criteria were assigned to KEGG pathways as described in the Methods. Bold colors indicate that the pathway is statistically significantly overrepresented; paler colors indicate a lack of significance. Very small bars are placeholders only and correspond to absence of mutations observed in the indicated pCO_2 condition.

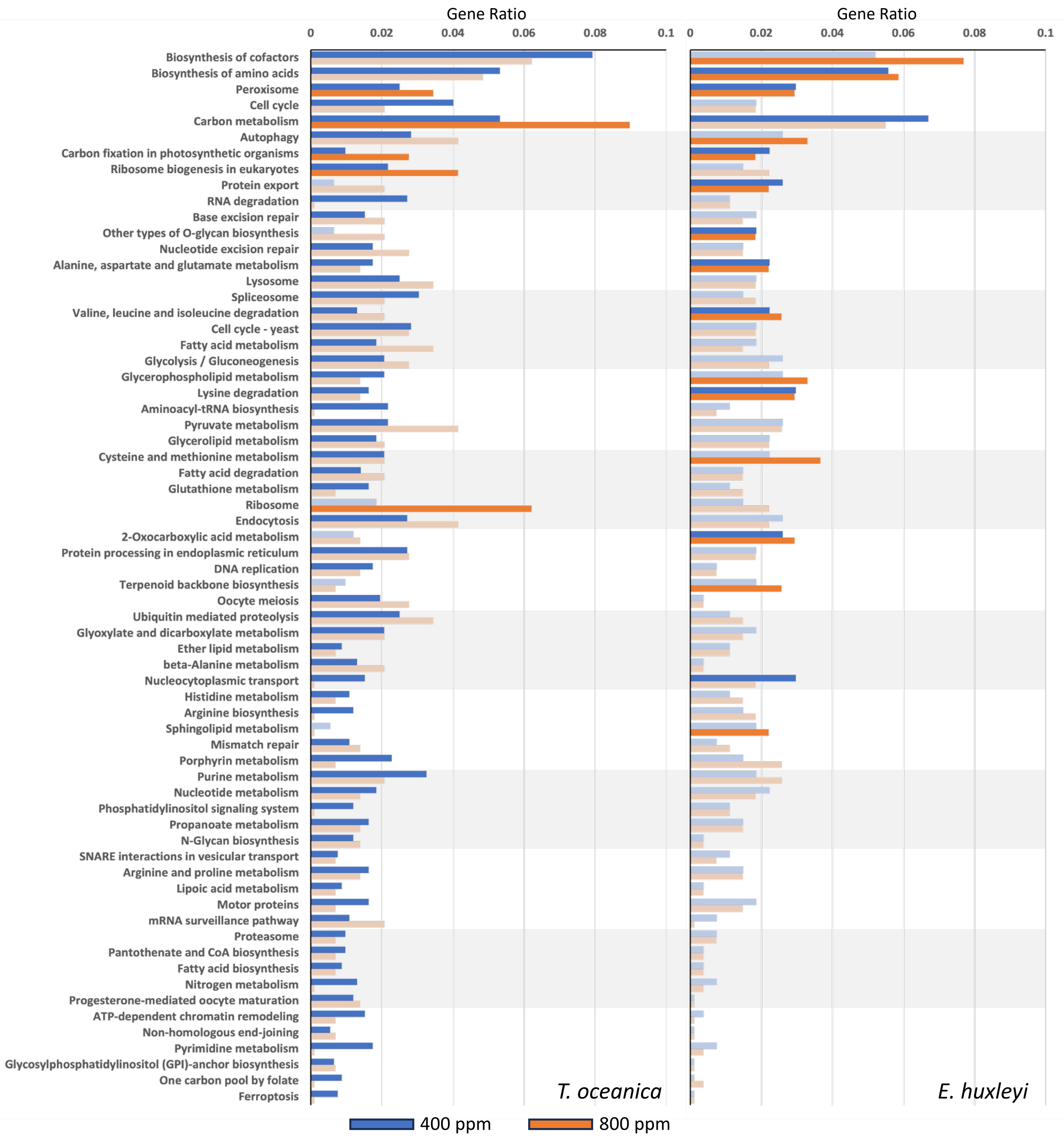

Figure S14. Over-representation analysis of eukaryotic phytoplankton mutations. Genes passing our multiple mutation screening criteria were assigned to KEGG pathways as described in the methods. Bold colors indicate that the pathway is statistically significantly overrepresented; paler colors indicate a lack of significance. Very small bars are placeholders only and correspond to absence of mutations observed in the indicated pCO_2 condition.

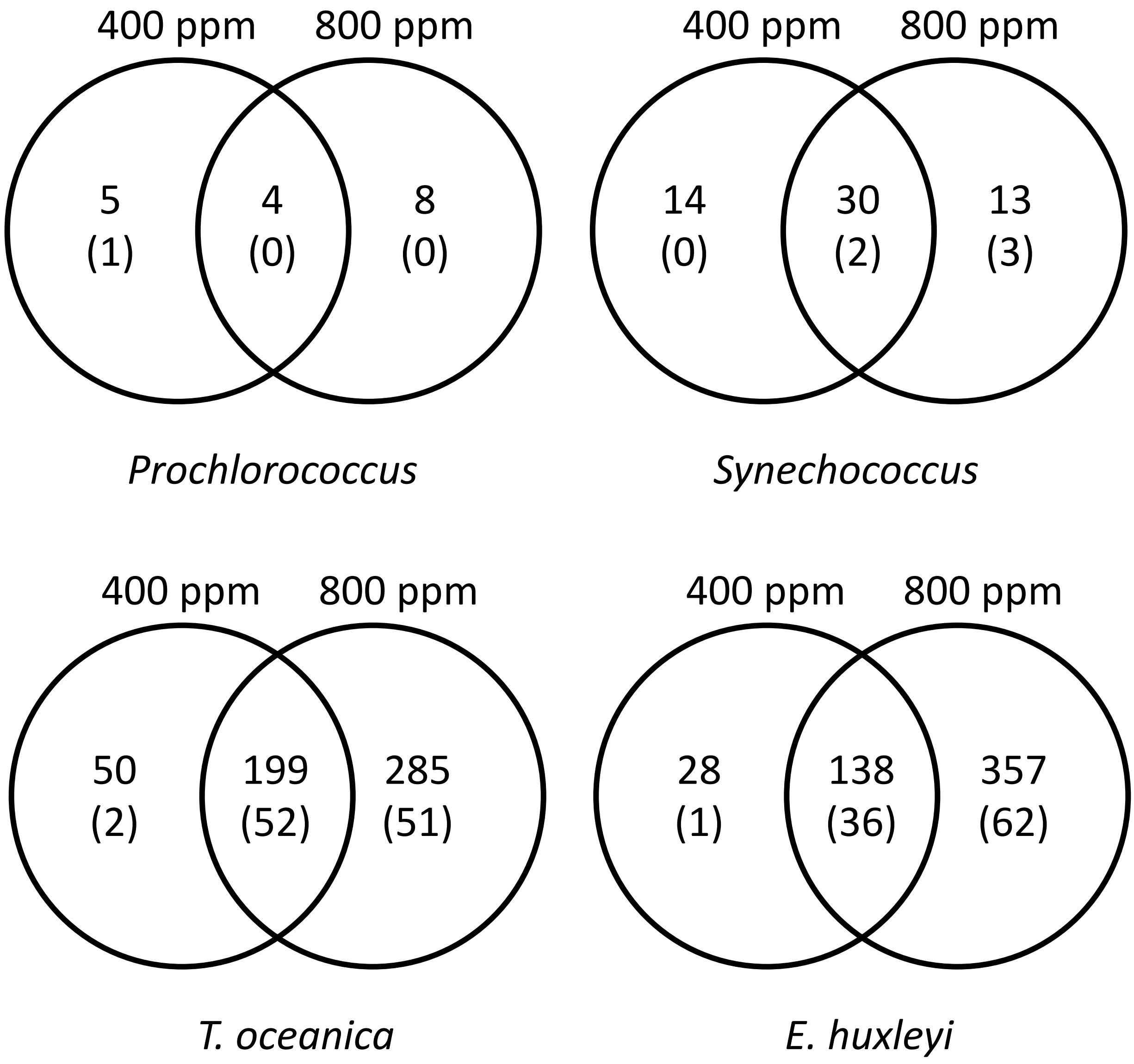

Figure S15. Genes in *Alteromonas* genomes that were significantly more mutated, by pCO_2 treatment. In order to be considered significantly multiply mutated, a gene had to have either i) more observed nonsynonymous, indel, or promoter mutations than in any of our bootstrapped dummy datasets (see Methods), and to not also have more synonymous mutations than in the dummy datasets, or ii) it had to have at least one observed nonsynonymous mutation in at least 50% of replicately evolved lineages. Values indicate the number of genes passing these criteria in only one of the pCO_2 treatments versus in both; values in parentheses indicate the number of thus identified genes that were also marked as statistically significantly differentially mutated between pCO_2 treatments in a linear model.

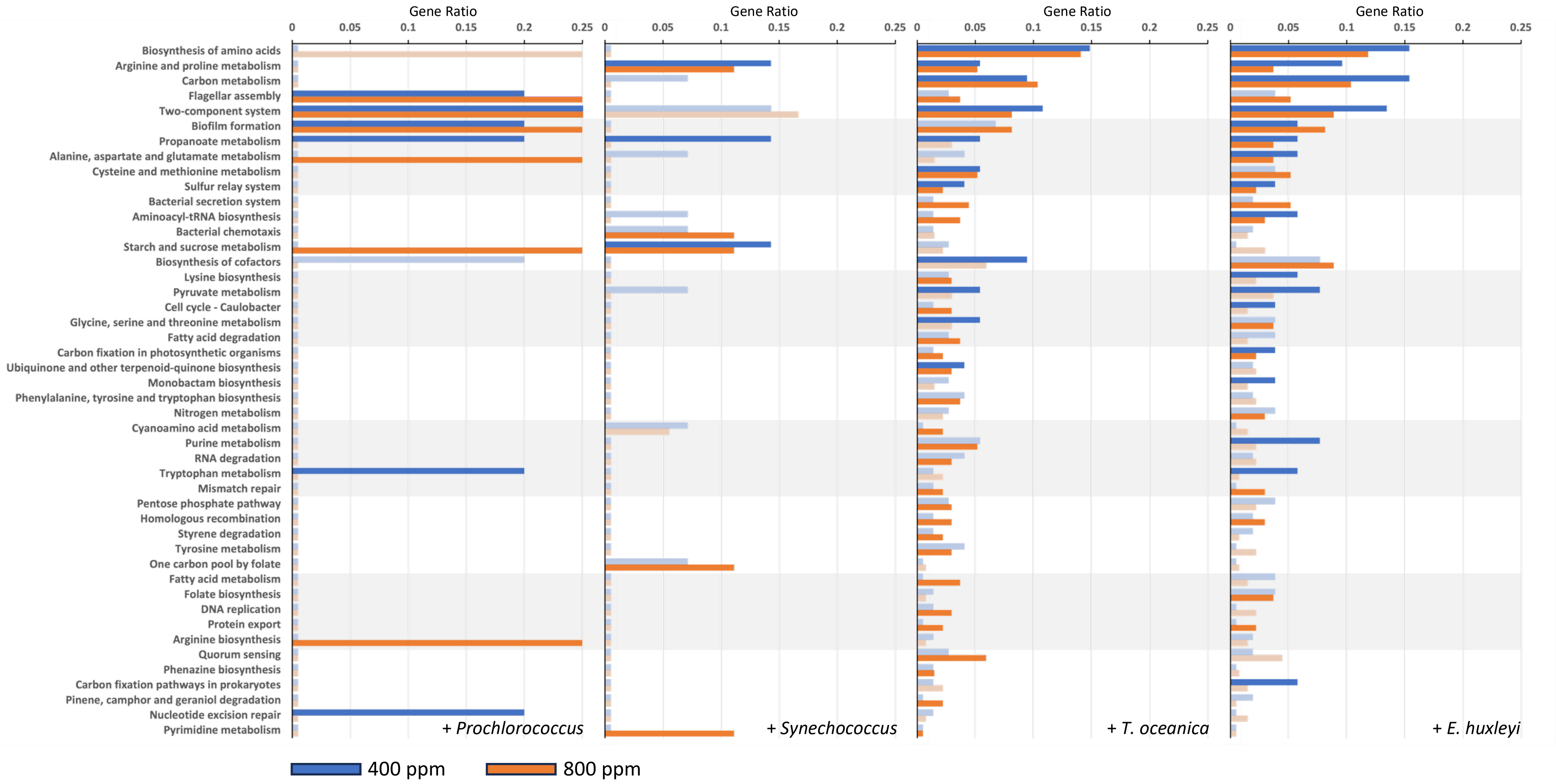

Figure S16. Over-representation analysis of *Alteromonas* EZ55 mutations. Genes passing our multiple mutation screening criteria were assigned to KEGG pathways as described in the Methods. Bold colors indicate that the pathway is statistically significantly overrepresented; paler colors indicate a lack of significance. Very small bars are placeholders only and correspond to absence of mutations observed in the indicated pCO_2 condition.

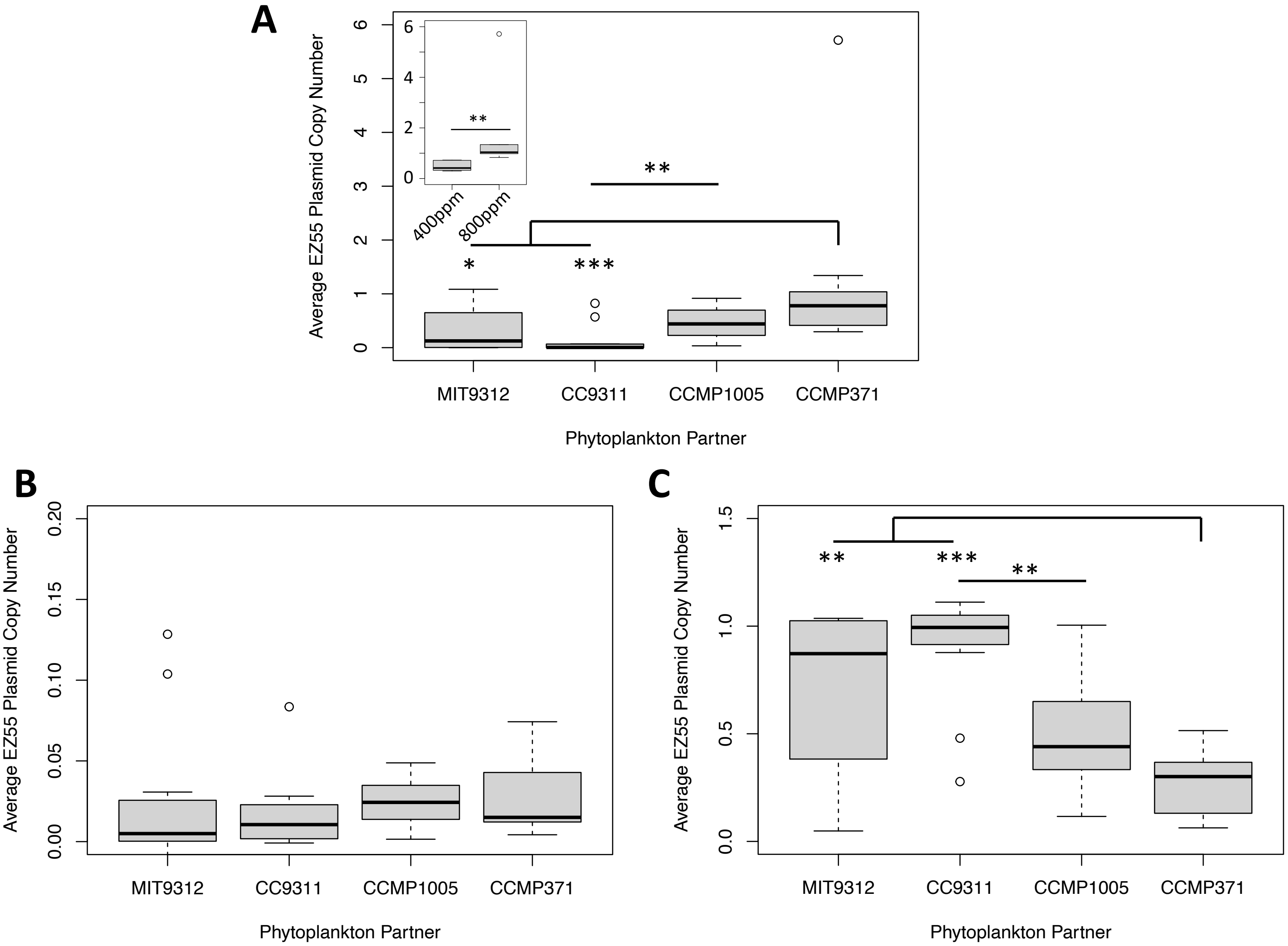

Figure S17. *Alteromonas* plasmid copy number. Copy number was determined by dividing the average coverage of plasmid sequences in diagnostic regions (see materials and methods) by the average coverage of chromosomal sequences. A) Copy number of the full, free *Alteromonas* EZ55 plasmid in co-cultures with *Prochlorococcus* MIT9312*, Synechococcus* CC9311, *T. oceanica* CCMP1005, and *E. huxleyi* CCMP371. The inset figure shows the copy number for EZ55 paired with *E. huxleyi* under different pCO_2 treatments, which were significantly different (Mann-Whitney test, $p = 0.008$). With the exception of strains partnered with *E. huxleyi*, all evolved EZ55 strains had mean copy numbers significantly less than 1, indicating the presence of plasmid-free segregants in the population. B) Copy number of the hypothetical reduced-size version of the EZ55 plasmid. C) Proportion of EZ55 cells with evidence of plasmid insertion in the chromosome. *, $p < 0.05$; **, $p < 0.01$; ***, $p < 0.001$; ., $p < 0.1$.

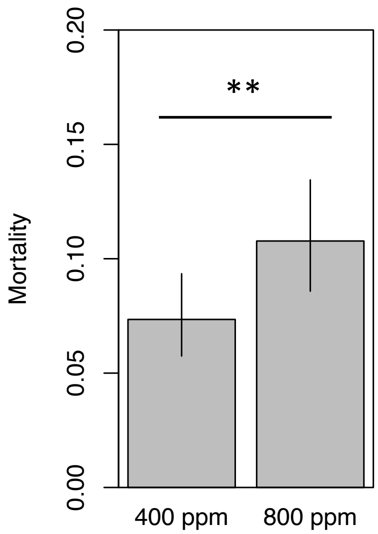

Figure S18. Impact of pCO_2 on *Prochlorococcus* mortality. Ancestral and evolved *Prochlorococcus* cultures were grown either axenically or in co-culture with either ancestral or evolved *Alteromonas* EZ55. The impact of the heterotrophic helper bacterium (or lack thereof) is shown in Figure 4A. Based on model predictions, cultures grown at 800 ppm experienced significantly greater mortality, but there was no interaction between the impact of pCO_2 and heterotrophic bacterium treatment on mortality. **, $p < 0.01$.

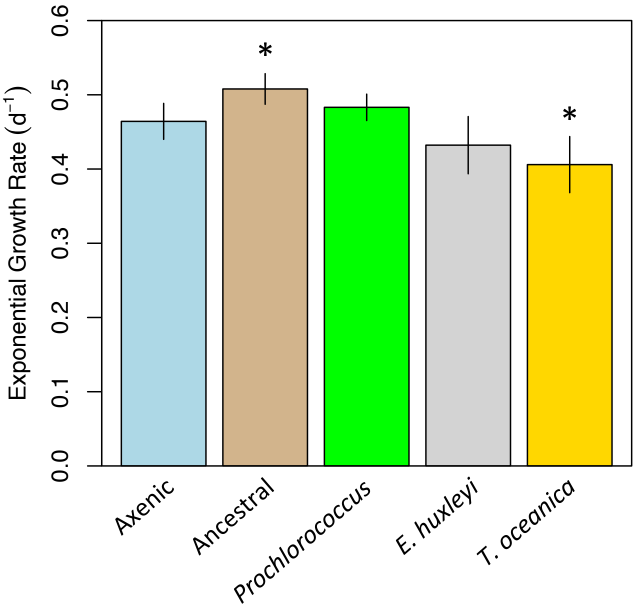

Figure S19. Impact of adaptation to co-culture on ability of EZ55 to improve exponential growth rates of *Prochlorococcus* cultures. Ancestral and evolved populations of *Prochlorococcus* MIT9312 were grown either axenically, in co-culture with ancestral *Alteromonas* EZ55, or with clones of EZ55 isolated from cultures of MIT9312, *E. huxleyi* CCMP371, or *T. oceanica* CCMP1005 after 500 generations of evolution at 400 ppm pCO_2. G and L indicate significantly ($p < 0.05$) greater or lower parameters based on the results of a Dunnett's test comparing each EZ55 treatment to the axenic control, whereas n.s. indicates the result of the comparison was nonsignificant ($p > 0.05$). There was not a significant difference between the impact of EZ55 on ancestral and evolved MIT9312, so the model estimated values are averaged between those treatments. Error bars represent the 95% confidence interval of the extended marginal means estimate of the indicated parameter.

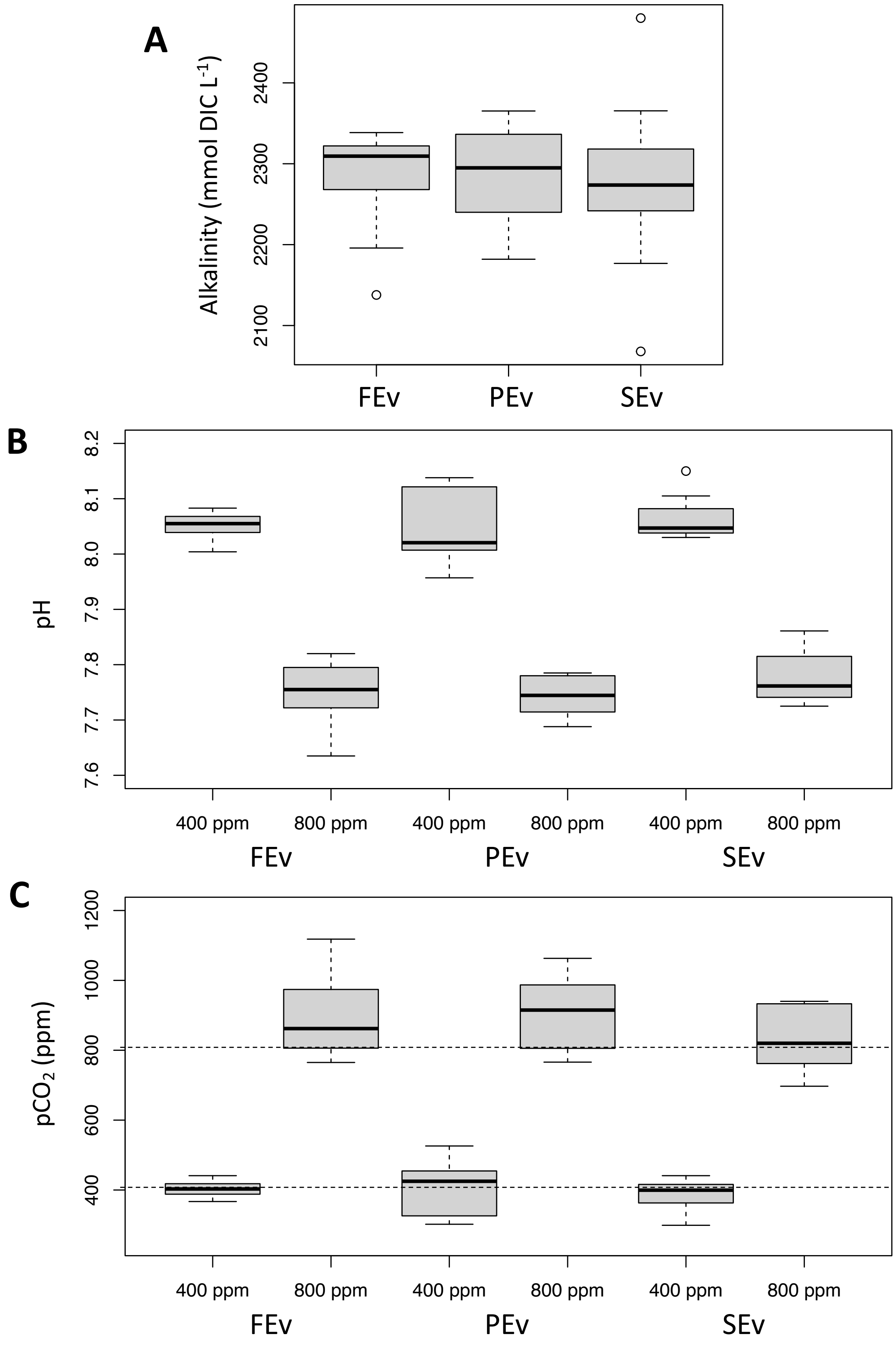

Figure S20. Medium carbonate parameters. Alkalinity (A) and pH (B) were measured by titration and spectrophotometry as described in the methods. pCO_2 (C) was computed based on these parameters using the R package *seacarb*. Dashed lines in (C) indicate the target values of 400 and 800 ppm.

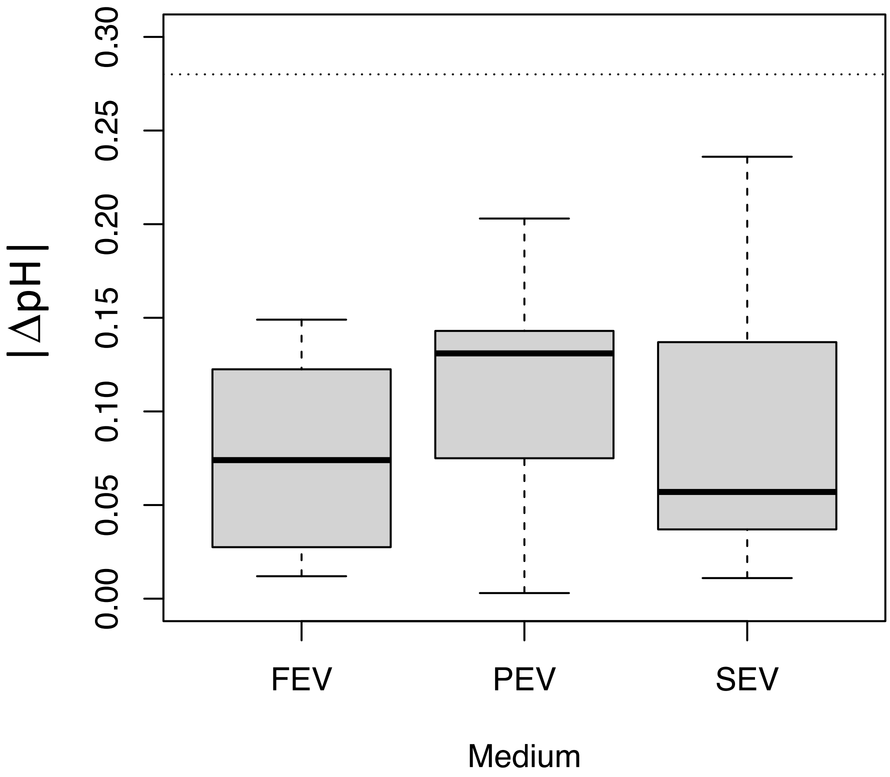

Figure S21. pH drift in media bottles. Several months into the experiment we discovered that pCO_2 concentrations in the lab changed enough to affect the pH of the media. We therefore began measuring media bottles periodically to check for changes and adjust additions accordingly to achieve our pH targets. This plot indicates the range of deviations observed in older media bottles. The dashed line indicates the difference between our pCO_2 treatments' target pH values. Thus, drift in media bottles was substantially smaller than the pH difference between treatments.

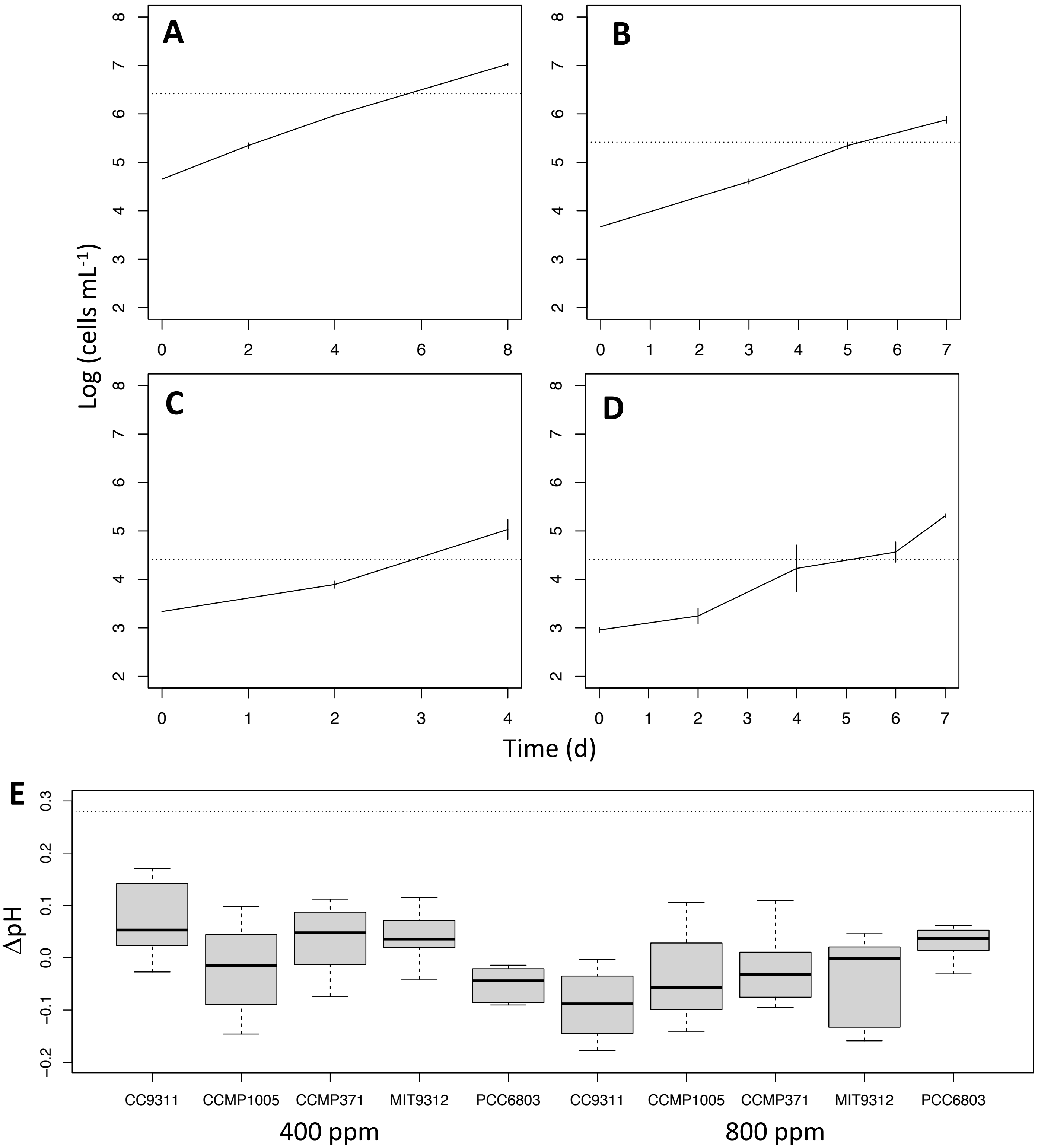

Figure S22. Carrying capacity and pH drift over the culture cycle. Representative growth curves for *Prochlorococcus* (A), *Synechococcus* (B), *T. oceanica* (C), and *E. huxleyi* (D) show that cultures remained in exponential growth phase well past the transfer cutoff cell density (shown by the dashed line in each panel). E) pH shifts from culture inoculation to culture transfer, measured in a series of cultures near the end of the experiment. The dashed line indicates the pH difference between pCO_2 treatments. pH shifts represent shifts toward the pH of the opposite treatment; none of the cultures overlapped the opposite treatment's target pH.

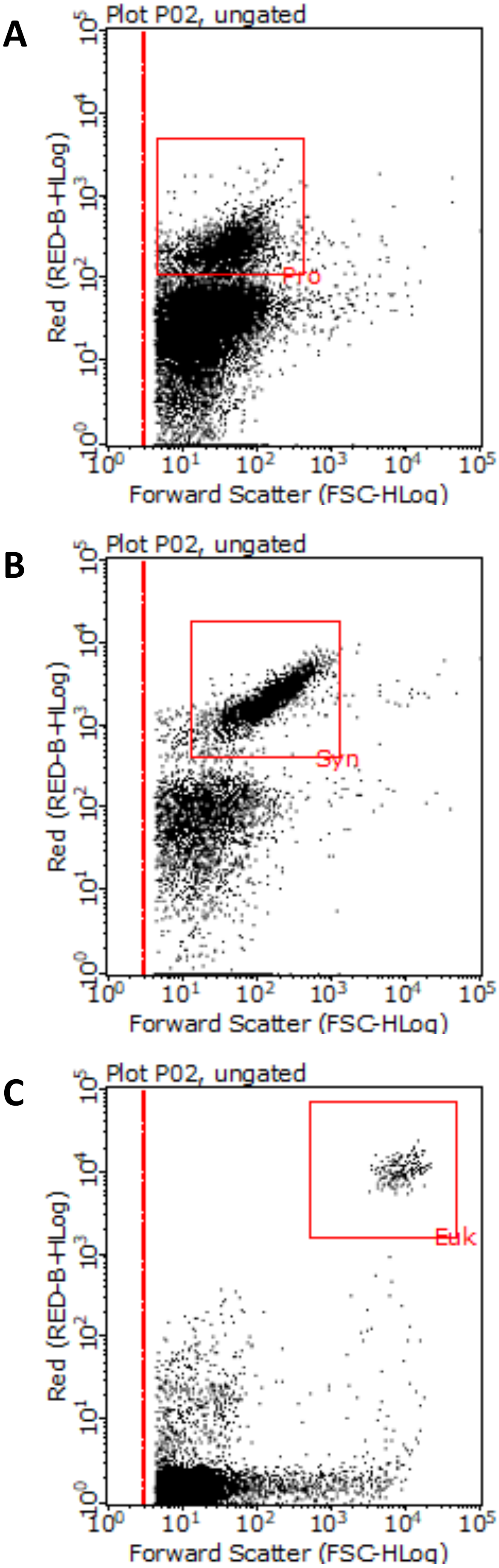

Figure S23. Flow cytometry gates for counting phytoplankton. Cells were observed using a Guava HT1 flow cytometer with the threshold for event detection set on forward scatter (FSC), indicated by the red bar on the left side of the plot. The instrument was set to collect 1000 events with red fluorescence greater than 100 units, or to run for 60 seconds, whichever occurred first. After collection, samples were manually examined using rectangular gates around the clumped points with appropriate FSC/red profiles. Gates were moved as needed manually between samples. Representative plots/gates are shown for A) *Prochlorococcus*, B) *Synechococcus*, and C) *E. huxleyi*. Plots for *T. oceanica* looked nearly identical to those for *E. huxleyi*.

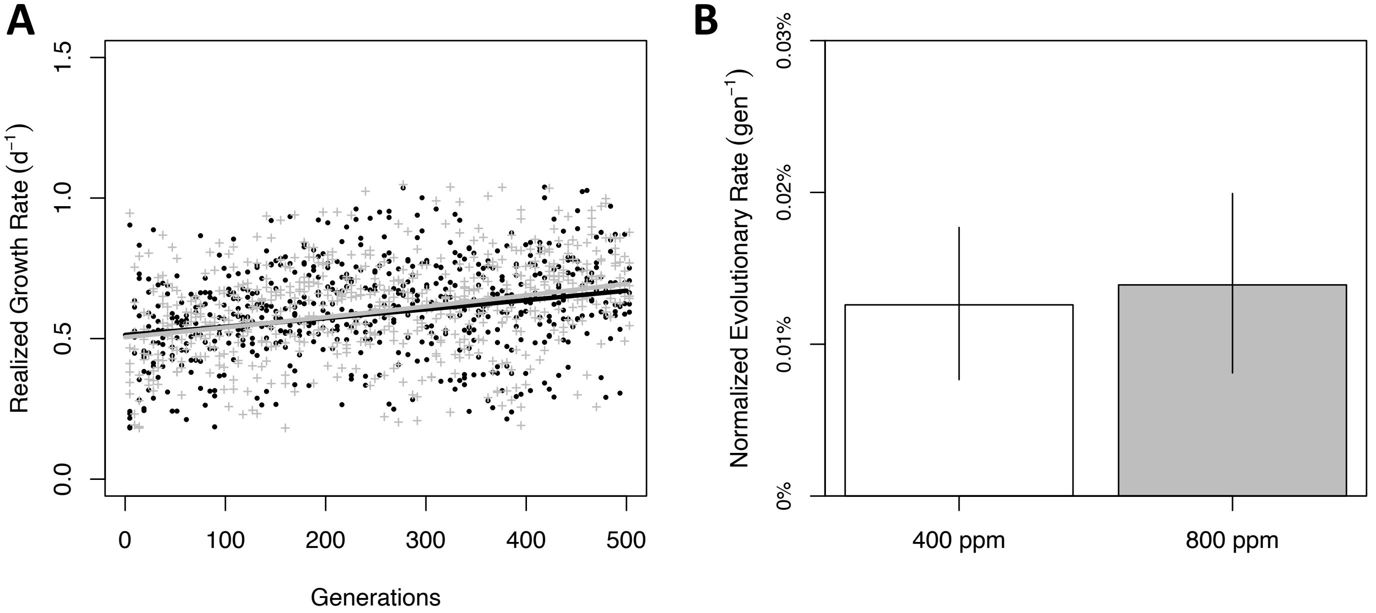

Figure S24. *Synechocystis* PCC6803 evolution. A) Each point represents the realized growth rate of one transfer ($\log_2 26 = 4.7$ generations). Trendlines are regressions of growth rate on generation. Black points and lines = 400 ppm cultures, gray crosses and lines = 800 ppm cultures. B) Normalized evolutionary rates were expressed as the slope of growth rate change (i.e. regression lines in panel A) divided by the estimated ancestral growth rate (i.e. the y-intercept of the regression). Error bars are 95% confidence intervals of the estimates. Tje evolutionary rates were significantly greater than 0 but there was no statistical difference between lines evolving at the two pCO2 treatment levels (linear model, $p > 0.05$).

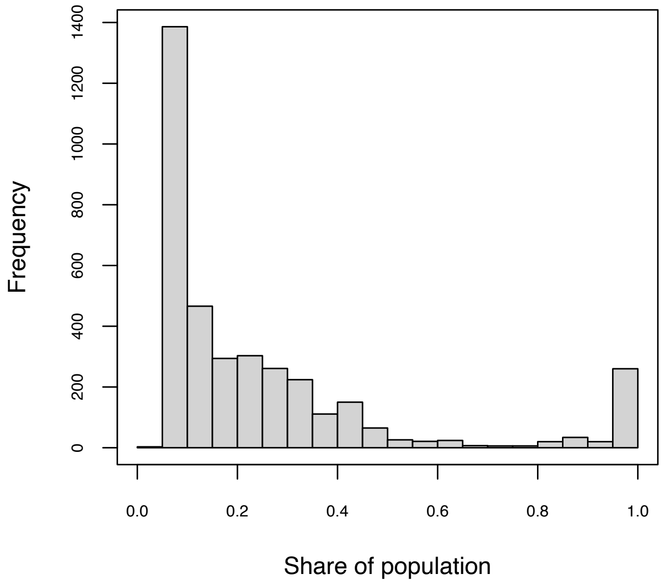

Figure S25. Frequency distributions of mutations in *Synechocystis* genomes. Histograms show the frequencies of mutations present as a given percentage share of the population, summed across all replicate evolved lineages of *Synechocystis* PCC6803. The observed pattern is similar to that of the other evolved bacteria in our study, with a few fixed mutations and many mutations at low frequencies.

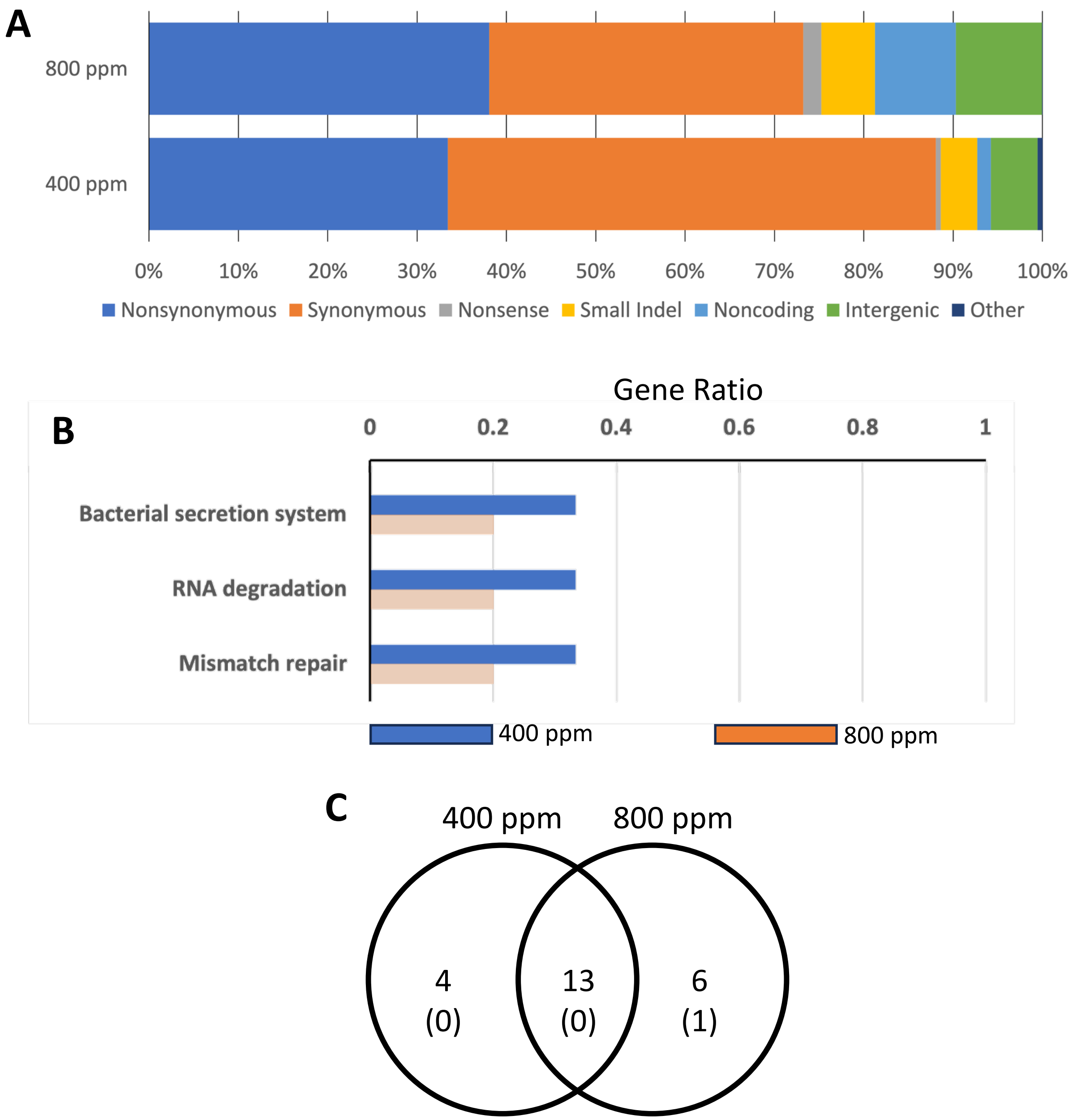

Figure S26. Mutations in *Synechocystis* PCC6803 genomes. A) Distributions of mutation types in Synechocystis genomes evolved under different pCO_2 regimes. B) Genes passing our multiple mutation screening criteria were assigned to KEGG pathways as described in the Methods. Bold colors indicate that the pathway is statistically significantly overrepresented; paler colors indicate a lack of significance. C) Values indicate the number of genes passing our criteria for convergent evolution in only one of the pCO_2 treatments versus in both; values in parentheses indicate the number of thus identified genes that were also marked as statistically significantly differentially mutated between pCO_2 treatments in a linear model.

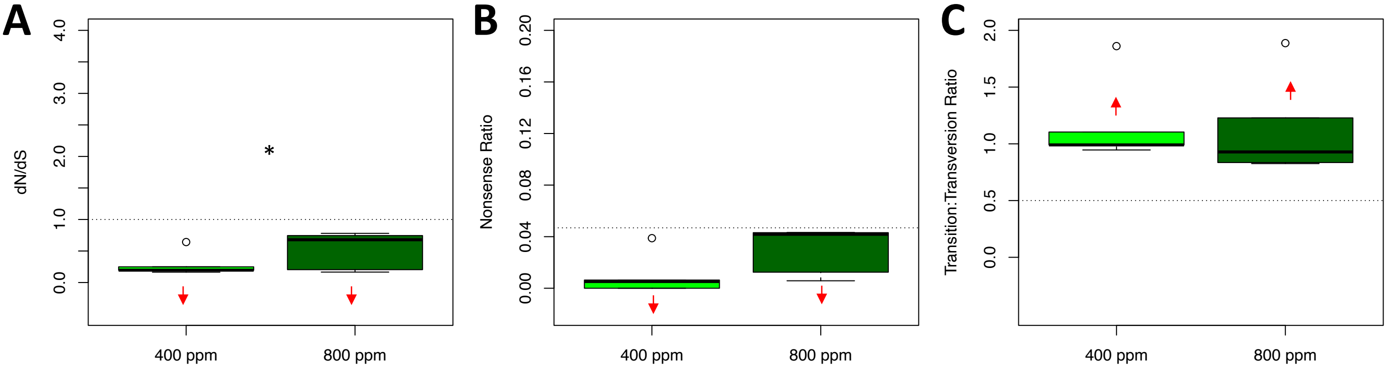

Figure S27. Genomic evidence of adaptive evolution in *Synechocystis* genomes. Plots show A) the ratio of nonsynonymous to synonymous (dN/dS) amino acid changes in coding sequences, B) the proportions of codon changes resulting in stop codons (i.e., nonsense mutations) in coding sequences, or C) the ratios of transitions to transversions. Dashed lines indicate the expected value under neutral evolution; red arrows indicate predicted means significantly higher or lower than this expected value (linear model, 95% confidence interval of the extended marginal mean). *, $p < 0.05$ for the comparison between pCO_2 treatments. These observations are consistent with predominantly conservative evolution for *Synechocystis*, with mild support for increased selection at elevated pCO_2 according to dN/dS.

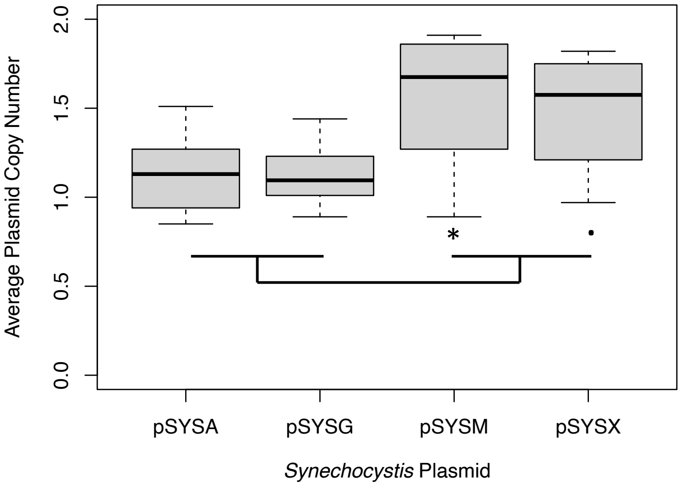

Figure S28. *Synechocystis p*lasmid copy number. Copy number was determined by dividing the average coverage of plasmid sequences by the average coverage of chromosomal sequences. *, $p < 0.05$; **, $p < 0.01$; ***, $p < 0.001$; ., $p < 0.1$. Copy numbers of the two largest plasmids, pSYSM and pSYSX, were significantly higher than the smaller plasmids pSYSA and pSYSG and were significantly or marginally significantly also greater than 1 copy per chromosome on average.

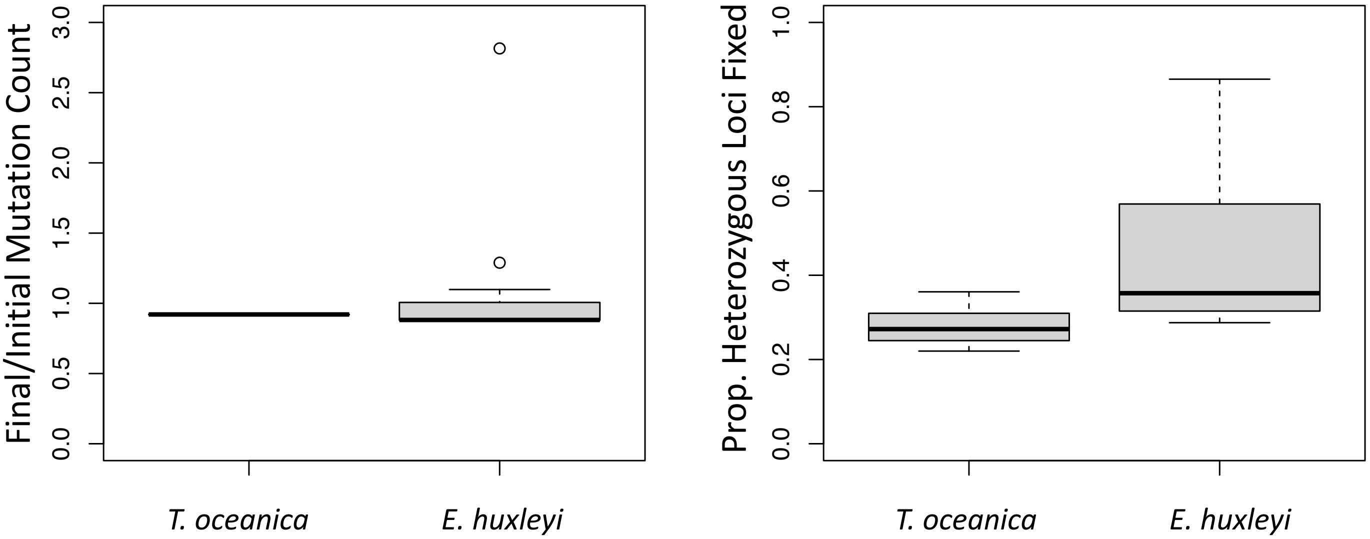

Figure S29. Heterozygosity in eukaryote genomes. We re-assembled the eukaryote reference genomes using breseq and identified 96,556 and 132,276 putatively heterozygous loci in *T. oceanica* and *E. huxleyi*, respectively. A) Mutations identified in evolutionary lineages were corrected based on reference to the re-assembled genome and predictions of heterozygosity, changing the number of mutations considered. For *T. oceanica* and most *E. huxleyi* genomes, this resulted in a modest decrease in the mutation count of approximately 8% and 12%, respectively. However, for three *E. huxleyi* genomes, many loci that were heterozygous in the reference genome became fixed in the evolved genomes, leading to increased numbers of mutations under consideration. B) For *T. oceanica* genomes, about 30% of ancestrally heterozygous loci fixed in each lineage. This value was much more variable for *E. huxleyi*, with a median value of 36% and a maximum of 87%..

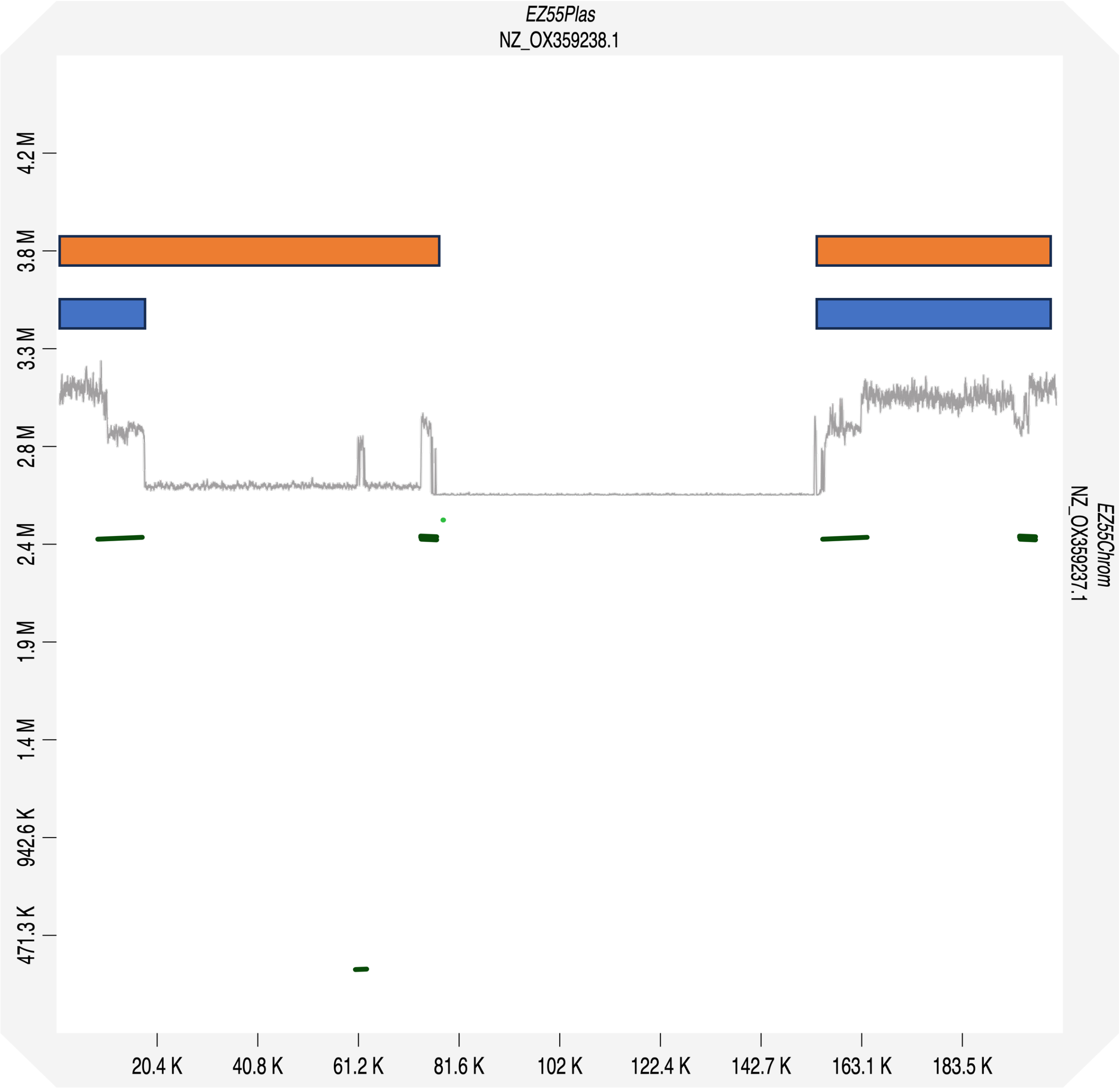

Figure S30. Homology between EZ55 chromosome and plasmid DNA sequences. Positions along the EZ55 main chromosome are represented on the y-axis, and positions along the plasmid are shown on the x-axis. Black dots indicate >95% homology between the paired sequences. Overlayed in light gray is a map of EZ55 plasmid coverage from LTPE428 (Fig. S6) scaled to match the x-axis, showing the erroneous mapping of chromosomal reads to deleted portions of the plasmid. The map also suggests potential targets for homologous recombination and insertion into the main chromosome that may explain the patterns of coverage and deletion observed. Blue bars indicate the approximate extent of the chromosome-insertion region, and orange bars represent the approximate extent of the reduced-size plasmid.

Data S1. (separate file)

Convergently evolved genes in phytoplankton genomes. “PCO2 Search” indicates whether the given gene passed our curation criteria in one or both of the pCO_2 treatments, which were analyzed separately; “Total Mutations” indicates the total number of unique mutations observed across all lineages at the indicated pCO_2 treatment; “Lineages Represented” indicates the number of replicately evolved lineages in which at least one mutation was observed for the given gene; “P Value” is the significance level of the comparison between total mutations observed in each pCO_2 treatment.

Data S2. (separate file)

Convergently evolved genes in *Alteromonas* genomes, binned by phytoplankton partner and pCO_2 treatment and curated by pCO_2 treatment. “PCO2 Search” indicates whether the given gene passed our curation criteria in one or both of the pCO_2 treatments, which were analyzed separately in each phytoplankton partner treatment; “Total Mutations” indicates the total number of unique mutations observed across all lineages at the indicated partner and pCO_2 treatment; “Lineages Represented” indicates the number of replicately evolved lineages in which at least one mutation was observed for the given gene; “P Value” is the significance level of the comparison between total mutations observed in each pCO_2 treatment.

Data S3. (separate file)

Convergently evolved genes in *Alteromonas* genomes, binned by phytoplankton partner and pCO_2 treatment and curated by phytoplankton partner. “Significant In” indicates with which phytoplankton partner(s) the gene passed our curation criteria; “Total Mutations” indicates the total number of unique mutations observed across all lineages for the indicated partner; “Lineages Represented” indicates the number of replicately evolved lineages in which at least one mutation was observed for the given gene; “P Values” indicate the significance level of the comparison between total mutations observed between the indicated phytoplankton pairings.
